# Finotonlimab (PD-1 inhibitor) plus bevacizumab (bevacizumab biosimilar) as first-tier therapy for late-stage hepatocellular carcinoma: a randomized phase 2/3 trial

**DOI:** 10.1038/s41392-025-02333-5

**Published:** 2025-08-06

**Authors:** Chuanhua Zhao, Yanqiao Zhang, Gang Wang, Jinfang Zheng, Weiqing Chen, Zheng Lu, Li Zhuang, Shanzhi Gu, Lei Han, Zhendong Zheng, Zujiang Yu, Yongsheng Yang, Hongmei Sun, Xiaoyong Wei, Ying Cheng, Hailan Lin, Bo Zhu, Guicheng Wu, Kaijian Lei, Wei Wang, Yuwen Wang, Kehe Chen, Ximing Xu, Cuiping Zheng, Yanzhi Bi, Sijuan Ding, Jingdong Zhang, Wei Li, Hailong Liu, Jun Wang, Xianling Liu, Yangfeng Du, Lianming Cai, Jingran Wang, Zhanxiong Luo, Baocai Xing, Jie Shen, Lin Yang, Jianbing Wu, Ou Jiang, Zhigang Peng, Xiuli Liu, Bangwei Cao, Liangfang Shen, Aibing Xu, Aimin Li, Shaojun Chen, Ting Fu, Jian Chen, Chuan Jin, Lei Zhang, Jun Lv, Chengwu Zhang, Xiaoman Zhang, Yu Wang, Huo Su, Qiang Zhou, Wenlin Gai, Liangzhi Xie, Jianming Xu

**Affiliations:** 1 Department of Oncology, The Fifth Medical Center, Chinese PLA General Hospital, Beijing, China; 2 Harbin Medical University Cancer Hospital, Harbin, China; 3 Baoji Central Hospital, Baoji, China; 4 Hainan General Hospital, Hainan, China; 5 Chongqing University Cancer Hospital, Chongqing, China; 6 The First Affiliated Hospital of Bengbu Medical University, Bengbu, China; 7 Yunnan Cancer Hospital, Yunnan, China; 8 Hunan Cancer Hospital, Hunan, China; 9 Affiliated Hospital of Jining Medical University, Jining, China; 10 General Hospital of Northern Theater Command, Shenyang, China; 11 The First Affiliated Hospital of Zhengzhou University, Zhengzhou, China; 12 The Second Norman Bethune Hospital of Jilin University, Jilin, China; 13 Jiamusi Cancer Hospital, Jiamusi, China; 14 Jiangxi Cancer Hospital, Jiangxi, China; 15 Jilin Cancer Hospital, Jilin, China; 16 Fujian Cancer Hospital, Fujian, China; 17 Xinqiao Hospital of Army Medical University, Xinqiao, China; 18 Chongqing University Three Gorges Hospital, Chongqing, China; 19 The Second People’s Hospital of Yibin, Yibin, China; 20 The Second Attached Hospital of Fujian Medical University, Fujian, China; 21 The Sixth People’s Hospital of Shenyang, Shenyang, China; 22 The People’s Hospital of Guangxi Zhuang Autonomous Region, Guangxi, China; 23 Renmin Hospital of Wuhan University, Hubei General Hospital, Hubei, China; 24 Wenzhou Central Hospital, Wenzhou, China; 25 Changzhou Cancer (Fourth People’s) Hospital, Changzhou, China; 26 The Central Hospital of Yongzhou, Yongzhou, China; 27 Liaoning Cancer Hospital&Institute, Liaoning, China; 28 The First Bethune Hospital of Jilin University, Jilin, China; 29 Chenzhou No.1 People’s Hospital, Chenzhou, China; 30 People’s Hospital of Hunan Province, Hunan, China; 31 The Second Xiangya Hospital of Central South University, Xiangya, China; 32 The First People’s Hospital of Changde City, Changde, China; 33 Ganzhou Cancer Hospital, Ganzhou, China; 34 Shijiazhuang People’s Hospital, Shijiazhuang, China; 35 Liuzhou People’s Hospital, Liuzhou, China; 36 Beijing Cancer Hospital, Beijing, China; 37 Nanjing Drum Tower Hospital, Nanjing, China; 38 Cancer Hospital Chinese Academy of Medical Sciences, Beijing, China; 39 The Second Affiliated Hospital of Nanchang University, Nanchang, China; 40 The Second People’s Hospital of Neijiang, Neijiang, China; 41 The First Affiliated Hospital of Guangxi Medical University, Guangxi, China; 42 The First People’s Hospital of Yichang, Yichang, China; 43 Beijing Friendship Hospital, Capital Medical University, Beijing, China; 44 XiangYa Hospital Central South University, XiangYa, China; 45 Nantong Tumor Hospital, Nantong, China; 46 Southern Medical University Integrated Traditional Chinese and Western Medicine, Guangzhou, China; 47 Liuzhou Workers’ Hospital, Liuzhou, China; 48 Zhuzhou Central Hospital, Zhuzhou, China; 49 Yantai Yuhuangding Hospital, Yantai, China; 50 Affiliated Cancer Hospital and Institute of Guangzhou Medical University, Guangzhou, China; 51 Sun Yat-sen Memorial Hospital, Sun Yat-sen University, Sun Yat-sen, China; 52 Beijing You’an Hospital, Capital Medical University, Beijing, China; 53 Zhejiang Provincial People’s Hospital, Zhejiang, China; 54 Beijing Engineering Research Center of Protein and Antibody, Sinocelltech Ltd, Beijing, China

**Keywords:** Gastrointestinal cancer, Gastrointestinal cancer

## Abstract

We aimed to assess the tolerability and efficacy of finotonlimab (an anti-programmed cell death protein-1 antibody) in combination with SCT510, a bevacizumab biosimilar, versus sorafenib in unresectable advanced HCC. This randomized phase 2 and 3 study (ClinicalTrials.gov, NCT04560894; Chinadrugtrials.org.cn, CTR20201976 and CTR20201974) was performed at 67 hospitals in China. HCC patients (*n* = 398) were included between 11 November 2020 and 28 September 2022. In phase 2, patients received intravenous finotonlimab (200 mg every 3 weeks) combined with SCT510 (15 mg/kg every 3 weeks). In phase 3, 346 patients were randomized (2:1) to either the finotonlimab plus SCT510 (dual-agent) group or the sorafenib group. The median follow-up time for the dual-agent therapy and sorafenib groups was 19.9 and 19.0 months, respectively. Median PFS, assessed by BICR according to RECIST 1.1, was significantly longer in the dual-agent group (7.1 months [95% confidence intervals {CI}: 6.1, 8.4]) than in the sorafenib group (2.9 months [95% CI: 2.8, 4.1]; stratified hazard ratio [HR]: 0.5, 95% CI: 0.38, 0.65, *p* < 0.0001). Median OS was also significantly longer in patients receiving finotonlimab plus SCT510 (22.1 months [18.6, not available]) than in those receiving sorafenib (14.2 months [95% CI: 10.2, 15.8]; HR: 0.60 [95% CI: 0.44, 0.81], *p* < 0.0008). Finotonlimab in combination with bevacizumab demonstrated favorable efficacy, in comparison to sorafenib, as a first-line treatment for unresectable HCC, with a manageable safety profile.

## Introduction

According to the 2022 GLOBOCAN, a globally recognized cancer database, the incidence of new cases of hepatocellular carcinoma (HCC) ranks as the fourth most commonly diagnosed cancer and the second leading cause of cancer-related death in China.^1^ Typically, HCC presents subtly, with over 70% of cases identified in intermediate or advanced stages. Furthermore, the overall recurrence rate within 5 years is ~70%.^2^ Most patients lose the opportunity for surgical intervention after recurrence, making systemic therapy the most commonly used treatment approach for unresectable HCC.

Sorafenib has long been the first-line standard for advanced HCC worldwide, despite offering a mOS of under one year.^3^ In a non-inferiority study, lenvatinib, which targets VEGFR, FGFR, and PDGFR, demonstrated an OS of 13.6 months compared with 12.3 months for sorafenib. Based on these results, lenvatinib became the second targeted therapy to receive approved.^4^ With the advancement of biological therapies, immune checkpoint inhibitors (ICIs) have demonstrated efficacy in treating HCC; however, the effectiveness of single-agent treatments for HCC remains limited. The involvement of vascular endothelial growth factor (VEGF) in modulating cancer immune responses indicates that targeting VEGF with antibodies could improve the efficacy of immunotherapy.^5^ The IMbrave150 study demonstrated that the combination of atezolizumab (a programmed death ligand [PD-L1] inhibitor) with bevacizumab (anti-VEGF antibody) for advanced HCC significantly improved survival and showed favorable safety.^6^

Although several phase III clinical trials have explored the synergistic potential of ICIs in conjunction with bevacizumab or tyrosine kinase inhibitors (TKIs) for the treatment of advanced HCC, only the combination of atezolizumab and bevacizumab had obtained approval from received Food and Drug Administration at that time.^6^ However, in China, no programmed cell death protein 1 (PD-1) inhibitor combined with anti-VEGF therapy had been approved for the treatment of advanced HCC at the onset of this trial. Consequently, the treatment options for Chinese patients are limited at the onset of this trial. Currently, sintilimab (PD-1 inhibitor) and bevacizumab biosimilar (anti-VEGF) (ORIENT-32 study) ^7^ and camrelizumab (PD-1 inhibitor) and rivoceranib (TKI) (CARES-310 study).^8^ have been approved in China. Meanwhile, although not yet approved in China, the guidelines have recommended the combination of ICIs as first-line therapy for late-stage liver cancer, such as HIMALAYA study^9^ and CheckMate - 9DW.^10^

Finotonlimab (SCT-I10A) is a humanized immunoglobulin G monoclonal antibody targeting PD-1 that has demonstrated antitumor activity in both murine models and patients.^11^ Finotonlimab has shown high efficacy and a favorable safety profile in several types of cancer.^12–15^ SCT510, a bevacizumab biosimilar, also developed by Sinocelltech Ltd., has received authorization for use in China in June 2023.^16^ Here, we present the findings from a phase II/III study assessing the efficacy and tolerability of finotonlimab and SCT510 in treating late-stage HCC.

## Results

### Study profile

**Fig. 1:**
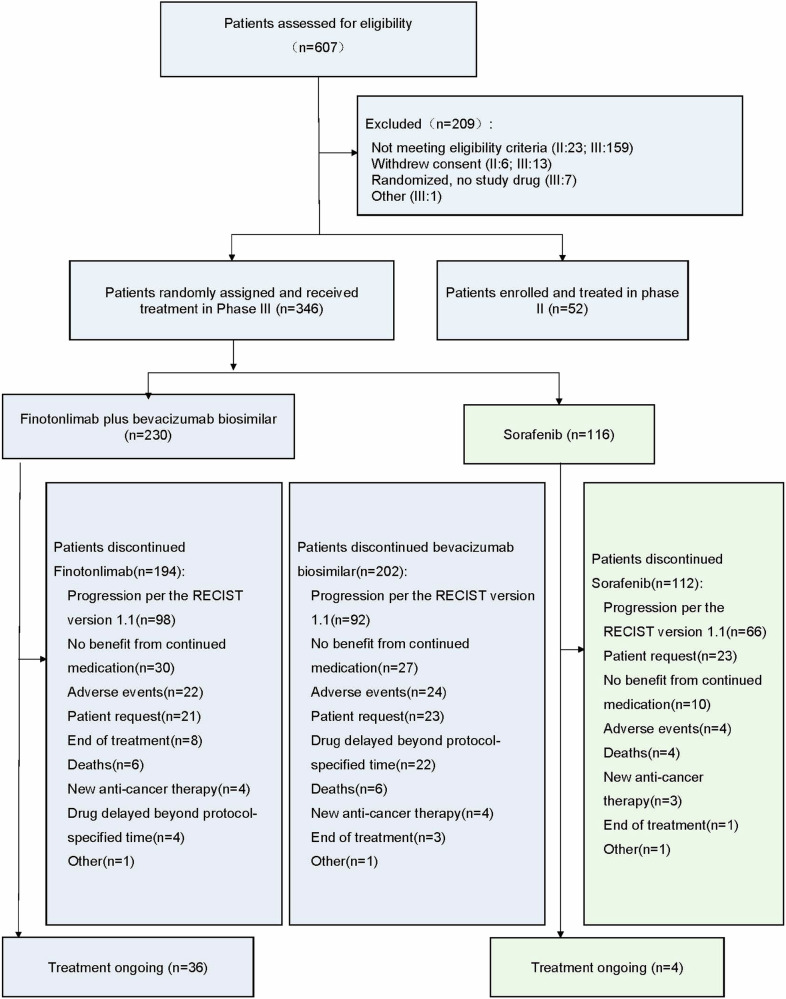
Trial profile. RECIST Response Evaluation Criteria in Solid Tumors

Between November 11, 2020 and September 28, 2022, 609 patients were screened (81 in phase II and 526 in phase III). After excluding 202 patients, those who participated in the randomization and received the study drug were 52 in phase II and 346 in phase III (Fig. 1).

### Efficacy and safety in the phase II study

As of November 2, 2023, the median follow-up duration is 32.2 months (95% confidence interval [CI]: 31.5, 33.1). Treatment-emergent adverse events (TEAEs) related to either drug occurred in 48 of the 52 patients, of which 23 were classified as grade 3 or higher. Serious AEs were reported in 17 patients, of which 14 were related to either drug, and two patients experienced drug-related deaths due to intestinal obstruction and hepatic failure, respectively. The ORR, as assessed by the investigators based on RECIST 1.1, was 26.9%. The mPFS was 8.4 months, whereas the mOS was 24.0 months. A phase II study demonstrated that the dual-agent has an acceptable safety profile and significant efficacy. The detailed results of the phase II study are presented in Supplementary Table 1, Supplementary Table 2, and Supplementary Table 3.

### Baseline characteristics of the groups in the phase III study

In the phase III, 346 patients received at least one dose of the study drugs: 230 patients in the finotonlimab plus SCT510 group (dual-agent group) and 116 patients in the sorafenib group (Fig. 1). At the data cut-off date (Nov 2, 2023), 36 patients in the dual-agent group and 4 patients in the sorafenib group were still receiving treatment. The two groups showed comparable baseline characteristics (Table 1).

**Table 1:** Baseline characteristics

	Finotonlimab plus SCT510 (*n* = 230)	Sorafenib (*n* = 116)
Age, Median (min, max), years	57.0 (30,79)	56.0 (29,77)
Sex, *n* (%)		
Male	197 (85.7)	104 (89.7)
Female	33 (14.3)	12 (10.3)
Eastern Cooperative Oncology Group performance status, *n* (%)		
0	105 (45.7)	53 (45.7)
1	125 (54.3)	63 (54.3)
Child-Pugh score, *n* (%)		
5	163 (70.9)	87 (75.0)
6	51 (22.2)	21 (18.1)
7	16 (7.0)	8 (6.9)
Barcelona Clinic Liver Cancer stage, *n* (%)		
B	46 (20.0)	23 (19.8)
C	184 (80.0)	93 (80.2)
Hepatitis B, *n* (%)	208 (90.4)	100 (86.2)
Hepatitis C, *n* (%)	10 (4.3)	7 (6.0)
α fetoprotein, *n* (%)		
≥400 ng/mL	109 (47.4)	55 (47.4)
<400 ng/mL	121 (52.6)	61 (52.6)
Macrovascular invasion, extrahepatic metastasis, or both	184 (80.0)	91 (78.4)
Macrovascular invasion, *n* (%)	85 (37.0)	51 (44.0)
Extrahepatic metastasis, *n* (%)	142 (61.7)	69 (59.5)
Previous local treatment, *n* (%)	71 (30.9)	41 (35.3)
Sum of target lesion diameters, Median (min, max), mm	97.0 (11,291.7)	85.7 (13,256.0)
Platelet count at baseline, Median (min, max), ×10^9^/L	164 (77,602)	156 (77,366)

### Efficacy in the phase III study

**Fig. 2:**
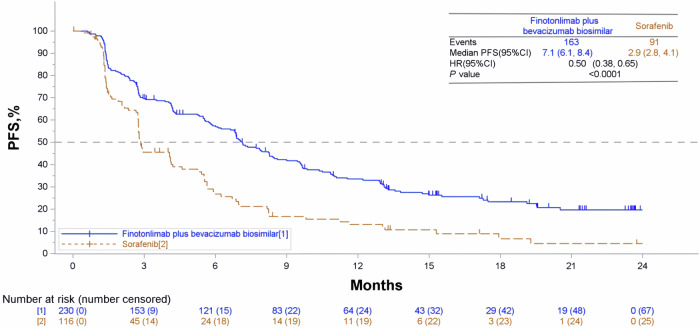
Kaplan-Meier curves of PFS assessed by BICR. BICR Blinded Independent Central Review, PFS progression-free survival, HR hazard ratio; The horizontal dashed line shows the mPFS

The median follow-up period was 19.7 months (19.9 months for the dual-agent group and 19.0 months for the sorafenib group). Disease progression or death occurred in 163 patients (70.9%) in the dual-agent group and in 91 patients (78.4%) in the sorafenib group. The mPFS was significantly prolonged in patients who received the dual-agent in comparison to patients receiving sorafenib. The mPFS was 7.1 months (95% CI: 6.1, 8.4) in the dual-agent and 2.9 months (95% CI: 2.8, 4.1) in the sorafenib group. (stratified hazard ratio [HR] 0.5, 95% CI: 0.38, 0.65, *p* < 0.0001, Fig. 2).

**Fig. 3:**
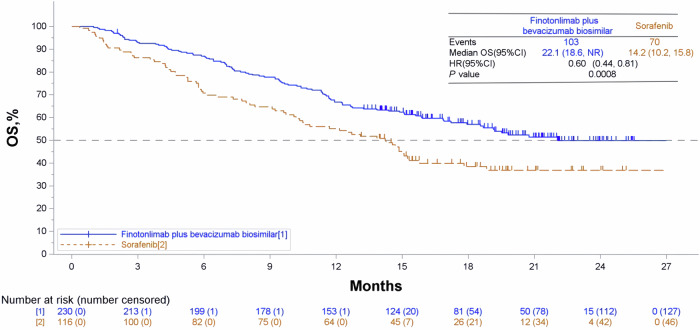
Kaplan-Meier curves of OS. OS Overall survival, HR hazard ratio, The horizontal dashed line shows the mOS

The mOS was 22.1 months (95% CI: 18.6, not available) in the dual-agent group and 14.2 months (95% CI: 10.2, 15.8) in the sorafenib group (HR: 0.60, 95% CI: 0.44, 0.81, *p* < 0.0008) (Fig. 3). The dual-agent significantly prolonged OS and reduced the risk of death by 40% compared with sorafenib. The 1-, 1.5-, and 2-years survival rates of the dual-agent group versus the sorafenib group were 66.8% vs 55.2%, 57.0% vs 38.5%, and 49.9% vs 36.8%, respectively. The dual-agent group demonstrated superior survival rates compared to the sorafenib group, suggesting a sustained survival benefit.

The mPFS, based on RECIST 1.1, was 7.9 months in the dual-agent group and 3.0 months in the sorafenib group (HR 0.48, *p* < 0.0001). These findings were consistent with the results obtained by the BICR, both of which indicated that the dual-agent prolonged PFS in comparison to sorafenib (Supplementary Fig. 1). In the response-assessable population, the confirmed ORRs assessed by the BICR based on RECIST1.1 were 32.8% for the dual-agent and 4.3% for sorafenib, with disease control rates of 78.6% and 60.3%, respectively (Table 2). The confirmed ORRs assessed by the investigators, also based on RECIST 1.1, were 28.7% for the dual-agent and 6.9% for sorafenib, with corresponding disease control rates of 75.2% and 59.5%, respectively. Additionally, the confirmed ORRs assessed by BICR based on the modified RECIST (mRECIST) were 33.2% and 4.3%for the dual-agent and sorafenib, respectively (Table 2). BICR and the assessments using both RECIST and mRECIST performed by the investigators consistently demonstrated that the dual-agent significantly reduced the tumor burden in patients.

**Table 2:** Confirmed response assessed by BICR

	Recist v1.1		mRecist v1.1	
	Finotonlimab plus SCT510 (*n* = 229)	Sorafenib (*n* = 116)	Finotonlimab plus SCT510 (*n* = 229)	Sorafenib (*n* = 116)
Best of response *n* (%)				
Complete response	0	0	0	0
Partial response	75 (32.8)	5 (4.3)	76 (33.2)	5 (4.3)
Stable disease	105 (45.9)	65 (56.0)	103 (45.0)	65 (56.0)
Progressive disease	41 (17.9)	27 (23.3)	42 (18.3)	27 (23.3)
Not evaluable	8 (3.5)	19 (16.4)	8 (3.5)	19 (16.4)
Objective response rate (95%CI), %	32 (26.7,39.2)	4.3 (1.4,9.8)	33.2 (27.1,39.7)	4.3 (1.4,9.8)
*P* value	<0.0001		<0.0001	
Disease control rate (95% CI), %	78.6 (72.7, 83.73)	60.3 (50.8, 69.3)	78.2 (72.3, 83.3)	60.3 (50.8, 69.3)
*P* value	0.0002		0.0003	
Median duration of response (month), 95%CI	NA (14.5, NA)	NA (4.5, NA)	NA (12.4, NA)	NA (4.5, NA)

*RECIST* Response Evaluation Criteria in Solid Tumors, *BICR* Blinded Independent Central Review

**Fig. 4:**
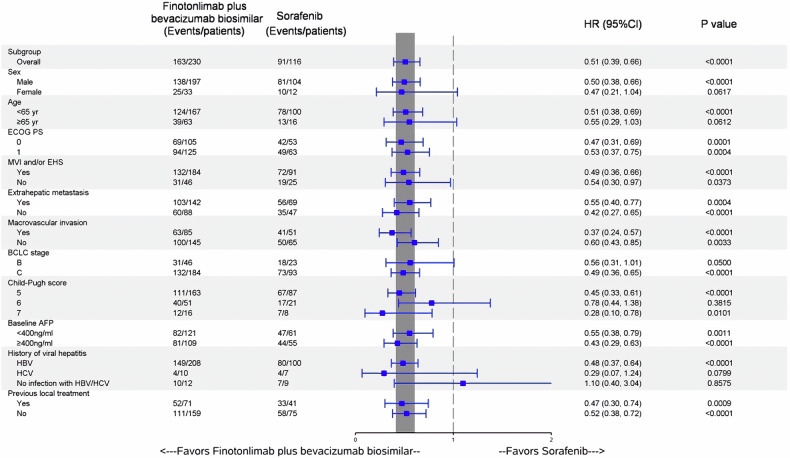
BICR-assessed progression-free survival in prespecified subgroups. ECOG Eastern Cooperative Oncology Group, MVI Macrovascular invasion, EHS extrahepatic metastasis, BCLC Barcelona Clinic Liver Cancer, AFP α-fetoprotein, HR hazard ratio

**Fig. 5:**
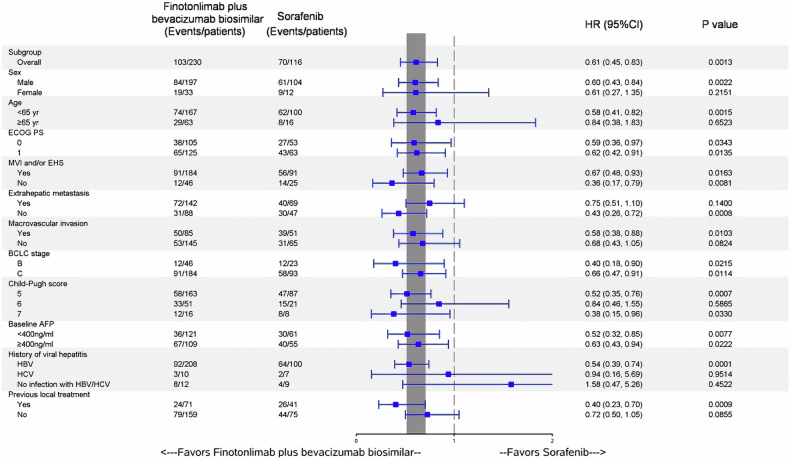
Overall survival in prespecified subgroups. ECOG Eastern Cooperative Oncology Group, MVI Macrovascular invasion, EHS extrahepatic metastasis, BCLC Barcelona Clinic Liver Cancer, AFP α-fetoprotein, HR hazard ratio

Subgroup analyses of OS and PFS were also performed, with the results illustrated in Figs. 4,5. In most of the pre-specified subgroups, the dual-agent showed superior benefits in terms of both PFS and OS, in comparison to sorafenib.

In the dual-agent group, the median time to deterioration (mTTD) in global health status was 9.7 months (95% CI: 7.2, 17.5), compared with 4.9 months (95% CI: 2.8, 7.0) in the sorafenib group (HR: 0.57, 95% CI: 0.39, 0.84). The mTTD in physical function was not reached in both groups (HR 0.79, 95% CI, 0.48, 1.3). For role functioning deterioration, the mTTD was not reached in the dual-agent group in contrast to 7.0 months in the sorafenib group (HR: 0.54, 95% CI: 0.34, 0.84, Supplementary Figs. 2-4). Quality of life remained relatively stable in the dual-agent group before cycle 21 (Supplementary Figs. 5-7). After cycle 21, less than 15% of the patients remained for patient-reported outcome (PRO) analysis.

### Safety in the phase III study

The safety analysis was performed in 346 patients who had at least one dose of the study drugs: 230 in the dual-agent group and 116 in the sorafenib group. The median durations of treatment with finotonlimab, SCT510, and sorafenib were 30.1, 29.2, and 12.2 weeks, respectively, and the relative dose intensities were 92.3%, 91.7%, and 81.5%, respectively.

TEAEs occurred in 223 patients (97.0%) in the dual-agent group and in 108 patients (93.1%) in the sorafenib group. The incidence of TEAEs of ≥10% for either drug is shown in Table 3. Among these, 121 patients (52.6%) in the dual-agent group and 44 patients (37.9%) in the sorafenib group experienced grad ≥ 3 TEAEs. The incidence of ≥5% grade ≥3TEAEs included decreased platelet counts [dual-agent: 16(7.0%); sorafenib: 3(2.6%)], proteinuria [dual-agent: 15(6.5%); sorafenib: 1(0.9%)], palmoplantar erythema syndrome [dual-agent: 0; sorafenib: 8 (6.9%)], and hypertension [dual-agent: 20 (8.7%); sorafenib: 5 (4.3%)]. The incidence of ≥10% TEAEs related to either drug is detailed in Supplementary Table 6. A higher proportion of serious AE was observed in the dual-agent group than in the sorafenib group [67 (29.1%) vs 15 (12.9%)]. TEAEs related death were reported in seven patients (3.0%) in the dual-agent group [one due to hepatic encephalopathy, one due to intracranial hemorrhage, one due to paraneoplastic syndrome, one due to heart failure, one due to upper gastrointestinal hemorrhage, and two unexplained deaths] and in two patients (3.0%) in the sorafenib group [one due to an unknown cause and one due to ruptured hepatocellular carcinoma]. A total of 121 patients receiving the dual-agent experienced immune-related AEs, of which 28 (12.2%) were grade ≥ 3, as shown in Supplementary Table 7. Additionally, 23 patients (10.0%) experienced immune-related AEs that led to the interruption, 11 patients (4.8%) experienced events that resulted in the discontinuation of finotonlimab, and there were no immune-related AEs that resulted in death.

**Table 3:** TEAE occurring in 10% or more in either group

	Finotonlimab plus SCT510 (*n* = 230) *N* (%)		Sorafenib (*n* = 116) *N* (%)	
	Any grade	Grade 3–5	Any grade	Grade 3–5
Any event	223 (97.0)	121 (52.6)	108 (93.1)	44 (37.9)
Proteinuria	106 (46.1)	15 (6.5)	29 (25.0)	1 (0.9)
Decreased platelet count	91 (39.6)	16 (7.0)	36 (31.0)	3 (2.6)
Weight decreased	58 (25.2)	2 (0.9)	34 (29.3)	0
COVID-19	58 (25.2)	7 (3.0)	9 (7.8)	0
Increased aspartate aminotransferase	61 (26.5)	3 (1.3)	26 (22.4)	3 (2.6)
Increased blood bilirubin	57 (24.8)	5 (2.2)	28 (24.1)	0
Decreased white blood cell count	53 (23.0)	9 (3.9)	25 (21.6)	5 (4.3)
Hypoalbuminemia	50 (21.7)	0	14 (12.1)	0
Anemia	48 (20.9)	5 (2.2)	14 (12.1)	3 (2.6)
Hypertension	47 (20.4)	20 (8.7)	18 (15.5)	5 (4.3)
Increased alanine aminotransferase	45 (19.6)	1 (0.4)	28 (24.1)	2 (1.7)
Decreased neutrophil count	40 (17.4)	10 (4.3)	18 (15.5)	5 (4.3)
Hypokalemia	39 (17.0)	7 (3.0)	15 (12.9)	3 (2.6)
Decreased appetite	36 (15.7)	3 (1.3)	13 (11.2)	0
Hypothyroidism	36 (15.7)	0	3 (2.6)	0
Diarrhea	32 (13.9)	2 (0.9)	43 (37.1)	3 (2.6)
Amylase increased	29 (12.6)	2 (0.9)	13 (11.2)	0
Hyponatremia	28 (12.2)	6 (2.6)	6 (5.2)	0
Pruritus	27 (11.7)	0	4 (3.4)	0
Upper respiratory infection	27 (11.7)	2 (0.9)	4 (3.4)	0
Pyrexia	27 (11.7)	0	13 (11.2)	0
Elevated blood pressure	25 (10.9)	7 (3.0)	6 (5.2)	2 (1.7)
Asthenia	25 (10.9)	2 (0.9)	15 (12.9)	2 (1.7)
Abdominal distension	24 (10.4)	1 (0.4)	11 (9.5)	1 (0.9)
Gamma-glutamyltransferase increased	23 (10.0)	5 (2.2)	11 (9.5)	0
Lipase increased	23 (10.0)	2 (0.9)	9 (7.8)	2 (1.7)
Increased blood alkaline phosphatase	23 (10.0)	0	8 (6.9)	0
Rash	15 (6.5)	0	20 (17.2)	3 (2.6)
Hypophosphatemia	13 (5.7)	0	13 (11.2)	0
Increased blood lactate dehydrogenase	5 (2.2)	0	13 (11.2)	0
Palmar-plantar erythrodysaesthesia syndrome	3 (1.3)	0	44 (37.9)	8 (6.9)
Alopecia	0	0	17(14.7)	0

At least one TEAE resulted in the interruption of either drug in 123 patients (53.5%) in the dual-agent and in 49 patients (42.2%) in the sorafenib treatment. In the sorafenib group, 39 patients (33.6%) experienced TEAEs that resulted in dose reductions. Additionally, at least one TEAE leading to the discontinuation of either drug was shown in 30 patients (13.0%) in the dual-agent group and in 5 patients (4.3%) in the sorafenib group (Supplementary Table 8). The most common reason for discontinuation was upper gastrointestinal hemorrhage (dual-agent group: 10 (4.3%); sorafenib group: 0).

## Discussion

This study demonstrated that dual-agent involving finotonlimab plus SCT510 in patients with advanced HCC significantly prolonged survival, extended the disease progression-free period and significantly reduced tumor burden. Subgroup analyses further substantiated the consistent clinical benefits observed in this patient group”. The baseline features of the study population closely resembled those reported in similar studies. Notably, the percentage of patients with a Child-Pugh score of 7 was the highest in this study at 7%, compared with other similar studies in which such patients were either not included or had a lower representation.^7^ The results of Child-Pugh B7 patients are presented in Supplementary Tables 9 and Table 10. The proportion of patients with AFP levels ≥400 ng/mL was also highest in this study, at 47.4%, compared with other studies such as IMbrave150 China,^17^ Orient32 ^7^, and CARES-310^8^ indicating a worse baseline level in the patients of this study.

In this study, the PFS for the dual-agent group was 7.1 months, with an HR of 0.50. By comparison, the IMbrave150^17^ and Orient32^7^ studies, which investigated the combination of ICIs with anti-VEGF therapies for late-stage HCC, reported mPFS of 6.9 months (HR: 0.59) and 4.6 months (HR: 0.56), respectively. Additionally, outcomes from the CARES-310^8^, examining the combination of ICIs with small-molecule TKIs, were also available, with an mPFS of 5.6 months and an HR of 0.52. In the IMbrave150 study,^18^ which included a broader global population, the HRs for mPFS were higher compared to studies primarily involving Chinese populations, such as ORIENT32 and the present study. This discrepancy may be partially explained by differences in baseline characteristics, particularly the varying prevalence of hepatitis B virus (HBV) infection, a significant etiological factor for HCC in China. In this study, ~90% of the enrolled patients were HBV-infected, aligning with findings from the ORIENT32 study.^7,17^ By contrast, the HBV infection rate was lower in the CARES-310 study^8^(76%) and significantly lower in the IMbrave150 global study^17^ (49%). Published evidence suggests that patients with HBV-associated liver cancer may derive greater benefit from ICIs compared to those with HCC caused by non-viral factors, such as non-alcoholic steatohepatitis (NASH).^19,20^ Supporting this, the OS subgroup analysis of the Leap 002 study showed an HR of 0.75 for HBV-infected patients, compared to 0.95 for non-HBV-infected patients.^21^ While preclinical studies have suggested potential biological differences in tumor microenvironment between HBV-associated HCC and NASH-related HCC, current clinical evidence from randomized trials has not demonstrated statistically significant differences in immunotherapy efficacy based on etiological subtypes.

The mOS in the agent group and the sorafenib group in this study were 22.1 months and 14.2 months (HR: 0.60), respectively. Despite the poor tumor and baseline characteristics of the patients enrolled, the dual-agent demonstrated a significant survival benefit compared to sorafenib. Furthermore, the ORR was 32% in the dual-agent group, significantly higher than 4.3% in the sorafenib group, highlighting the enhanced efficacy of the dual-agent. These findings emphasize the potential of the dual-agent as a promising treatment option for late-stage hepatocellular carcinoma. For context, other major trials reported similar trends in survival and response rates. The IMbrave150 study showed an OS of 19.2 months versus 13.4 months (HR:0.66),^18^ the Orient32 study reported OS as not reached versus 10.4 months (HR: 0.57),^7^ and the CARES-310 study showed an OS of 23.8 months versus 15.2 months (HR:0.64).^22^ ORRs from these trials included 21% versus 4% in Orient32,^7^ 29.8% versus 11.3% in IMbrave150,^18^ and 26.8% versus 5.9% in CARES-310^22^. However, cross-trail comparisons should be interpreted caution.

In phase II of the study, the common AEs associated with finotonlimab or SCT510 were similar to those observed with PD-1 inhibitor monotherapy or bevacizumab, with no new safety findings detected. The incidences of related AEs and SAEs were 92.3% and 26.9%, respectively, similar to those in ORIENT-32 study (TRAEs: 92.0%, TRSAEs: 25.0%)^7^ but lower than those in the camrelizumab plus apatinib study (TRAEs: 98.6%, TRSAEs: 32.9%).^23^ This suggests that ICIs may have a better safety profile when combined with large-molecule anti-angiogenic drugs than when combined with small-molecule TKIs. In phase III, adverse AEs occurred at similar rates in both groups: 97.0% of patients in the dual-agent group and 93.1% in the sorafenib group experienced AEs. In this study, the incidence of ≥grade 3 TRAEs was 42.6%, comparable to that observed in the Orient-32.^7^ and IMbrave150 studies.^17^ (both ~40.0%), and significantly lower than that reported in the CARES-310 study (81.0%).^8^ This further indicates that combining ICIs and anti-VEGF therapy may be safer than the combination of ICIs and TKIs. The most ≥3 AEs in this study included decreased platelet count, proteinuria, and hypertension in the dual-agent group, whereas palmoplantar erythema syndrome was the most frequently observed AE in the sorafenib group. AEs leading to death associated with SCT510 in the dual-agent arm included intracranial hemorrhage and upper gastrointestinal hemorrhage, both of which are known side effects of the drug and are similar to those in comparable studies, with no new safety signals identified. The most common AE leading to discontinuation of either study drug in the dual-agent group was upper gastrointestinal bleeding, similar to the findings in the Orient32 study, ^7^ whereas patients with HCC, which is usually associated with cirrhosis, are at a higher risk for gastroesophageal variceal bleeding. The most frequent immune-related AEs reported included hypothyroidism, skin toxicity, liver toxicity, and amylase elevation, which were consistent with the characteristics of immune-related AEs in HCC. Although the percentage of amylase elevation was high, the patients were primarily asymptomatic amylase elevation, with an actual incidence of pancreatitis of only 0.9%. The study design called for the detection of changes in amylase markers in every cycle to monitor the risk of pancreatic toxicity. However, amylase elevation is also susceptible to multiple causes. Overall, immune-related toxicity did not increase the risk of unintended AEs. Bleeding is a known adverse reaction associated with bevacizumab. In this study, the percentage of bleeding-related events was 26.7% in the dual-agent group (vs. 20.7% in the sorafenib group), a result comparable to the 25.2% reported in the IMbrave150 study. The combination regimen demonstrated no significant increase in bleeding risk for patients.

The study had some limitations: Firstly, the current investigation was a multicenter trial performed exclusively within China; hence, the generalizability of its findings to other ethnic populations has yet to be determined. Secondly, in the IMbrave150 study, patients were required to undergo endoscopy before enrollment to rule out grade 3 esophageal varices,^17^ whereas this study did not mandate endoscopy before enrollment. Instead, the decision to perform endoscopy was left to the investigator based on the individual patient’s condition, which may put patients at high risk of bleeding. Thirdly, the study’s open-label design, which was mitigated by the establishment of an Independent Data Monitoring Committee (IDMC) to reduce bias in efficacy and PFS assessments, may still be susceptible to biases in the implementation of the clinical trials and AE management. Finally, sorafenib was the only standard treatment option available at the start of the study. However, some patients in the sorafenib group had subsequent targeted immune dual-agent therapy after discontinuing the study, which may have confounded the comparison of survival outcomes between the treatment arms.

In conclusion, finotonlimab combined with SCT510 demonstrated significant efficacy as a first-line therapy for HCC. The primary endpoints of PFS and OS met the prespecified test criteria, with primary analyses demonstrating statistically and clinically significant benefits. The safety profile of the dual-agent was manageable, with no unanticipated or uncontrollable safety signals identified compared with similar marketed products.

## Materials and methods

### Study design

This phase 2 and 3 study was performed at 67 centers in China between 11 November 2020 and 28 September 2022. This clinical trial included phase II single-arm study and a phase III randomized controlled study. The phase II study served as a safety run-in period, and focused on assessing the tolerability of treatment with finotonlimab plus SCT510. The phase III study was a randomized, controlled trial in which patients were randomly assigned to either finotonlimab plus SCT510 (dual-agent) or sorafenib. This study was carried out in alignment with the ethical principles of the Declaration of Helsinki, Good Clinical Practice guidelines, and local regulatory requirements. All patients provided written informed consent.

### Patients

Key inclusion criteria were as follows: patients aged 18 years or older; ECOG physical status score of 0–1; Diagnosed with HCC.^24^ without prior systemic treatment; Barcelona Clinical Liver Cancer Staging (BCLC) stage C or stage B unfit for surgery and/or local therapy; a Child-Pugh score of ≤7; Presence of at least one measurable lesion as defined by RECIST 1.1, and adequate hematologic and organ function.

Main exclusion criteria included previous surgical or local therapy 4 weeks before the study; with CNS metastases or carcinomatous meningitis; bleeding tendency, high bleeding risk, or coagulopathy; portal vein cancerous embolism involving the main trunk and right and left branches, or cancerous embolism in the inferior vena cava or cardiac involvement; ruptured esophageal or gastric varices within 6 months prior to the first study drug therapy; active, known or suspected autoimmune disease; concomitant serious medical illness; and acute or chronic active hepatitis B or hepatitis C infection with hepatitis B virus (HBV) DNA > 2000 IU/ml or 10^4^ copies/ml; or Hepatitis C virus RNA > 10^3^ copies/ml. The complete criteria are detailed in the protocol.

### Randomisation and masking

In the phase III study, eligible patients were assigned in a 2:1 ratio by stratified blocked randomization to receive either finotonlimab plus SCT510 (dual-agent) or sorafenib. The randomization stratification factors were Eastern Cooperative Oncology Group (ECOG) performance status (0 vs 1), baseline alpha-fetoprotein (AFP) level (<400 ng/mL vs ≥400 ng/mL), and having macrovascular invasion and/or extrahepatic metastases (no vs. yes). Aaeduhe study surgical or local tn IIDMC was formed to reduce the risk of bias associated with open-label status and to maintain data integrity, especially for the monitoring of trial data, including interim analysis.

### Procedure

In both the phase II and randomized phase III experimental group, patients received finotonlimab 200 mg intravenously over 60 min, followed by SCT510 at 15 mg/kg, every 3 weeks. SCT510 was initially infused over 90 min, with subsequent infusions reduced to 60 and 30 min if tolerated. Patients who experience delayed or interrupted administration of finotonlimab or SCT510 in dual-agent due to adverse events (AE), may continue to receive finotonlimab or SCT510 monotherapy. No dose adjustments were permitted for finotonlimab. However, for SCT510, dose adjustments were required based on body weight. If there is a change of 10% or more from the body weight, the dose should be recalculated. Patients in the control group had sorafenib 400 mg orally twice daily. Dosage adjustments for sorafenib were permitted up to two times: from 400 mg twice daily to 400 mg once daily, and subsequently to 400 mg every other day.

Patients were treated until any of the following conditions were met: disease progression, unmanageable toxicity, commencement of new oncologic treatment, death, or loss to follow-up. After disease progression, use of the study drug could continue based on patient consent and the investigator’s assessment regarding the clinical benefit. The treatment would be discontinued if the investigator determined there was no further clinical benefit, intolerable toxicity occurred, the patient or investigator decided to discontinue treatment, death occurred, or loss to follow-up occurred.

Tumor was examined by CT or MRI at baseline and every 6 weeks after the subject received the first dose of medication, continuing every 9 weeks for up to 48 weeks. Tumor response was assessed by both the investigator and the BICR according to RECIST1.1 criteria, and also by the BICR according to mRECIST criteria. The EORTC QLQ-C30 and EORTC QLQ-HCC18 scales were also assessed in the phase III study. Safety assessments included thorough vital signs, physical examinations, laboratory tests, and any AE as evaluated by the investigator in alignment with the National Cancer Institute (NCI) Common Terminology Criteria for Adverse Events (CTCAE 5.0). These assessments were collected from the time the subject received the first dose of the drug until 90 days after the last dose or prior to the commencement of new oncologic treatment.

### Outcomes

In phase II, the efficacy endpoints included the objective response rate (ORR) according to RECIST 1.1 criteria, duration of response, disease control rate, progression-free survival, overall survival, pharmacokinetics, and immunogenicity in patients receiving finotonlimab plus SCT510.

In phase III, the primary endpoints were progression-free survival (PFS) as evaluated by central review (BICR) according to RECIST 1.1 and overall survival. Secondary endpoints included 1-, 1.5-, and 2-year survival rates; PFS according to RECIST 1.1; objective response rate, duration of response, and disease control rate as assessed by both BICR and the investigator according to RECIST 1.1; PFS, objective response rate, disease control rate and duration of response as assessed by BICR according to mRECIST. Quality of life was also evaluated, with a post-hoc analysis conducted using EORTC QLQ-C30. Other endpoints included PD-L1 expression levels with efficacy and prognosis, safety, and the pharmacokinetics and immunogenicity of finotonlimab plus SCT510.

### Statistical analysis

This phase II single-arm part served as a safe introductory period, with a planned enrollment of 50–60 patients, was a crucial step in understanding the initial efficacy and tolerability. After the last subject completed the initial efficacy evaluation, the study transitioned into a phase III randomized controlled part.

The phase III part was designed to enroll approximately 342 patients, employed a superiority parallel design, with dual primary endpoints: OS and PFS. One PFS analysis was performed, after 252 PFS events, providing 85% power to detect an HR of 0.67 favoring finotonlimab plus SCT510 compared with sorafenib; and two OS analyses (one interim and a final) were planned at the occurrence of 127 (50% of 253 total expected) deaths and 253 deaths, also providing a power of 85% to detect an HR of 0.67 favoring finotonlimab plus SCT510. The type I error rate was controlled at two-sided 0.05. Using a fixed-sequential procedure, PFS and OS were analyzed with a two-sided *α* of 0.05, respectively. The PFS was analysed first; if the null hypothesis for PFS was not rejected, the trial was terminated. Conversely, if the test result of PFS was significant, the interim analysis of OS was planned concurrently with the final PFS analysis. The Lan-DeMets spending function was utilized to estimate the O’Brien-Fleming boundary for the interim and final analyses of OS. The primary analysis for PFS was conducted upon the occurrence of 254 PFS events, with a significance boundary of 0.05. Meanwhile, the interim OS was analyzed after 173 deaths (68% of the expected 253), with a significance boundary of 0.01343.

Analyses in the phase II were descriptive. In phase III, the efficacy analyses for OS and PFS were based on the mITT population (all patients treated with at least one dose of the study drug). Efficacy was evaluated in the response-evaluable population with measurable baseline tumors, and tolerability was analyzed in the mITT population.

We employed the Kaplan-Meier method to estimate mPFS, utilizing the Brookmeyer–Crowley method to calculate the associated 95% CIs. Greenwood’s formula and log-log transformations were applied to estimate PFS rates along with their corresponding 95% CIs. PFS was compared between groups to calculate *p*-values using a log-rank test, and the stratified Cox proportional hazards model was employed to calculate the HRs and the 95% CI in the phase III. OS was calculated using the same methodology as for PFS. Prespecified subgroup analyses and forest plots were generated for the primary endpoints of PFS and OS.

The ORR and disease control rate were calculated, and their 95% CIs were derived using the Clopper Pearson method, rate difference and *p*-value for comparisons between groups were calculated using the stratified Cochran–Mantel–Haenszel test. Time to deterioration in health-related quality-of-life outcomes was analyzed using the methodology applied to other time-to-event endpoints. Changes of Least Squares Mean score from baseline in health-related quality-of-life outcomes were analyzed with the Mixed Models for Repeated Measures.

An IDMC was established to conduct the prespecified interim analysis and monitor safety data throughout the study period. Statistical analyses were performed with SAS version 9.4.

## Supplementary information

Phase II-III Trial-Supplementary_Materials

Protocol of the clinical trial

Statistical analysis plan of a clinical trial

## Data Availability

The authors also declare that the data supporting the findings of this study are available within the main manuscript or the supplementary material. Correspondence and requests for materials should be addressed to L.X. (LX@sinocelltech.com).
