# Supplementary material for: Finotonlimab (PD-1 inhibitor) plus bevacizumab (bevacizumab biosimilar) as first-tier therapy for late-stage hepatocellular carcinoma: a randomized phase 2/3 trial
Source: Signal Transduct Target Ther. 2025 Aug 6;10:249. doi: 10.1038/s41392-025-02333-5 (PMC12329032; doi:10.1038/s41392-025-02333-5)
Supplement: Supplementary file 1 — Phase II-III Trial-Supplementary_Materials [file 41392_2025_2333_MOESM1_ESM.docx]

Supplementary Materials for

Finotonlimab Plus Bevacizumab as First-tier Therapy for Late-Stage Hepatocellular Carcinoma: a Randomized Phase 2/3 Trial

Chuanhua Zhao1#, Yanqiao Zhang2#, Gang Wang3, Jinfang Zheng4, Weiqing Chen5, Zheng Lu6, Li Zhuang7, Shanzhi Gu8, Lei Han9, Zhendong Zheng10, Zujiang Yu11,Yongsheng Yang12, Hongmei Sun13, Xiaoyong Wei14, Ying Cheng15, Hailan Lin16, Bo Zhu17, Guicheng Wu18, Kaijian Lei19, Wei Wang20, Yuwen Wang21, Kehe Chen22, Ximing Xu23, Cuiping Zheng24, Yanzhi Bi25, Sijuan Ding26, Jingdong Zhang27, Wei Li28, Hailong Liu29, Jun Wang30, Xianling Liu31, Yangfeng Du32, Lianming Cai33, Jingran Wang34, Zhanxiong Luo35, Baocai Xing36, Jie Shen37, Lin Yang38, Jianbing Wu39, Ou Jiang40, Zhigang Peng41, Xiuli Liu42, Bangwei Cao43, Liangfang Shen44, Aibing Xu45, Aimin Li46, Shaojun Chen47,Ting Fu48, Jian Chen49, Chuan Jin50, Lei Zhang51, Jun Lv52, Chengwu Zhang53, Xiaoman Zhang54, Yu Wang54, Huo Su54, Qiang Zhou54, Wenlin Gai54, Liangzhi Xie54 , Jianming Xu

Correspondence to: [jmxu2003@163.com](mailto:jmxu2003@163.com)

**This PDF file includes:**

Supplementary Figures. 1 to 7

Supplementary Tables 1 to 10

Supplementary Fig 1. Kaplan-Meier curves of PFS assessed by investigator

BICR: Blinded Independent Central Review; PFS: progression-free survival; HR: hazard ratio; The horizontal dashed line shows the mPFS


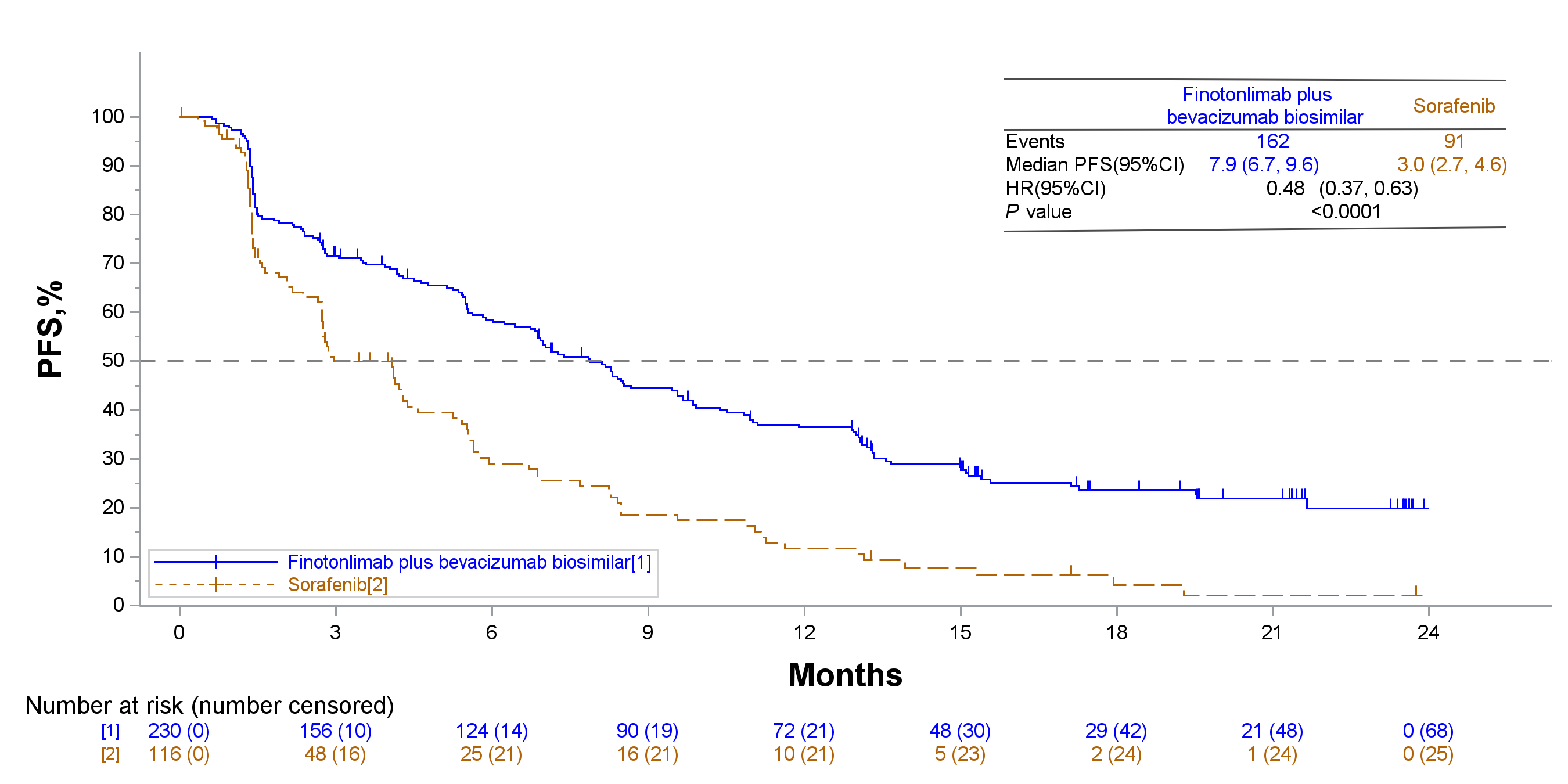


Supplementary Fig 2. Kaplan-Meier curves of EORTC QLQ-C30 TTD Estimates in GHS Domains


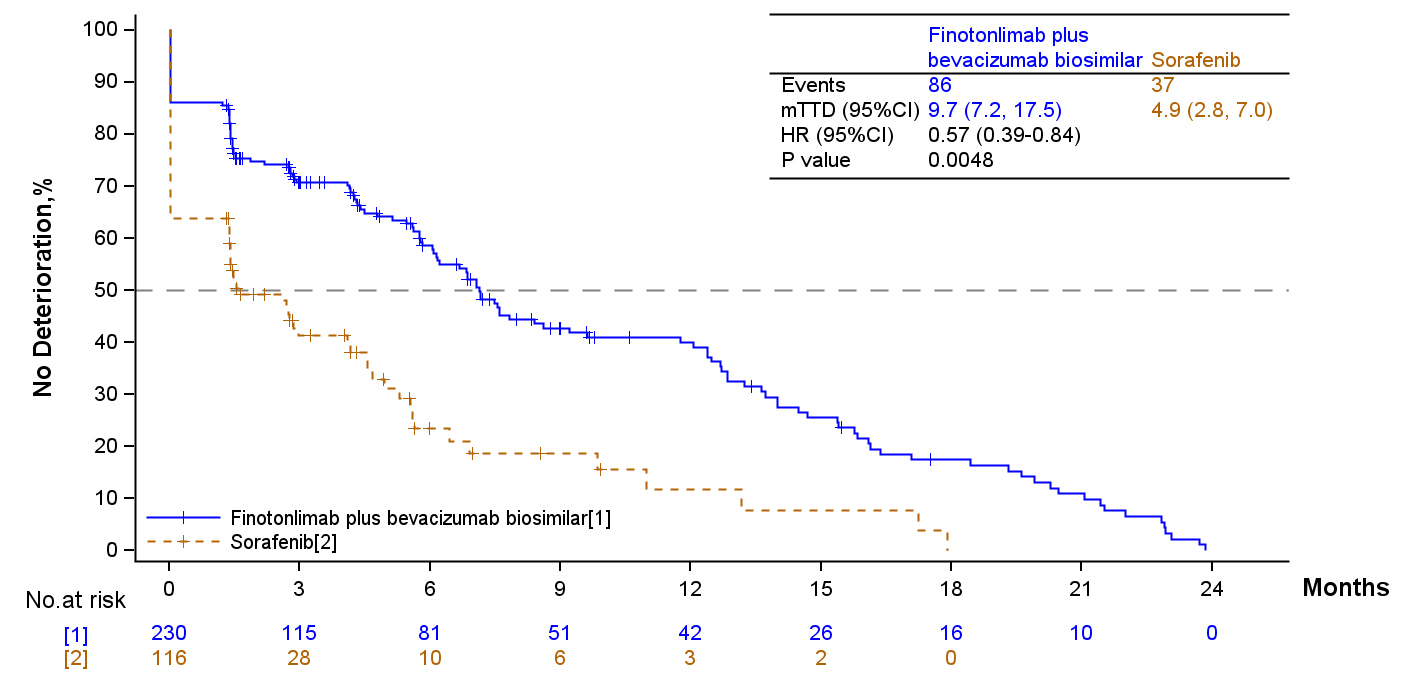


Supplementary Fig 3. Kaplan-Meier curves of EORTC QLQ-C30 TTD Estimates in Physical Functioning Domains


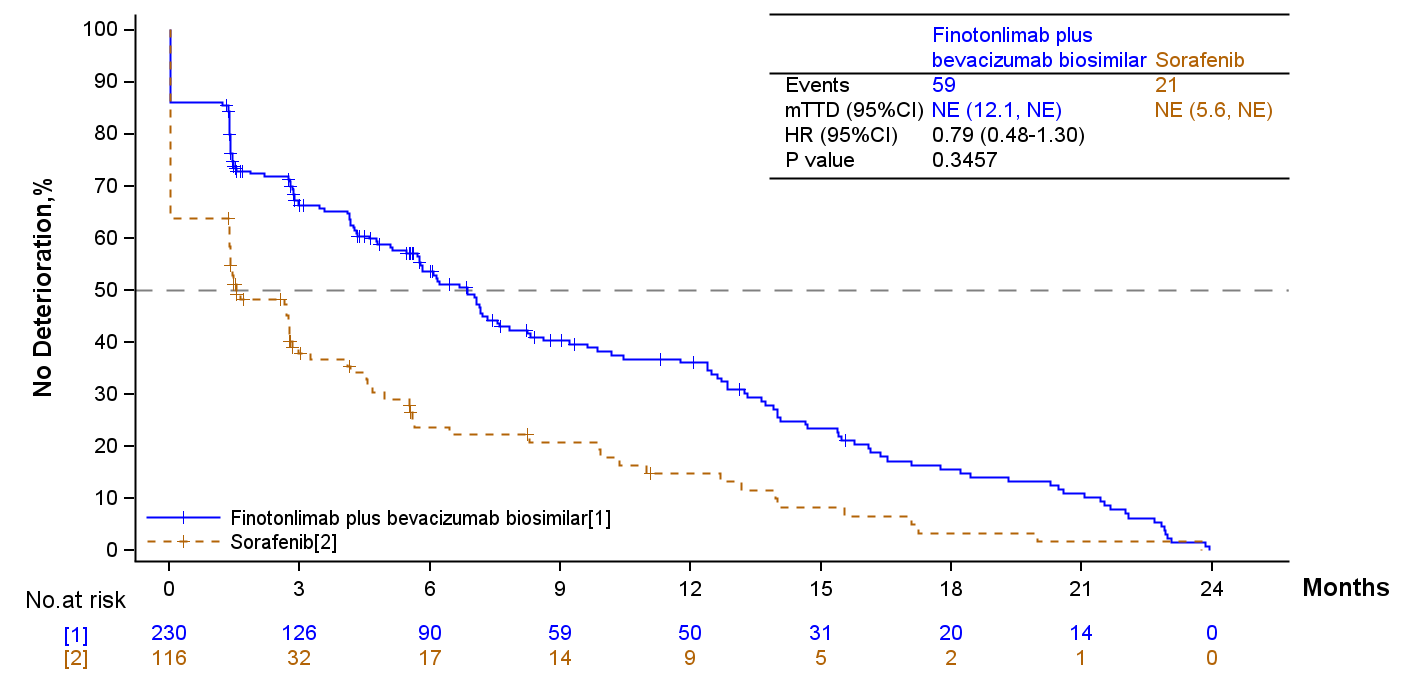


**Supplementary Fig 4**. **Kaplan-Meier curves of EORTC QLQ-C30 TTD Estimates in Role Functioning Domains**


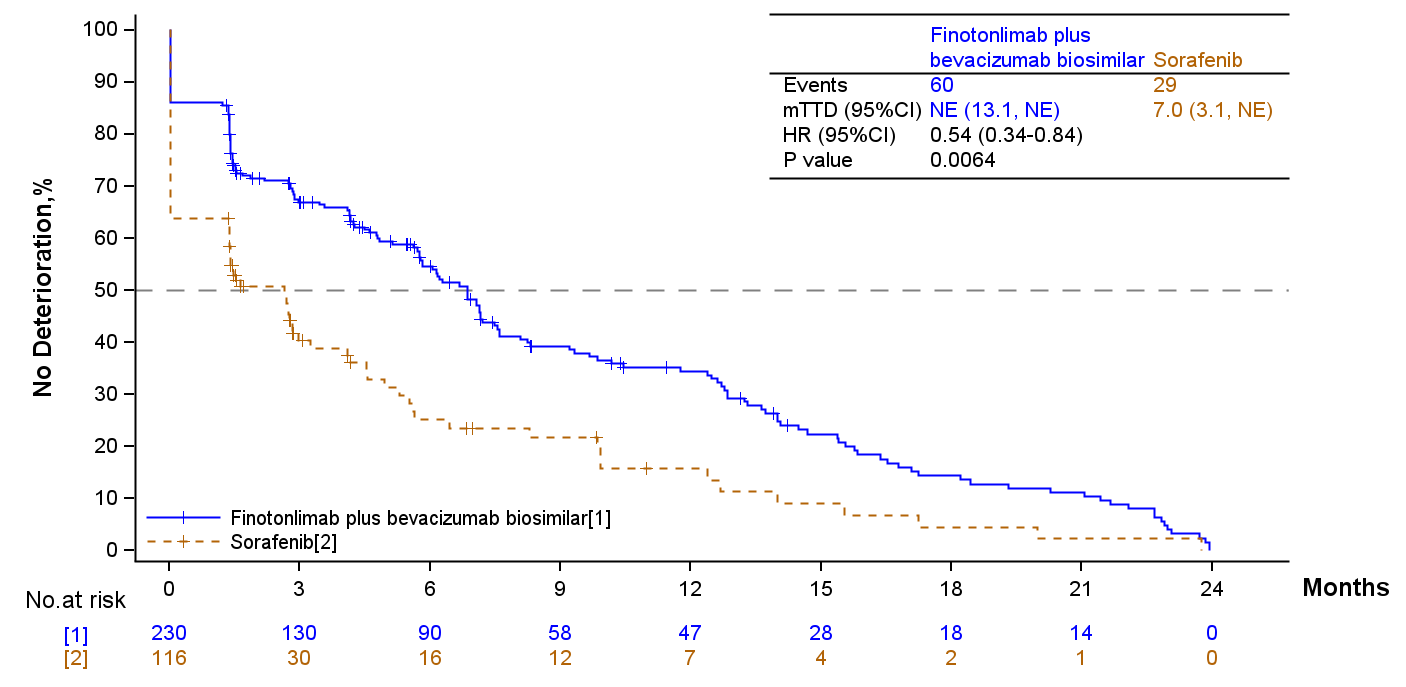


**Supplementary Fig 5. EORTC QLQ-C30: Physical Functioning**


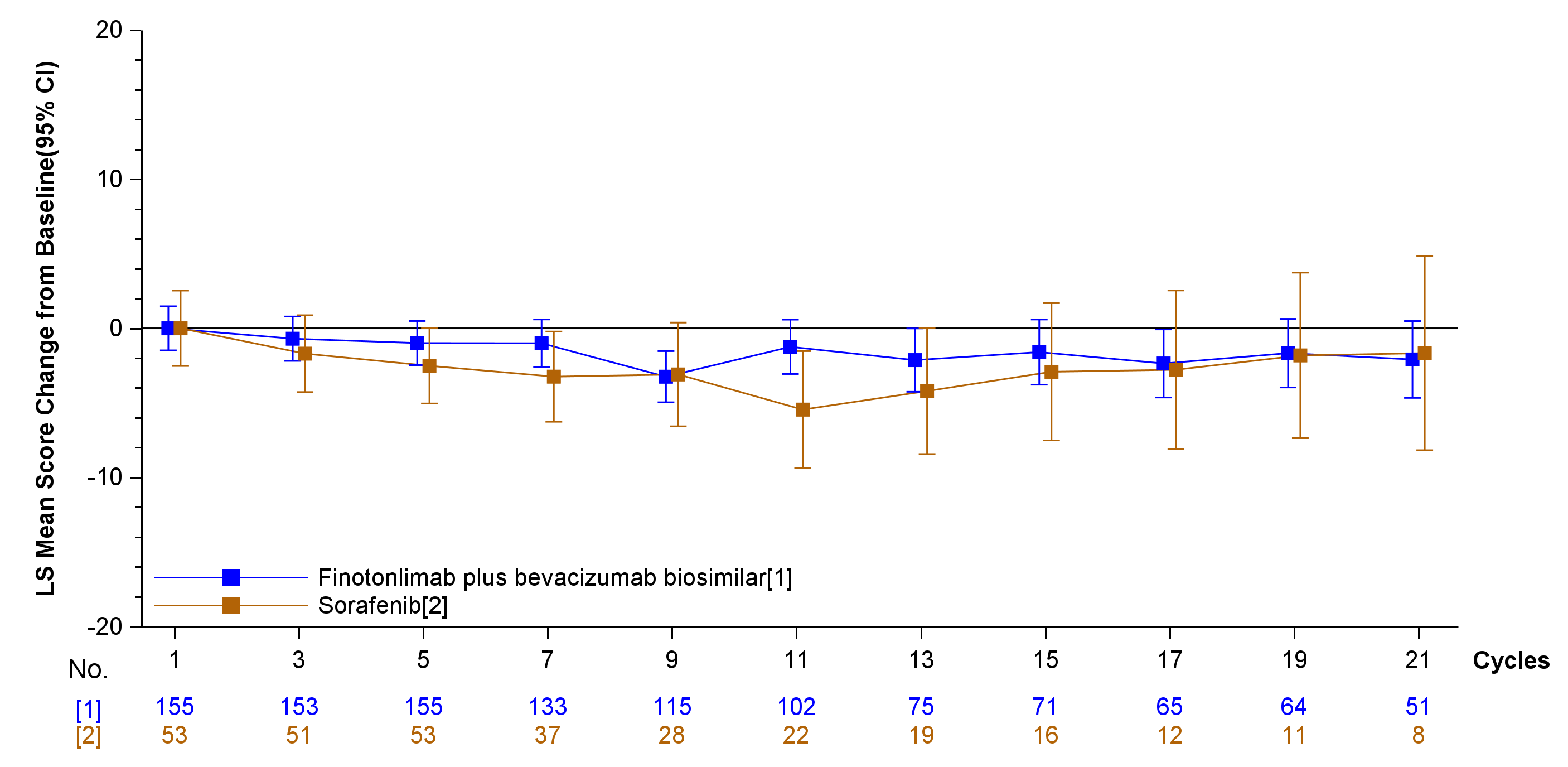


**Supplementary Fig 6. EORTC QLQ-C30: Role Functioning**


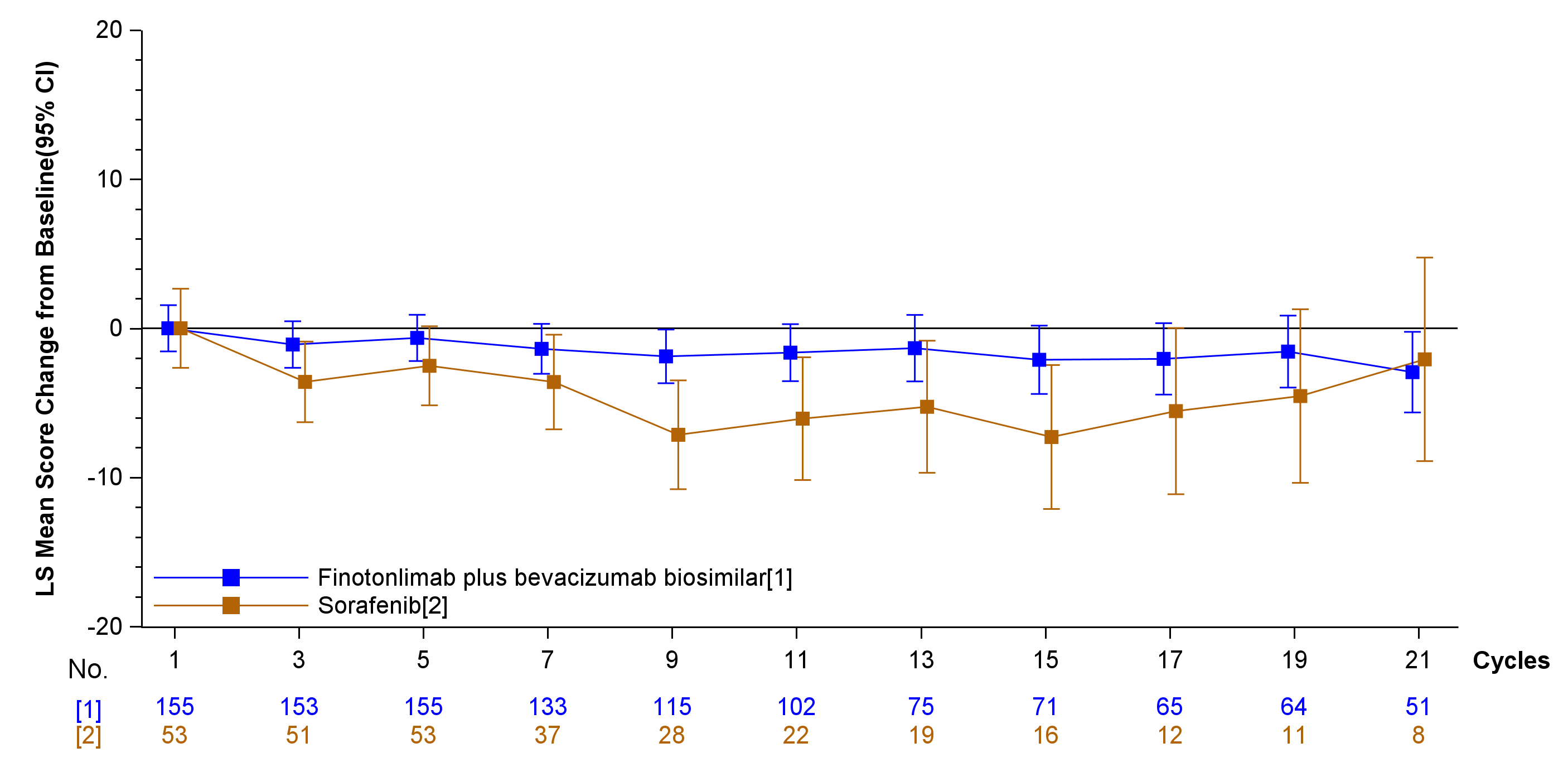


**Supplementary Fig 7** **Changes from baseline in scores for EORTC QLQ-C30 (quality of life, physical functioning, role functioning) by treatment cycle**


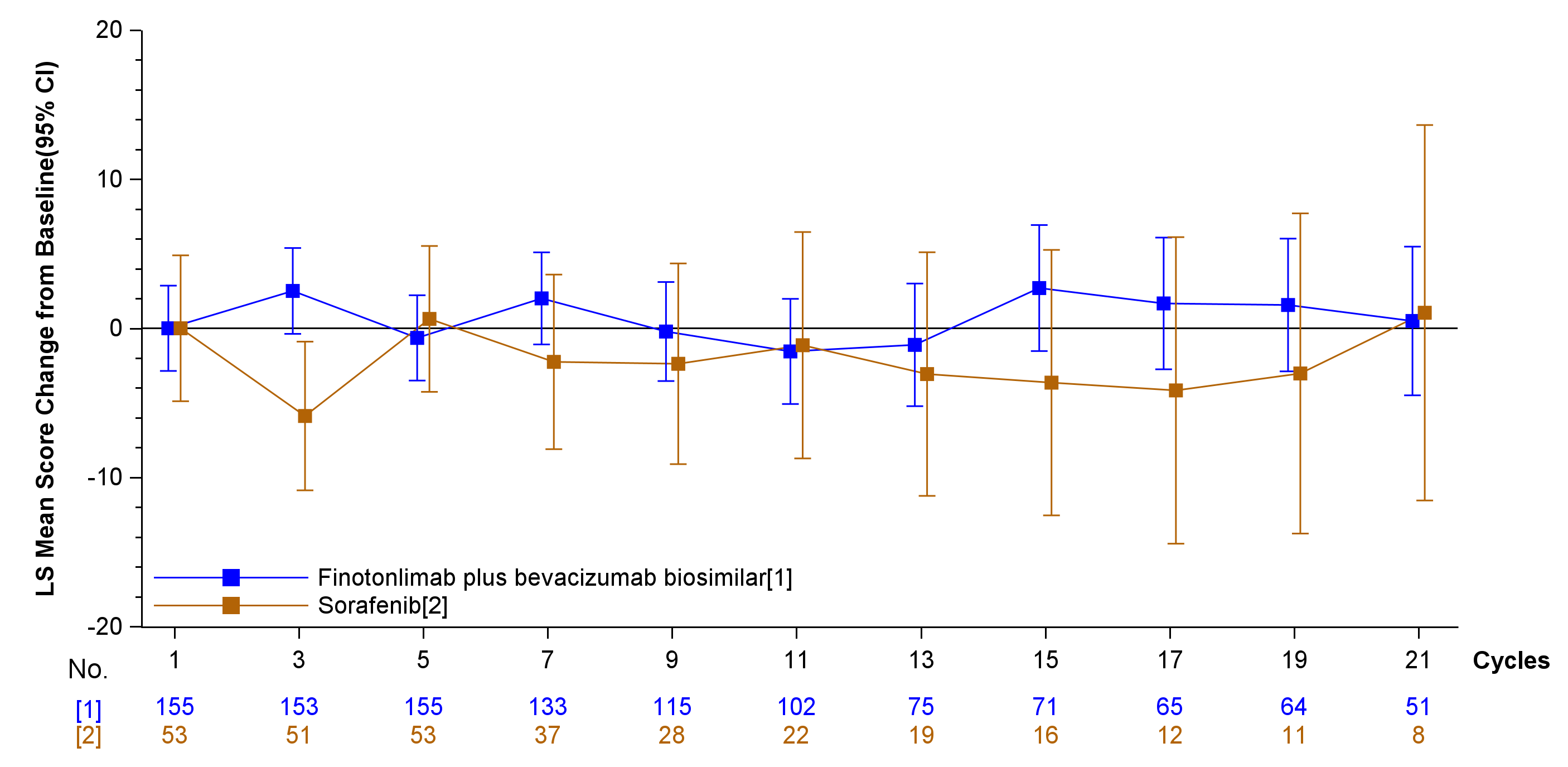


Supplementary Table 1.Efficacy outcomes in phase II

|  | **Finotonlimab plus bevacizumab biosimilar**  **(n=52)** |
| --- | --- |
| Best overall response, n(%) |  |
| Complete response | 0 |
| Partial response | 14 (26.9) |
| Stable disease | 28 (53.8) |
| Progressive disease | 9 (17.3) |
| Not evaluable | 1 (1.9) |
| Objective response rate (ORR), n(%) | 14 (26.9) |
| 95%CI | 15.57, 41.02 |
| Disease control rate (DCR), n(%) | 42 (80.8) |
| 95%CI | 67.47, 90.37 |
| Median duration of response (month),95%CI | 13.7 (9.7,NA) |
| Median progression-free survival (month), 95%CI | 8.4 (4.0,12.3) |
| Median overall survival (month), 95%CI | 24.0 (15.6,33.6) |

Supplementary Table 2.AE summary in phase II

|  | **Finotonlimab plus bevacizumab biosimilar**  **(n=52)**  **N (%)** |
| --- | --- |
| Treatment-emergent adverse events (TEAE) | 50 (96.2) |
| TEAE related to either drug (TRAE) | 48 (92.3) |
| CTCAE ≥3 TEAE | 28 (53.8) |
| CTCAE ≥3 TRAE | 23 (44.2) |
| Treatment-emergent serious adverse events (TESAE) | 17 (32.7) |
| TESAE related to either drug (TRSAE) | 14 (26.9) |
| TEAE caused death | 5 ( 9.6) |
| TRAE caused death | 2 ( 3.8) |
| TEAE leading to interrupt to either drug | 36 (69.2) |
| TRAE leading to interrupt to either drug | 33 (63.5) |
| TEAE leading to discontinue to either drug | 8 (15.4) |
| TRAE leading to discontinue to either drug | 8 (15.4) |

CTCAE: Common Terminology Criteria for Adverse Events

**Supplementary Table 3. Treatment-emergent adverse events with incidence≥10% in the safety set of phase II**

|  | **Total(n=52)** | **Grade1-2** | **Grade3** | **Grade4** |
| --- | --- | --- | --- | --- |
| Any | 50 (96.2) | 22 (42.3) | 21 (40.4) | 2 (3.8) |
| Platelet count decreased | 32 (61.5) | 25 (48.1) | 6 (11.5) | 1 (1.9) |
| Aspartate aminotransferase increase | 23 (44.2) | 21 (40.4) | 2 (3.8) | 0 |
| Proteinuria | 22 (42.3) | 19 (36.5) | 3 (5.8) | 0 |
| Weight decreased | 15 (28.8) | 15 (28.8) | 0 | 0 |
| White blood cell count decreased | 15 (28.8) | 12 (23.1) | 3 (5.8) | 0 |
| Blood bilirubin increased | 15 (28.8) | 15 (28.8) | 0 | 0 |
| Hypoproteinaemia | 15 (28.8) | 15 (28.8) | 0 | 0 |
| Neutrophil count decreased | 14 (26.9) | 13 (25.0) | 1 (1.9) | 0 |
| Anaemia | 13 (25.0) | 10 (19.2) | 3 (5.8) | 0 |
| Alanine aminotransferase increased | 12 (23.1) | 12 (23.1) | 0 | 0 |
| Hypertension | 11 (21.2) | 7 (13.5) | 4 (7.7) | 0 |
| Gamma-glutamyltransferase increased | 10 (19.2) | 8 (15.4) | 2 (3.8) | 0 |
| Asthenic conditions | 10 (19.2) | 9 (17.3) | 1 (1.9) | 0 |
| Arthralgia | 9 (17.3) | 9 (17.3) | 0 | 0 |
| Amylase increased | 8 (15.4) | 8 (15.4) | 0 | 0 |
| Blood pressure increased | 8 (15.4) | 7 (13.5) | 1 (1.9) | 0 |
| Rash | 8 (15.4) | 8 (15.4) | 0 | 0 |
| Fever | 8 (15.4) | 8 (15.4) | 0 | 0 |
| Lipase increased | 7 (13.5) | 7 (13.5) | 0 | 0 |
| Nausea | 7 (13.5) | 6 (11.5) | 1 (1.9) | 0 |
| Decreased appetite | 7 (13.5) | 7 (13.5) | 0 | 0 |
| Cough | 7 (13.5) | 7 (13.5) | 0 | 0 |
| Blood thyroid stimulating hormone increased | 6 (11.5) | 6 (11.5) | 0 | 0 |
| Abdominal pain | 6 (11.5) | 6 (11.5) | 0 | 0 |
| Hyponatraemia | 6 (11.5) | 4 (7.7) | 2 (3.8) | 0 |
| Hypokalaemia | 6 (11.5) | 4 (7.7) | 2 (3.8) | 0 |

**Supplementary Table** **4.** **New anti-tumor therapy in phase III**

|  | Finotonlimab plus bevacizumab biosimilar (n=230) | Sorafenib  (n=116) |
| --- | --- | --- |
| New anti-tumor therapy，n(%) | 131 (57.0) | 79 (68.1) |
| Immunotherapy | 44 (19.1) | 41 (35.3) |
| Targeted therapy | 91 (39.6) | 54 (46.6) |
| Chemotherapy | 13 (5.7) | 6 (5.2) |
| Local therapy | 36 (15.7) | 27 (23.3) |
| Radiotherapy | 8 (3.5) | 8 (6.9) |
| Others | 45 (19.6) | 34 (29.3) |

**Supplementary Table 5. AE summary in phase III**

|  | Finotonlimab plus bevacizumab biosimilar (n=230) n (%) | Sorafenib  (n=116)  n (%) |
| --- | --- | --- |
| Treatment-emergent adverse events (TEAE) | 223 (97.0) | 108 (93.1) |
| TEAE related to either drug (TRAE) | 211 (91.7) | 105 (90.5) |
| Treatment-emergent serious adverse events (TESAE) | 67 (29.1) | 15 (12.9) |
| TESAE related to either drug (TRSAE) | 46 (20.0) | 7 ( 6.0) |
| TEAE caused death | 7 ( 3.0) | 2 ( 1.7) |
| TRAE caused death | 3 ( 1.3) | 0 |
| TEAE leading to interrupt to either drug | 123 (53.5) | 49 (42.2) |
| TRAE leading to interrupt to either drug | 94 (40.9) | 46 (39.7) |
| TEAE leading to discontinue to either drug | 30 (13.0) | 5 ( 4.3) |
| TRAE leading to discontinue to either drug | 27 (11.7) | 3 ( 2.6) |

**Supplementary Table 6. Treatment-related adverse events occurring in 10%or more of patients in either group.**

|  | Finotonlimab plus bevacizumab biosimilar  （n=230）  N (%) | | Sorafenib  (n=116)  N (%) | |
| --- | --- | --- | --- | --- |
|  | Any grade | Grade 3-5 | Any grade | Grade 3-5 |
| Any event | 195(84.8) | 66(28.7) | 101(87.1) | 31(26.7) |
| Proteinuria | 103(44.8) | 15( 6.5) | 27(23.3) | 1( 0.9) |
| Decreased platelet count | 76(33.0) | 15( 6.5) | 30(25.9) | 1( 0.9) |
| Increased aspartate aminotransferase | 54(23.5) | 3( 1.3) | 23(19.8) | 3( 2.6) |
| Increased blood bilirubin | 49(21.3) | 4( 1.7) | 26(22.4) | 0 |
| Hypertension | 43(18.7) | 18( 7.8) | 18(15.5) | 5( 4.3) |
| Increased alanine aminotransferase | 43(18.7) | 1( 0.4) | 25(21.6) | 2( 1.7) |
| Decreased white blood cell count | 41(17.8) | 7( 3.0) | 22(19.0) | 5( 4.3) |
| Hypothyroidism | 35(15.2) | 0 | 2( 1.7) | 0 |
| Decreased neutrophil count | 32(13.9) | 9( 3.9) | 14(12.1) | 5( 4.3) |
| Hypoalbuminemia | 28(12.2) | 0 | 9( 7.8) | 0 |
| Amylase increased | 28(12.2) | 2( 0.9) | 12(10.3) | 0 |
| Anemia | 27(11.7) | 4( 1.7) | 9( 7.8) | 1( 0.9) |
| Pruritus | 24(10.4) | 0 | 4( 3.4) | 0 |
| Elevated blood pressure | 23(10.0) | 6( 2.6) | 5( 4.3) | 1( 0.9) |
| Diarrhea | 20( 8.7) | 2( 0.9) | 41(35.3) | 2( 1.7) |
| Decreased appetite | 19( 8.3) | 1( 0.4) | 12(10.3) | 0 |
| Asthenia | 19( 8.3) | 2( 0.9) | 15(12.9) | 2( 1.7) |
| Weight decreased | 17( 7.4) | 1( 0.4) | 18(15.5) | 0 |
| Rash | 12( 5.2) | 0 | 19(16.4) | 3( 2.6) |
| Palmar-plantar erythrodysaesthesia  syndrome | 3( 1.3) | 0 | 44(37.9) | 8( 6.9) |
| Alopecia | 0 | 0 | 17(14.7) | 0 |

**Supplementary Table 7. Immune-related adverse events (irAE)**

|  | Finotonlimab plus bevacizumab biosimilar (n=230) | |
| --- | --- | --- |
| irAEs subclassification | Any grade | Grade 3 or more |
| Any irAE | 121(52.6) | 28(12.2) |
| Hypothyroidism | 45(19.6) | 0 |
| Hyperthyroidism | 9(3.9) | 0 |
| Hyperglycemia or diabetes | 7(3.0) | 2( 0.9) |
| Other thyroid diseases | 3(1.3) | 0 |
| Hypophysitis | 2(0.9) | 1( 0.4) |
| Thyroiditis | 1(0.4) | 0 |
| Immune-related skin adverse reactions | 42(18.3) | 4( 1.7) |
| Increased amylase | 15(6.5) | 2( 0.9) |
| Lipase elevation | 9(3.9) | 1( 0.4) |
| Pancreatitis | 2(0.9) | 1( 0.4) |
| Immune-related hepatitis | 18(7.8) | 7( 3.0) |
| Immune-related diarrhea and colitis | 9(3.9) | 3( 1.3) |
| Immune-related thrombocytopenia | 9(3.9) | 7( 3.0) |
| Immune-related pneumonia | 6(2.6) | 1( 0.4) |
| Immuno-related nephritis | 3(1.3) | 0 |
| Inflammatory arthritis | 3(1.3) | 0 |
| Myalgia | 1(0.4) | 0 |
| Immune-related myocarditis | 2(0.9) | 0 |

**Supplementary Table** **8. TEAE leading to discontinue to either drug**

|  | Finotonlimab plus bevacizumab biosimilar (n=230) n(%) | Sorafenib (n=116) n(%) |
| --- | --- | --- |
| TEAE leading to discontinue to either drug | 30(13.0) | 5( 4.3) |
| Upper gastrointestinal hemorrhage | 10( 4.3) | 0 |
| Heart failure | 2( 0.9) | 0 |
| Colitis | 2( 0.9) | 0 |
| Decreased platelet count | 2( 0.9) | 0 |
| Increased blood bilirubin | 2( 0.9) | 0 |
| Hematemesis | 1( 0.4) | 0 |
| Acute pancreatitis | 1( 0.4) | 0 |
| Ulcerative colitis | 1( 0.4) | 0 |
| Pancreatitis | 1( 0.4) | 0 |
| Esophageal Variceal Bleeding | 1( 0.4) | 0 |
| Hepatic encephalopathy | 1( 0.4) | 0 |
| lacunar cerebral infarction | 1( 0.4) | 0 |
| Myasthenia gravis | 1( 0.4) | 0 |
| Intracranial hemorrhage | 1( 0.4) | 0 |
| Myocardial infarction | 1( 0.4) | 0 |
| Paraneoplastic syndrome | 1( 0.4) | 0 |
| Type 1 diabetes | 1( 0.4) | 0 |
| Hypopituitarism | 1( 0.4) | 0 |
| Immune-mediated lung disease | 1( 0.4) | 0 |
| Drug Eruption | 1( 0.4) | 0 |
| Aortic thrombosis | 1( 0.4) | 0 |
| Increased aspartate aminotransferase | 0 | 2( 1.7) |
| Increased alanine aminotransferase | 0 | 2( 1.7) |
| Death | 0 | 1( 0.9) |
| Ruptured liver cancer | 0 | 1( 0.9) |
| Gastric ulcer hemorrhage | 0 | 1( 0.9) |

**Supplementary Table 9. Comparison of efficacy between two groups with Child-Pugh score of 7**

|  | Finotonlimab plus bevacizumab biosimilar  (n=16) | Sorafenib  (n=8) |
| --- | --- | --- |
| Complete response | 0 | 0 |
| Partial response | 2 (12.5) | 0 |
| Stable disease | 10 (62.5) | 3 (37.5) |
| Progressive disease | 2 (12.5) | 0 |
| Not evaluable | 2 (12.5) | 5 (62.5) |
| Objective response rate (95%CI), % | 12.5(1.55, 38.35) | 0(0.00, 36.94) |
| P value | 0.4386 | |
| Disease control rate (95% CI), % | 75(47.62, 92.73) | 37.5(47.62, 92.73) |
| P value | 0.1445 | |
| mPFS  (95% CI),month | 5.5（1.8，7.4） | 1.6 (0.8,2.9) |
| HR(95%CI) | 0.41 (0.12,1.37) | |
| mOS  (95% CI),month | 7.0 (3.1,11.3) | 3.0 (0.8,5.8) |
| HR(95%CI) | 0.39 (0.13,1.17) | |

**Supplementary Table 10. Comparison of safety between two groups with Child-Pugh score of 7**

|  | Finotonlimab plus bevacizumab biosimilar (n=16) n (%) | Sorafenib  (n=8)  n (%) |
| --- | --- | --- |
| Treatment-emergent adverse events (TEAE) | 16(100) | 7 (87.5) |
| TEAE related to either drug (TRAE) | 15 (93.8) | 7 (87.5) |
| Treatment-emergent serious adverse events (TESAE) | 5 (31.3) | 2 (25.0) |
| TESAE related to either drug (TRSAE) | 2 (12.5) | 2 (25.0) |
| TEAE caused death | 3 (18.8) | 0 |
| TRAE caused death | 2 (12.5) | 0 |
| TEAE leading to interrupt to either drug | 10 (62.5) | 3 (37.5) |
| TRAE leading to interrupt to either drug | 7 (43.8) | 3 (37.5) |
| TEAE leading to discontinue to either drug | 3 (18.8) | 0 |
| TRAE leading to discontinue to either drug | 2 (12.5) | 0 |
