# Supplementary material for: Finotonlimab (PD-1 inhibitor) plus bevacizumab (bevacizumab biosimilar) as first-tier therapy for late-stage hepatocellular carcinoma: a randomized phase 2/3 trial
Source: Signal Transduct Target Ther. 2025 Aug 6;10:249. doi: 10.1038/s41392-025-02333-5 (PMC12329032; doi:10.1038/s41392-025-02333-5)
Supplement: Supplementary file 2 — Protocol of the clinical trial [file 41392_2025_2333_MOESM2_ESM.pdf]

SCT-I10A in Combination with SCT510 Versus Sorafenib as First-Line Therapy for Advanced Hepatocellular Carcinoma(HCC)
a Multicenter, Randomized, Open-label Phase II/III Trial

Protocol number	SCT-I10A-C301
Investigational drug	Recombinant humanized anti-PD-1 monoclonal antibody injection (SCT-I10A)<br>Recombinant humanized anti-VEGF monoclonal antibody injection (SCT510)
Sponsor	Sinocelltech Ltd.
Version number	V2.0
Version date	2021-04-20

Confidentiality statement

All information contained in this protocol is owned by Sinocelltech Ltd. and is provided solely for review by researchers, collaborating researchers, ethics committees, and relevant regulatory authorities. Without the written approval of Sinocelltech Ltd., it is strictly prohibited to disclose any information to third parties unrelated to this study, except for necessary explanations provided to patients who may participate in this study when signing the informed consent form.

Table of Contents

List of Abbreviations 6
1 Research background 32
1.1 Overview of the epidemiology and treatment status of hepatocellular carcinoma 32
1.1.1 Epidemiological characteristics 32
1.1.2 Current status of first-line treatment for hepatocellular carcinoma 32
1.1.3 Immunotherapy for advanced hepatocellular carcinoma 33
1.1.4 Immune checkpoint inhibitors combined with anti VEGF targeted therapy 35
1.2 SCT-I10A 37
1.2.1 SCT-I10A Overview 37
1.2.2 Experience from preclinical pharmacodynamics studies of SCT-I10A 38
1.2.3 Experience in preclinical pharmacokinetics studies of SCT-I10A 38
1.2.4 SCT-I10A Preclinical toxicology study 39
1.2.5 Phase I clinical study data of SCT-I10A 41
1.3 SCT510 44
1.3.1 Overview of SCT510 44
1.3.2 Pharmaceutical comparative study 44
1.3.3 Preclinical efficacy comparison study 45
1.3.4 Preclinical pharmacokinetics comparative study 45
1.3.5 Preclinical toxicology comparative study 46
1.3.6 SCT510 I Phase clinical study data 47
2 Study Objectives 48
2.1 Phase II single-arm study 48
2.2 Phase III randomized controlled trial 48
3 Study design 50
3.1 Principles of study design 50
3.2 Study design 50
3.2.1 Overall study design 50
3.3 Risk / Benefit assessment 54
3.4 Evaluation Criteria Indicators 56
3.4.1 Phase II single-arm study 56
3.4.2 Phase III randomized controlled trial 57
3.5 Randomization and Blinding 58
3.5.1 Randomization Method 58
3.5.2 Blinding 58
3.6 End of Study (EOS) 58
3.7 Trial termination 59
3.8 Closure of the study and research centers 60
4 Study Population 61
4.1 Inclusion Criteria 61
4.2 Exclusion criteria 62
4.3 Early withdrawal from study / Treatment 65

4.3.1 Subject withdrawal from treatment 65
4.3.2 Patients may withdraw from the study on their own accord. 68
5 Investigational drug 69
5.1 Basic information on the investigational drug 69
5.1.1 SCT-I10A 69
5.1.2 SCT510 69
5.2 Drug preparation and administration 70
5.2.1 SCT-I10A 70
5.2.2 SCT510 70
5.2.3 Sorafenib 71
5.3 Adjustments to investigational drug treatment 71
5.3.1 SCT-I10A Treatment adjustments 71
5.3.2 SCT510 Treatment adjustment 76
5.3.3 Adjustment of Sorafenib Treatment 81
5.3.4 Other medications 84
5.4 Termination of study treatment after CR 84
5.5 Combination therapy / Treatment 84
5.6 Prohibited drugs / Treatment 85
5.7 Management of investigational drugs 86
5.7.1 Transport / Receive / Store / Safeguard 86
5.7.2 Distribution / Use / Return / Destruction 86
5.7.3 Records 87
5.8 Other safety considerations and risk management 87
6 Study procedures and evaluation criteria 88
6.1 Study procedures 88
6.1.1 Subject Screening 88
6.1.2 Study treatment period 92
6.1.3 End of Treatment Visit 95
6.1.4 Survival follow-up 96
6.2 Efficacy assessment indicators 96
6.2.1 Tumor assessment time 97
6.2.2 Tumor assessment requirements 97
6.2.3 Blinded independent central imaging review 98
6.2.4 Quality of life assessment 98
6.3 Safety assessment 99
6.3.1 Weight and height 99
6.3.2 Vital signs 99
6.3.3 Physical examination 99
6.3.4 ECOG Score 100
6.3.5 12-ECG 100
6.3.6 Cardiac echocardiography 100
6.3.7 Laboratory tests 100

6.3.8 Adverse events 102
6.3.9 Concomitant medication and treatment 102
6.4 Biomarkers 102
6.5 Pharmacokinetic sample collection and analysis 102
6.6 Immunogenicity 103
7 Adverse events and severe adverse events 104
7.1 Adverse events 104
7.1.1 Timeframe for adverse event collection 104
7.1.2 Follow-up of adverse events 104
7.1.3 Description of adverse events 105
7.1.4 Analysis of the association between adverse events and the investigational drug 106
7.1.5 Handling of abnormal laboratory test results 107
7.1.6 Severity of adverse events 108
7.1.7 Outcome of adverse events 109
7.2 Severe adverse events 109
7.2.1 Definition 109
7.2.2 Reporting of severe adverse events 109
7.2.3 Follow-up of severe adverse events 110
7.3 Suspected unexpected serious adverse reactions 110
7.3.1 Definition 110
7.3.2 Report of suspected unexpected serious adverse reactions 110
7.3.3 Follow-up of unexpected serious adverse reactions 111
7.4 Management of pregnancy events 112
7.5 Special attention to adverse events 112
8 Data management 114
8.1 Data Management Plan 114
8.2 CRF and database 114
8.3 Data entry and verification 114
8.4 Data Verification and Query Management 115
8.5 External data and medical coding 115
8.6 Database lock 115
9 Statistical analysis 116
9.1 Sample size 116
9.2 Analysis Population 117
9.3 Statistical analysis methods 118
9.3.1 General principles 118
9.3.2 Case distribution 118
9.3.3 Baseline and demographic characteristics 118
9.3.4 Compliance and drug exposure analysis 118
9.3.5 Efficacy analysis 119
9.3.6 Safety analysis 122

9.3.7 Pharmacokinetic analysis 123
9.3.8 Immunogenicity analysis 123
9.3.9 Combination therapy 123
9.4 Mid-term analysis 123
9.5 Multiplicity considerations 123
9.6 Other analyses 124
9.7 Independent Data Monitoring Committee (IDMC) 124
10 Ethics 125
10.1 Ethics Committee 125
10.2 Informed consent of patients 125
10.3 Confidentiality of patient information 126
11 Management of the study 127
11.1 Sponsor 127
11.2 Investigator 127
11.3 Clinical monitoring 127
11.4 Medical Monitoring 129
11.5 Audit 129
11.6 Recording and preservation of research data 130
12 Registration and publication of research results 131
13 References 132

List of Abbreviations

ADA	Drug-resistant antibodies
ADCC	Antibody-dependent cell-mediated cytotoxicity
AE	Adverse events
AESI	Adverse events of special concern
AFP	Alpha-fetoprotein
AJCC	American Joint Committee on Cancer
ALB	Albumin
ALP	Alkaline phosphatase
ALT	Alanine aminotransferase (ALT)
APTT	Activated partial thromboplastin time
AST	Aspartate aminotransferase (AST)
ATC	Anatomical-Therapeutic-Chemical Classification System
AUC	Area under the concentration-time curve
BCG	BCG (Bacillus Calmette-Guérin)
BCLC	Barcelona Clinic Liver Cancer staging
BID	Twice daily
BICR	Blinded independent central review
CDC	Complement-dependent cytotoxicity
CRF	Case report form
CHO	Chinese hamster ovary cells
CI	Confidence interval
CL	Clearance rate
C_{max}	Peak concentration
C_{min}	Trough concentration
CNS	Central nervous system
CPIs	Immune checkpoint inhibitors
CR	Complete response
CRO	Contract research organization
Cr	Creatinine
Ccr	Creatinine clearance rate
CT	Computed Tomography
CTCAE	Common Terminology Criteria for Adverse Events
DCR	Disease Control Rate

DOR	Duration of Response
ECOG	Eastern Cooperative Oncology Group Performance Status
EDC	Electronic Data Capture System
12-ECG	12-lead Electrocardiogram
EOT	End of Treatment Visit
EOS	End of Study
EPO	Erythropoietin
ESMO	European Society for Medical Oncology
FAS	Full analysis set
FDA	U.S. Food and Drug Administration
FT3	Free T3
FT4	Free T4
μg	Microgram
GCP	Good Clinical Practice for Clinical Trials
G-CSF	Granulocyte colony-stimulating factor
GM-CSF	Granulocyte-macrophage colony-stimulating factor
GLP	Good Laboratory Practice for Nonclinical Studies
GMR	Geometric mean ratio
HbsAg	Hepatitis B surface antigen
HBV	Hepatitis B virus
HCC	Hepatocellular carcinoma
HCV	Hepatitis C virus
HIV	Human immunodeficiency virus
β-hCG	Human chorionic gonadotropin
ICH	International Conference on Technical Requirements for Registration of Pharmaceuticals for Human Use
IDMC	Independent Data Monitoring Committee
INR	International Standardized Ratio
IRB	Institutional Review Board
Kg	Kilogram
LVEF	Left Ventricular Ejection Fraction
mAb	Monoclonal Antibody
MedDRA	International Dictionary of Medical Terms
mg	Milligram
mL	Milliliter

mmHg	Millimeters of Mercury
MMP	Medical Monitoring Plan
MRI	Magnetic Resonance Imaging
mRECIST	Modified Response Evaluation Criteria in Solid Tumors
MTD	Maximum Tolerated Dose
nM	Nanomole
NE	Unable to Evaluate
NMPA	National Medical Products Administration
NSAID	Non-steroidal anti-inflammatory drugs
NYHA	New York Heart Association
ORR	Objective response rate
OS	Overall survival
OSR	Overall survival rate
PD	Disease progression
PFS	Progression-free survival
PK	Pharmacokinetics
PPS	Protocol compliance
PR	Partial response
PRES	Reversible posterior leukoencephalopathy syndrome
PT	Prothrombin time
QA	Quality assurance
Q3W	Every three weeks
RECIST	Response Evaluation Criteria in Solid Tumors
SAE	Severe adverse events
SAP	Statistical analysis plan
SD	Disease stability
SOC	System organ classification
SS	Safety set
TBIL	Total bilirubin
TEAE	Adverse events during treatment
TSH	Thyroid-stimulating hormone
$t_{1/2}$	Terminal elimination half-life
Vz	Apparent volume of distribution
VEGF	Vascular endothelial growth factor
VEGFR	Vascular endothelial growth factor receptor

WBC	Leukocytes

Protocol Synopsis

**Study Title**	SCT-I10A in Combination With SCT510 Versus Sorafenib as First-Line Therapy for Advanced Hepatocellular Carcinoma (HCC): A Multicenter, Randomized, Open-label, Phase 2/3 Trial
**Version Number/ Version Date**	Version Number: V2.0/Version Date: 2021-04-20
**Sponsor**	Sinocelltech Ltd.
**Research Centers**	Planned 40-70 research centers
**Indication**	Advanced hepatocellular carcinoma without prior systemic treatment
**Investigational Drugs**	Recombinant humanized anti-PD-1 monoclonal antibody injection (SCT-I10A), recombinant humanized anti-VEGF monoclonal antibody injection (SCT510)
**Study Objectives**	**Phase II Single-arm Study**<br>**Primary Study Objective**:<br>• To evaluate the safety and tolerability of SCT-I10A combined with SCT510 in patients.<br>**Secondary Study Objectives:**<br>• To evaluate the objective response rate (ORR), duration of response (DOR), disease control rate (DCR), progression-free survival (PFS), and overall survival (OS) of patients treated with SCT-I10A combined with SCT510 according to RECIST v1.1 criteria;<br>• To evaluate the pharmacokinetic characteristics of SCT-I10A combined with SCT510;<br>• To evaluate the immunogenicity of SCT-I10A and SCT510.<br>**Phase III Randomized Controlled Trial**<br>**Primary Study Objective**:<br>• Evaluation of progression-free survival (PFS) and overall survival (OS) based on blinded independent central review (BICR) according to RECIST v1.1 for patients treated with SCT-I10A combined with SCT510 compared to Sorafenib.<br>**Secondary Study Objectives:**<br>• One-year, 1.5-year, and 2-year overall survival rates (OSR) for patients treated with SCT-I10A combined with SCT510 compared to Sorafenib;<br>• Investigator assessment of progression-free survival (PFS) for patients treated with SCT-I10A combined with SCT510 compared to Sorafenib according to RECIST v1.1criteria;<br>• BICR and investigator assessment of objective response rate (ORR), duration of response (DOR), and disease control rate (DCR) for patients treated with SCT-

	I10A combined with SCT510 compared to Sorafenib according to RECIST v1.1 criteria;<br>• BICR evaluated the progression-free survival (PFS), objective response rate (ORR), duration of response (DOR), and disease control rate (DCR) of SCT-I10A combined with SCT510 compared to Sorafenib in patients according to mRECIST criteria;<br>• Evaluate the safety of SCT-I10A combined with SCT510 in patients;<br>• Evaluate the quality of life of patients treated with SCT-I10A combined with SCT510 compared to Sorafenib (EORTC QLQ-C30 and EORTC QLQ-HCC18 scales);<br>• Evaluate the steady-state trough concentrations and pharmacokinetic characteristics of SCT-I10A and SCT510 in patients with advanced hepatocellular carcinoma;<br>• Evaluate the immunogenicity of SCT-I10A and SCT510;<br>• Evaluate the correlation between PD-L1 expression levels and efficacy, prognosis.
**Study Design**	This study is a multicenter, randomized, open-label Phase II/III clinical study to evaluate the efficacy and safety of SCT-I10A combined with SCT510 compared to Sorafenib as first-line treatment for advanced hepatocellular carcinoma, divided into two parts: a Phase II single-arm study and a Phase III randomized controlled study. The Phase II single-arm study serves as a safety introduction phase, primarily evaluating the safety and tolerability of SCT-I10A combined with SCT510 treatment, with a planned enrollment of 50-60 patients, receiving SCT-I10A 200mg on day 1 every 3 weeks combined with SCT510 15mg/kg on day 1 every 3 weeks, until disease progression, intolerable toxicity, initiation of new antitumor therapy, decision to discontinue treatment after careful consideration by the patient or investigator, death, or loss to follow-up. After disease progression, if the investigator determines that continued treatment with SCT-I10A combined with SCT510 may provide clinical benefit, treatment with SCT-I10A combined with SCT510 may continue after obtaining patient consent, until the investigator determines that there is no longer clinical benefit, intolerable toxicity, the patient or investigator decides to terminate treatment after careful consideration, death, or loss to follow-up.<br><br>It is planned to analyze the safety and efficacy of the Phase II single-arm study part before the third administration of the last patient in the Phase II study (after completing the first efficacy evaluation), and after preliminary confirmation of safety and efficacy, to enter the Phase III randomized, controlled study.<br><br>The second part is a Phase III randomized, controlled study, planned to enroll approximately 342 patients, who will be randomly assigned to the trial group and control group in a 2:1 ratio.

	**Screening Period:**<br><br>After patients sign the informed consent form, they will enter the study screening period, during which screening evaluations will be completed according to the visit schedule in the study flowchart. The maximum duration of the screening period is 28 days, and patients who meet all inclusion criteria and do not meet any exclusion criteria will be eligible to enter the treatment period of the study.<br><br>**Treatment Period:**<br><br>Eligible patients will be randomly assigned (2:1) to the trial group and control group, with random stratification factors including ECOG (0 vs 1), baseline AFP levels (<400 ng/ml vs ≥400 ng/ml), and the presence or absence of major vascular invasion and/or extrahepatic metastasis (none vs present). The trial group received the SCT-I10A combined with SCT510 regimen, while the control group received the Sorafenib regimen.<br><br>**Trial Group:** SCT-I10A: 1 cycle every 3 weeks (21 days), administering 200mg on day 1 of each cycle via intravenous infusion, with a treatment duration not exceeding 2 years.<br><br>SCT510: 1 cycle every 3 weeks (21 days), administering 15mg/kg on day 1 of each cycle via intravenous infusion, with a treatment duration not exceeding 2 years.<br><br>Administration sequence: first infuse SCT-I10A, then administer SCT510.<br><br>**Control Group:** Sorafenib: 1 cycle every 3 weeks (21 days), with each treatment cycle lasting 400mg taken twice daily (preferably 12 hours apart) on an empty stomach (1 hour before meals or 2 hours after meals), with a treatment duration not exceeding 2 years.<br><br>The control group received Sorafenib treatment until disease progression, intolerable toxicity, the initiation of new antitumor therapy, termination of treatment decided after careful consideration by the patient or investigator, death, or loss to follow-up. After disease progression, if the investigator determines that continued treatment with Sorafenib may provide clinical benefit, it may continue with the patient's consent until the investigator determines that there is no clinical benefit, intolerable toxicity, termination of treatment decided after careful consideration by the patient or investigator, death, or loss to follow-up.<br><br>The trial group received SCT-I10A combined with SCT510 treatment until disease progression, intolerable toxicity, the initiation of new antitumor therapy, a decision to terminate treatment made after careful consideration by the patient or investigator, death, or loss to follow-up. After disease progression, if the investigator determines that continued treatment with SCT-I10A combined with SCT510 may provide clinical benefit, treatment with SCT-I10A combined with SCT510 may continue after obtaining patient consent, until the investigator determines that there is

	no longer clinical benefit, intolerable toxicity, the patient or investigator decides to terminate treatment after careful consideration, death, or loss to follow-up. Clinical benefit is the result of the investigator's comprehensive assessment based on the absence of intolerable toxicity or symptom deterioration due to disease progression, combined with imaging findings and clinical status.<br><br>During the screening period of this study and throughout the entire study, the investigator will determine whether the patients should continue treatment based on the Response Evaluation Criteria in Solid Tumors (RECIST) version 1.1. Baseline tumor assessment will be conducted within 28 days prior to the initiation of the experimental drug treatment. It is recommended that tumor assessments be performed using enhanced computed tomography (CT) or magnetic resonance imaging (MRI) (enhanced CT is preferred; if the patient is allergic to CT contrast agents, a CT chest scan without contrast plus MRI of other areas may be used). From the start of the first dose of study treatment until week 48, tumor efficacy evaluations will be conducted every 6 weeks (±7 days); after week 48, evaluations will occur every 9 weeks (±7 days). The timing of efficacy evaluation visits is fixed, regardless of whether there is a delay in the treatment cycle. Until there is no clinical benefit, a new antitumor treatment begins, informed consent is withdrawn, death occurs, or loss to follow-up, whichever occurs first. If symptoms indicative of PD occur, subsequent imaging methods need to be employed for assessment to record and confirm tumor efficacy.<br><br>From the start of the first dose of study treatment, safety assessments will be conducted every 2 cycles (±3 days). During the study treatment period, additional safety assessments are required: including physical examinations and vital signs before each administration of SCT-I10A and SCT510, before each cycle of Sorafenib treatment, ECOG performance status, 12-lead ECG, and basic laboratory tests (blood routine, urinalysis, blood biochemistry). If the safety assessment time coincides with the administration time on the same day, it must be completed before the first administration of the investigational drug.<br><br>**Follow-up period:**<br><br>End of treatment visit (EOT): Patients who continue treatment after disease progression without clinical benefit, experience intolerable toxicity, or require termination of investigational drug treatment for any other reason must undergo an end of treatment visit. All patients will be followed for adverse events. From the time the patients receive the investigational drug until 90 days after the last dose or before starting any new antitumor therapy, whichever occurs first, all adverse medical events will be collected. After this period, only serious adverse events related to the investigational drug or study procedures need to be collected. For adverse events (AEs), follow-up must continue until any of the following conditions are met: the event resolves, the event stabilizes, the event returns to baseline levels, or the investigator reasonably determines (for reasons such as inability to recover or improvement) that

	further follow-up is unnecessary, or more information cannot be obtained (such as loss to follow-up or refusal to provide additional information by the patient).<br><br>Survival follow-up visits: After the end-of-treatment visit, patients will undergo follow-up for survival status. After the end-of-treatment visit, patients who terminate study treatment for reasons other than disease progression, initiation of new antitumor therapy, death, or loss to follow-up should continue to receive tumor assessments during this period until disease progression, initiation of new antitumor therapy, withdrawal of informed consent, death, or loss to follow-up occurs, whichever comes first. All patients who have received at least one dose of the investigational drug will undergo survival status follow-up every 3 months (±14 days), which may be conducted via telephone inquiries, to collect information on new antitumor therapies and survival status starting from the end of treatment visit, until death, loss to follow-up, or withdrawal of informed consent.
**Number of Patients**	The Phase II study plans to enroll 50-60 patients, while the Phase III study plans to enroll 342 patients.
**Inclusion Criteria**	Patients who meet all of the following criteria may be included in this study:<br>1. Voluntarily signed a written informed consent form prior to screening;<br>2. Age ≥ 18 years, regardless of gender;<br>3. ECOG performance status score of 0-1;<br>4. Clinically or pathologically diagnosed with hepatocellular carcinoma, with the diagnosis of hepatocellular carcinoma meeting at least one of the following criteria:<br>1) Histologically or cytologically confirmed hepatocellular carcinoma, excluding fibrolamellar, sarcomatoid, or mixed cholangiocarcinoma-hepatocellular carcinoma;<br>2) Clinically diagnosed as HCC according to the 'Guidelines for Diagnosis and Treatment of Primary Liver Cancer (2019 Edition)';<br>5. Patients who have not previously received any systemic therapy for HCC (primarily including systemic chemotherapy, anti-angiogenic therapy, molecular targeted therapy, and any other immunotherapy involving PD-1, anti-PD-L1/L2, anti-CD137, or anti-CTLA-4 antibodies, or targeting T-cell co-stimulation or immune checkpoint pathways) are allowed to enroll if disease progression occurs 6 months after the completion of postoperative adjuvant chemotherapy;<br>6. Barcelona Clinic Liver Cancer (BCLC) staging is classified as stage C or B, which is not suitable for surgery and/or local treatment;<br>7. Child-Pugh liver function classification: Class A and better Class B (≤7 points), with no history of hepatic encephalopathy;<br>8. According to the Response Evaluation Criteria in Solid Tumors (RECIST version 1.1), at least one measurable lesion is required, and lesions that have shown clear disease progression after prior local treatment may also be selected as target

	lesions;<br>9. Expected survival period exceeds 3 months;<br>10. Major organ functions are normal (no blood components, cell growth factors, or albumin infusion correction treatments were administered within 14 days prior to laboratory tests), meeting the following criteria:<br>1) Blood routine: Neutrophils ≥1.5×10^9 /L, Platelets ≥75×10^9 /L, Hemoglobin ≥90g/L;<br>2) Liver function: Alanine aminotransferase (ALT) and Aspartate aminotransferase (AST), ALT and AST ≤5×ULN; Serum albumin ≥28g/L; Total bilirubin (TBIL) ≤2×ULN; Alkaline phosphatase (ALP) ≤5×ULN.<br>3) Kidney function: Serum creatinine (Cr) ≤1.5×ULN or Creatinine clearance rate (Ccr) ≥50mL/min;<br>4) Urinalysis detects urinary protein <2 (+); If the baseline urinary protein is ≥2 (+), the 24-hour urinary protein quantification must be ≤1.0g;<br>5) Coagulation function: Activated partial thromboplastin time (APTT), International normalized ratio (INR), Prothrombin time (PT) ≤1.5×ULN;<br>6) Cardiac echocardiogram: Left ventricular ejection fraction (LVEF) ≥50%;
**Exclusion Criteria**	Patients meeting any of the following criteria should be excluded from this study:<br>1. Within 4 weeks prior to the first investigational drug treatment, patients who have undergone liver surgery and/or local treatment for HCC, except for palliative radiotherapy to relieve pain for bone metastatic lesions;<br>2. Patients who have not recovered from any toxicity and/or complications from local treatments (including interventional treatments, radiofrequency treatments, etc.), prior chemotherapy, surgery, or radiotherapy, that have not decreased to ≤1 grade (NCI CTCAE version 5.0) (except for hair loss and fatigue);<br>3. Patients who received antitumor drug treatment (including but not limited to traditional Chinese medicine formulations with antitumor indications) within 2 weeks prior to the first investigational drug treatment;<br>4. Moderate or severe ascites or ascites requiring drainage within 4 weeks prior to the first investigational drug treatment, or pleural effusion or pericardial effusion requiring drainage and/or accompanied by symptoms of dyspnea;<br>5. Patients with known active central nervous system metastases (CNS) and/or carcinomatous meningitis: previously treated patients with brain metastases may participate in the study, provided they are clinically stable for at least 2 weeks, with no evidence of new or enlarged brain metastases, and steroids are discontinued 14 days prior to the administration of the investigational drug. Stable brain metastases in this definition should be determined prior to the first administration of the investigational drug. Asymptomatic patients with brain metastases (i.e., no neurological symptoms, no need for corticosteroids, and no lesions >1.5 cm) may participate, but regular brain imaging should be conducted as a disease site.

	6. Excluding patients with other malignant tumors (cured cervical carcinoma in situ, non-melanoma skin cancer, or other tumors/cancers that have undergone radical treatment and have shown no signs of disease for at least 5 years);<br>7. Patients with a tendency to bleed, high bleeding risk, or coagulation disorders, including those with a history of arterial or venous thromboembolic events occurring within 6 months prior to screening, such as myocardial infarction, unstable angina, cerebrovascular accidents or transient ischemic attacks, pulmonary embolism, deep vein thrombosis, or any other serious thromboembolic events (excluding those with implanted venous infusion ports or catheter-related thrombosis, or superficial venous thrombosis that stabilized after conventional anticoagulation treatment); Any life-threatening bleeding events occurring within the 3 months prior to screening, including those requiring blood transfusion, surgery, or local treatment, or ongoing drug treatment; Use of full-dose oral or parenteral anticoagulants, thrombolytics, or aspirin (>325mg/day) or other platelet function inhibitors (such as clopidogrel) within 10 days prior to enrollment; Or having previously undergone surgical procedures, with lesions invading major blood vessels, as determined by the investigator to have a bleeding tendency;<br>Note: Prophylactic anticoagulation for maintaining venous access is permitted, provided that INR ≤ 1.5×ULN and APTT is within the normal range within 14 days prior to enrollment;<br>8. Portal vein involvement of the main trunk and left and right branches, or simultaneous involvement of the main trunk and superior mesenteric vein, or presence of inferior vena cava thrombosis or cardiac involvement;<br>9. Presence of unhealed wounds, active gastrointestinal ulcers or bleeding, fractures (excluding healed old fractures);<br>10. History of esophageal or gastric variceal bleeding within 6 months prior to the first investigational drug treatment. Known severe varices on endoscopy within 3 months prior to enrollment. Evidence of portal hypertension, assessed by the investigator as having a high risk of bleeding;<br>11. History of gastrointestinal perforation and/or fistula, abdominal abscess, intestinal obstruction (including incomplete intestinal obstruction requiring parenteral nutrition), extensive bowel resection (partial colectomy or extensive small bowel resection with chronic diarrhea), Crohn's disease, ulcerative colitis, or long-term chronic diarrhea within 6 months prior to the first investigational drug treatment;<br>12. Patients who have undergone major surgery within 4 weeks prior to enrollment or are expected to undergo major surgery during the study period (excluding diagnostic biopsy);<br>13. Active, known, or suspected autoimmune diseases (such as systemic lupus erythematosus, rheumatoid arthritis, inflammatory bowel disease, autoimmune

thyroid disease, multiple sclerosis, vasculitis, glomerulonephritis, etc.), a history of human immunodeficiency virus infection (HIV positive), or other acquired or congenital immunodeficiency diseases. However, the following patients are allowed to be enrolled: patients with type 1 diabetes whose condition is stable after receiving a fixed dose of insulin; Patients with autoimmune hypothyroidism receiving stable doses of hormone replacement therapy; Skin diseases that do not require systemic treatment (such as eczema with rashes covering less than 10% of the body surface area, psoriasis without ophthalmic symptoms, etc.);

14. History of liver or other organ transplantation or stem cell transplantation;
15. Known history of active tuberculosis;
16. Known history of interstitial lung disease, non-infectious pneumonia, or patients highly suspected of having interstitial lung disease; Patients with a history of drug-induced or radiation-induced non-infectious pneumonia who are asymptomatic are allowed to enroll;
17. Active infections requiring systemic therapy within 14 days prior to the first investigational drug treatment, or uncontrolled infections (excluding simple urinary tract infections or upper respiratory infections), and patients requiring treatment for syphilis;
18. Patients with severe medical conditions, such as grade III or higher cardiac dysfunction (New York Heart Association [NYHA]), ischemic heart disease (such as myocardial infarction or angina), or a history of myocardial infarction within the last 3 months that remains poorly controlled after medication, diabetes (fasting blood glucose ≥10mmol/L) that remains poorly controlled after medication, or hypertension (systolic blood pressure >140mmHg and/or diastolic blood pressure >90mmHg) that remains poorly controlled after medication, as well as patients with a history of hypertensive crisis or hypertensive encephalopathy;
19. Patients with acute or chronic active hepatitis B or C virus infection, with hepatitis B virus (HBV) DNA > 2000 IU/ml or 10^4 copies/ml; Hepatitis C virus (HCV) RNA > 10^3 copies/ml; Hepatitis B surface antigen (HbsAg) and anti-HCV antibodies both positive; Patients who have undergone antiviral treatment and meet the above criteria and are willing to continue antiviral treatment during the study can be enrolled;
20. Exclusion criteria include the use of immunosuppressive drugs within 2 weeks prior to enrollment or during the study period:
    1) Intranasal, inhaled, topical steroids, or local steroid injections (e.g., intra-articular injections);
    2) Physiological doses of systemic corticosteroids (≤10mg/day of prednisone or equivalent dose);
    3) Short-term (≤7 days) use of steroids for the prevention or treatment of non-autoimmune allergic diseases;
21. Administration of live vaccines within 4 weeks prior to the first dose of

	investigational drugs or planned during the study (excluding live virus seasonal influenza vaccines);<br>22. Allergy to any component of the investigational drugs SCT-I10A, SCT510; or known allergy to any other monoclonal antibody; or allergy to any component of Sorafenib;<br>23. Pregnant or breastfeeding women, or patients planning to become pregnant during the treatment period and within 6 months after treatment ends;<br>24. Patients of childbearing potential who are unwilling to use effective contraceptive measures (including male patients capable of causing pregnancy and female patients and their male partners) during the study period and for at least 6 months after the last administration of the investigational drug;<br>25. Patients currently enrolled in other investigational devices or investigational drug treatments, with a time since the last use of other investigational drugs or devices of less than or equal to 4 weeks;<br>26. It is known that the patients have a history of drug addiction or mental illness;<br>27. Any other diseases, metabolic disorders, or laboratory test abnormalities that the investigator deems make the patient unsuitable for investigational drug treatment, or that may affect the interpretation of the study results, or place the patient at high risk, or affect the collection of data and samples.
**Study treatment**	**Phase II and III experimental groups:** SCT-I10A: 200 mg administered via intravenous infusion on Day 1 of each cycle (every 3 weeks, 21 days), with a treatment duration not exceeding 2 years.<br><br>SCT-510: 15 mg/kg will be administered via intravenous infusion on Day 1 of each cycle, every 3 weeks (21 days), for a duration not exceeding 2 years.<br><br>Administration sequence: SCT-I10A will be infused first, followed by SCT510, with an interval of more than 30 minutes between the end of SCT-I10A infusion and the start of SCT510 infusion.<br><br>If any drug is paused or permanently discontinued, other drugs may still be continued for treatment. If any drug is delayed or interrupted due to adverse events during a treatment cycle, other investigational drugs that have not caused intolerable toxicity may continue to be administered during that cycle. If the investigational drug is delayed by more than 7 days, the current cycle will be paused, and administration will resume in the next cycle. If the investigational drug is delayed by 7 days or less, the drug can continue to be used in the current cycle. For example, if SCT510 is delayed due to an adverse event, SCT-I10A will continue to be administered as planned. If SCT510 is delayed by 7 days or less, treatment with SCT510 can continue in the current cycle. If SCT510 is delayed by more than 7 days, administration of SCT510 will be paused in the current cycle and will resume in the next cycle. The delay time is calculated based on the first administration of the other investigational drug in the current cycle.

	**Phase III control group:** Sorafenib: 3 weeks (21 days) constitutes one cycle, with each treatment cycle lasting for daily 400mg taken twice in the morning and evening on an empty stomach (with an interval of approximately 12 hours), and the medication duration should not exceed 2 years.<br><br>The control group received Sorafenib treatment until disease progression, intolerable toxicity, the initiation of new antitumor therapy, termination of treatment decided after careful consideration by the patient or investigator, death, or loss to follow-up. After disease progression, if the investigator determines that continued treatment with Sorafenib may provide clinical benefit, it may continue with the patient's consent until the investigator determines that there is no clinical benefit, intolerable toxicity, termination of treatment decided after careful consideration by the patient or investigator, death, or loss to follow-up.<br><br>The trial group received SCT-I10A combined with SCT510 treatment until disease progression, intolerable toxicity, the initiation of new antitumor therapy, a decision to terminate treatment made after careful consideration by the patient or investigator, death, or loss to follow-up. After disease progression, if the investigator determines that continued treatment with SCT-I10A combined with SCT510 may provide clinical benefit, treatment with SCT-I10A combined with SCT510 may continue after obtaining patient consent, until the investigator determines that there is no longer clinical benefit, intolerable toxicity, the patient or investigator decides to terminate treatment after careful consideration, death, or loss to follow-up. Clinical benefit is the result of the investigator's comprehensive assessment based on the absence of intolerable toxicity or symptom deterioration due to disease progression, combined with imaging findings and clinical status.
**Combination medication/Adjuvant therapy**	All medications administered from the signing of the informed consent form until 90 days after the last dose or before the initiation of new antitumor therapy (whichever occurs first) will be recorded in the EDC. Changes in medication dosage or adjustments will also be documented.<br><br>Investigators may provide standard treatment and palliative care for existing comorbidities, medical/surgical complications: administering sedatives, antiemetics, antibiotics, analgesics, antihistamines, steroids, granulocyte colony-stimulating factors, as well as red blood cells, erythropoietin, platelets, or fresh frozen plasma products to help manage pain, infections, and other complications of malignancies. If febrile neutropenia or evidence of infection occurs, intravenous antibiotic treatment may be administered.<br><br>For any immune-related adverse events (AEs) that arise, active treatment should be undertaken, and consultation with relevant specialty physicians should be sought if necessary. For the management of immune-related toxicities, please refer to the 2019 CSCO guidelines on the management of toxicities related to immune checkpoint inhibitors.

	During the trial treatment period, local radiotherapy may be implemented for pain relief or for osteolytic lesions at risk of fracture, but the patient's disease status should first be confirmed as not having progressed (PD) according to RECIST version 1.1. If a patient undergoes palliative radiotherapy after the initiation of study treatment, the reason for the treatment must be clearly documented, and target and non-target lesions must be assessed according to RECIST version 1.1. If radiation has been received during the trial, it cannot be further used for response assessment.<br><br>If a patient has already started bisphosphonate treatment prior to the initiation of study treatment, the use of bisphosphonates is permitted, regardless of the indication. If an increase in the ongoing bisphosphonate treatment dose or the initiation of bisphosphonate treatment is considered due to worsening bone pain, the patient's disease status must first be confirmed as PD according to RECIST version 1.1, unless disease progression is ruled out. Furthermore, it must be clearly documented in the patient's source documents; otherwise, patients who require the initiation of bisphosphonate treatment during the study will be assessed as having disease progression.<br><br>Systemic corticosteroids required to control infusion reactions or immune-related adverse events must be gradually tapered over at least one month and must not be used at immunosuppressive doses (equivalent to ≤10mg/day of prednisone) prior to the next administration of the investigational drug. For patients with a known allergy to diagnostic imaging contrast agents, the administration of steroids as a preventive treatment is permitted.<br><br>Patients may continue hormone replacement therapy if it was being used prior to enrollment.<br><br>Investigators may decide to administer any medication that is necessary for the health of the patients and will not interfere with the investigational drug.
**Prohibited drugs/treatments**	During the study treatment, patients must not receive additional systemic immunotherapy, chemotherapy, radiotherapy, biological therapy (including cytokines), immunosuppressants, or any other investigational drugs.<br><br>During the study treatment, no traditional Chinese medicine that is approved for antitumor therapy (those labeled with anticancer or antitumor in the product insert) is allowed. If necessary, the investigator may decide to administer traditional Chinese medicine for non-antitumor treatment indications, such as supportive care.<br><br>From 28 days prior to the first administration of the study treatment until the end of treatment, patients must not receive live vaccines, including but not limited to: measles, mumps, rubella, varicella, yellow fever, seasonal influenza, H1N1 influenza, rabies, BCG, and typhoid. Inactivated influenza vaccines may be used.<br><br>Patients receiving Sorafenib treatment should avoid the use of CYP3A4 inducers during the study treatment period: including but not limited to rifampicin; St. John's

	wort (Hypericum perforatum); phenytoin; carbamazepine; phenobarbital; and dexamethasone.
**Evaluation criteria**	**Phase II Single-arm Study**<br>**Primary study endpoint**:<br>1) Safety assessment endpoint:<br>• TEAE；<br>• Clinical laboratory test values;<br>• ECOG performance status score;<br>• Vital signs;<br>• 12-ECG；<br>• Thyroid function;<br>• Physical examination, etc.;<br>**Secondary study endpoints:**<br>1) Efficacy assessment endpoint: The investigator will evaluate the objective response rate (ORR), duration of response (DOR), disease control rate (DCR), progression-free survival (PFS), and overall survival (OS) of patients treated with SCT-I10A combined with SCT510 according to RECIST v1.1 criteria;<br>2） PK evaluation: Pharmacokinetic evaluation indicators include but are not limited to: AUC 0-t, AUC 0- ∞, C max, C min, CL, Vz, t 1/2, etc.;<br>3) Immunogenicity assessment: Detection of anti SCT-I10A antibodies and anti-SCT510 antibodies in the serum of patients before and after treatment in this study to evaluate immunogenicity.<br>**Phase III Randomized Controlled Trial**<br>**Primary study endpoint**:<br>• Evaluation of progression-free survival (PFS) and overall survival (OS) based on blinded independent central review (BICR) according to RECIST v1.1 for patients treated with SCT-I10A combined with SCT510 compared to Sorafenib.<br>**Secondary study endpoints:**<br>1) Other efficacy assessment endpoints:<br>• 1-year, 1.5-year, and 2-year survival rates (OSR);<br>• Investigators evaluate the progression-free survival (PFS) of patients treated with SCT-I10A combined with SCT510 compared to Sorafenib according to RECIST 1.1 criteria;<br>• BICR and investigators evaluated the objective response rate (ORR), duration of response (DOR), and disease control rate (DCR) of SCT-I10A combined with SCT510 compared to Sorafenib treatment in patients according to RECIST 1.1 criteria;

	• BICR evaluated the progression-free survival (PFS), objective response rate (ORR), duration of response (DOR), and disease control rate (DCR) of SCT-I10A combined with SCT510 compared to Sorafenib treatment in patients according to mRECIST criteria;<br>• Evaluate the changes in quality of life of patients in the experimental group and control group (EORTC QLQ-C30 and EORTC QLQ-HCC18 scales);<br>• Evaluate the correlation between PD-L1 expression levels and efficacy, prognosis;<br><br>2) Safety assessment endpoints:<br><br>• TEAE；<br>• Clinical laboratory test values;<br>• ECOG performance status score;<br>• Vital signs;<br>• 12-ECG；<br>• Thyroid function;<br>• Physical examination, etc.;<br><br>3) PK evaluation: Assess the steady-state trough concentrations and pharmacokinetic characteristics of SCT-I10A and SCT510 in patients with advanced hepatocellular carcinoma;<br>4) Immunogenicity assessment: Detect anti-SCT-I10A antibodies and anti-SCT510 antibodies in the serum of patients before and after treatment in this study to evaluate immunogenicity.
**Statistical methods**	**Analysis set**<br><br>Full analysis set (full analysis set, FAS): According to the intention-to-treat (ITT) principle, it includes all patients who were randomized and enrolled (Phase II includes all who signed the informed consent form and were successfully screened) and who received at least one dose of the study medication. The full analysis set is the primary analysis set for this study. In the analysis based on the full analysis set, patients will be assigned to the groups according to the randomization (regardless of the actual treatment they received) for participation in the analysis.<br><br>Per-protocol set (PPS): a subset of the full analysis set consisting of patients who adhered more closely to the protocol. Patients included generally require the following characteristics: completion of the minimum exposure to the designated experimental drug; availability of data for the primary endpoint indicators in the trial; no significant violations of the trial protocol. The analysis based on the per-protocol set will serve as a supportive analysis for this study.<br><br>Safety analysis set (safety set, SS): includes all patients who received at least one dose of the investigational drug and have at least one safety assessment after treatment,

	grouped according to the actual investigational drug received. The safety analysis set is used for the safety data analysis in this study.<br><br>Pharmacokinetics analysis set (PK analysis set, PKS): includes all patients who received at least one dose of the investigational drug and have at least one evaluation of drug concentration after treatment.<br><br>Baseline uses FAS, efficacy analysis uses both FAS and PPS, drug exposure/adherence and safety analysis use SS, and pharmacokinetics analysis uses PKS.<br><br>**Efficacy analysis**<br><br>Phase II is a single-arm study, with efficacy indicators as secondary endpoints, primarily evaluated using descriptive statistics. For Phase III studies, the Kaplan-Meier method is used to describe progression-free survival (PFS) and overall survival (OS), and to calculate the quartiles (25%, 50%, 75%) of event occurrence times along with their 95% confidence intervals; The log-rank test and Cox model are used for significance testing of risk ratios, calculating the objective response rate (ORR), disease control rate (DCR), duration of response (DOR), and 1-year, 1.5-year, and 2-year survival rates (OSR).<br><br>Descriptive statistics were used to summarize the scores of the EORTC QLQ-C30 and EORTC QLQ-HCC18 assessment scales at each planned evaluation time point and their changes from baseline.<br><br>**Safety analysis**<br><br>The analysis of safety endpoints was conducted in the safety analysis set, with separate analyses for Phase II and Phase III studies, including but not limited to the following aspects.<br><br>**Adverse events**<br><br>Adverse events were coded using the latest Chinese version of the Medical Dictionary for Regulatory Activities (MedDRA), and various adverse events were summarized according to the System Organ Class (SOC) and Preferred Term (PT).<br><br>Calculate the frequency, number of cases, and incidence of TEAE, grade 3 and above TEAE, drug-related TEAE, special attention adverse events, serious adverse events, and TEAE leading to treatment discontinuation. Summarize the severity of various TEAEs. The list describes the details of adverse events for each patient, including the type, severity, occurrence and duration, outcome, and relationship with the experimental drug and dosage.<br><br>**Laboratory tests**<br><br>A descriptive summary of laboratory test indicators at visit points will be provided, and the changes in indicators before and after treatment for patients will be

	summarized in a cross-table (based on clinical significance). A list of abnormal data after treatment for patients will be provided.<br>**Vital signs**<br>Describe the results of vital signs after treatment and their changes relative to baseline, and provide a list of those with abnormalities after treatment.<br>**ECG**<br>Summarize the number and percentage of patients with abnormal ECG findings, and present the changes in normal and abnormal indicators before and after treatment in a cross-tabulation format (based on clinical significance). List the data of patients who were normal before treatment and abnormal after treatment, as well as those who were abnormal before and after treatment. This includes the ECG assessment list for patients with at least one abnormal finding.<br>**Pharmacokinetic Analysis**<br>Descriptive statistics of pharmacokinetic parameters, including but not limited to**:** $AUC_{0\text{-}t}$, $AUC_{0\text{-}\infty}$, C_{max}, C_{min}, CL, Vz, $t_{1/2}$, etc.<br>**Immunogenicity analysis**<br>Descriptive statistics of anti-SCT-I10A antibodies and anti-SCT510 antibodies in the serum of patients to assess immunogenicity.
**Sample size estimation, efficacy hypothesis, and interim analysis**	**Sample size estimation:**<br>Phase III randomized open-label study:<br>Referencing data from the IMbrave150 study in the Chinese population [1], the mPFS for the Atezolizumab combined with Bevacizumab group compared to the Sorafenib group was 5.7 vs 3.2 months (HR=0.6), with the mOS for the experimental group not yet reached, while the control group (Sorafenib) was 11.4 months, HR=0.44. Also referencing data from the REFLECT study in the Chinese population, the PFS for the Sorafenib group was 3.6 months, OS was 10.2 months, and previous related studies indicated that Sorafenib had a PFS of approximately 3.5 months and an OS of 10-11.5 months in the Chinese population.<br>This study is designed as a superiority parallel design, with the primary objective of evaluating the efficacy of the experimental group (SCT-I10A combined with SCT510) compared to the control group (Sorafenib) in the target patients. The primary endpoints are set as a dual endpoint consisting of progression-free survival (PFS) and overall survival (OS) assessed by BICR. According to previous literature, the median PFS of Sorafenib in the Chinese population is approximately 3.5 months, and the median OS is 10-11.5 months. It is assumed that the median PFS of the control group in this study is 3.6 months, while the median PFS of the experimental group is 5.4 months, with a hazard ratio (HR) of 0.67. The median OS of the control group is assumed to be 12 months, while the median OS of the experimental group is 17.9

	months, also with an HR of 0.67. The two-sided significance level is set at 0.05, and the analysis of PFS and OS will be conducted using a fixed-sequential procedure. It is assumed that the enrollment period is 12 months, and after the first patient is randomly enrolled, approximately 16 months will be used for the two-sided significance level of 0.05 for the hypothesis testing of PFS. When 342 patients (with a 2:1 enrollment ratio for the experimental and control groups) are enrolled and 252 PFS events are reached, there will be at least an 85% probability of detecting the superiority hypothesis of the experimental group relative to the control group. The trial will end when the null hypothesis for PFS cannot be rejected. When the PFS rejects the null hypothesis, a hypothesis test for OS will be conducted approximately 36 months after random enrollment using a two-sided significance level of 0.05, thereby having an 85% confidence level to detect the efficacy hypothesis of the experimental group relative to the control group regarding OS. It is planned that at the final analysis of PFS, it is expected to observe 50% of the required OS events for an interim analysis of the OS endpoint, and using the approximate Lan-DeMets α spending function with the O'Brien Fleming boundary values for the judgment of interim analysis efficacy, that is, using a two-sided significance level of 0.0028 for the judgment of interim analysis efficacy, with the final analysis of OS having a two-sided significance level of 0.049.<br><br>Adjustments to the expected number of enrolled patients and events may be further detailed in the statistical analysis plan, considering the delayed effects of subsequent crossover treatments and immunotherapy.<br><br>**Efficacy hypothesis testing:**<br><br>For the PFS and OS endpoints, the null hypothesis H0: HR≥1; alternative hypothesis H1: HR<1<br><br>The overall Type I error rate is controlled at a two-sided significance level of 0.05. First, a two-sided significance level of 0.05 will be used to test PFS; if PFS does not reject the null hypothesis, the trial will end; If PFS rejects the null hypothesis, continue testing OS at a two-sided significance level of 0.05, conduct an interim analysis of OS at a two-sided significance level of 0.0028, and perform the final analysis of OS at a two-sided significance level of 0.049. If either of the efficacy endpoints for PFS or OS is statistically significant, then statistical superiority is established.<br><br>**Interim analysis:**<br><br>The Phase III portion of this study conducts two tests for OS, namely the interim analysis and the final analysis. An interim analysis of OS will be conducted when 50% of the required OS events (approximately 124 cases) are expected to be observed at the final analysis of PFS. And using the approximate Lan-DeMets α spending function with the O'Brien Fleming boundary values for the judgment of interim analysis efficacy, the two-sided 0.0028 significance level for the interim analysis and the two-sided significance level of 0.049 for the final analysis.

Table1 Study flow chart

**Study phase**	**Screening period**	**Treatment period**		**End of treatment visit**	**Survival status follow-up** [1]
Study cycle	NA	Cycle 1	Cycle 2-N	Within 7 days after the last administration, or within 7 days after learning/confirming early withdrawal	Every 3 months<br>Once
Study days	-28~-1	1	1	NA	NA
Time window (days)	-	NA	±3	NA	±14
Informed consent form [2]	X				
Inclusion/exclusion criteria confirmation	X				
Demographics	X				
Tumor diagnosis	X				
Previous antitumor therapy	X				
Medical history, surgical history, treatment history [3]	X				
Physical examination	X	X [4]	X	X	
Vital signs [5]	X	X	X	X	
Height	X				
Weight [6]	X	X	X	X	
12-ECG	X	X [4]	X	X	
ECOG score	X	X [4]	X	X	
Blood routine, urinalysis, blood biochemistry [7]	X	X [8]	X	X	
Coagulation function	X	X [8]	X [9]	X	
Thyroid function	X	X [8]	X [9]	X	
Cardiac ultrasound echocardiogram [10]	X				
Pregnancy test [11]	X	X [4]	X	X	
HBV, HCV, HIV tests [12]	X				

Study phase	Screening period	Treatment period		End of treatment visit	Survival status follow-up [1]
Study cycle	NA	Cycle 1	Cycle 2-N	Within 7 days after the last administration, or within 7 days after learning/confirming early withdrawal	Every 3 months Once
Study days	-28~-1	1	1	NA	NA
Time window (days)	-	NA	±3	NA	±14
HBV-DNA/HCV-RNA [13]	X		X	X	
AFP [14]	X	X	X	X	
Tumor assessment [15]	X		X	X	X [16]
Immunogenicity [17]		X	X	X	
Pharmacokinetics [18]		X	X	X	
Randomized [19]		X			
SCT-I10A [20]		X	X		
SCT510 [21]		X	X		
Sorafenib [22]		X	X		
Biological sample collection [23]	X				
Quality of life assessment [24]		X	X	X	
Concomitant medication	X	X	X	X	
Adverse events [25]		X	X	X	X
Subsequent antitumor therapy					X
Survival status					X

Note:

1) Survival status follow-up will be conducted every 3 months after the end of treatment visits (via telephone contact), until all patients withdraw informed consent, die, or are lost to follow-up;
2) Written informed consent must be obtained prior to the execution of any clinical research procedures (unless permitted by the protocol). This study allows patients who previously failed screening to be screened again; during re-screening, a new informed consent form must be signed and a new patient screening number must be obtained ;
3) Medical history and treatment history, including surgical history, within at least 1 year;
4) Acceptable screening period results within 7 days (1 week) prior to the first dose;

5) Vital signs: Monitoring of vital signs is required at each visit; additionally, in the combination treatment group, vital signs should also be monitored during each administration, specifically before the start of SCT-I10A infusion, during the infusion, before the start of SCT510 infusion, during the infusion, and within 1 hour after the infusion ends.
6) Baseline weight must be recorded within 3 days prior to the first dose; If the patient's weight changes by less than 10% compared to the weight reference value corresponding to the current dose during the study, no adjustment of the SCT510 dose is necessary; if the weight change is 10% or more, the SCT510 dose needs to be recalculated;
7) If urinary protein ≥ 2+ in urinalysis, an additional 24-hour urinary protein quantification is required. Blood biochemistry indicators should include amylase and lipase; specific laboratory test indicators can be found in section 6.3.7.6.3.7
8) Results from the screening period within 3 days prior to the first study medication are acceptable.
9) Assessment before medication every 2 cycles, such as on the 1st day of cycles 3, 5, 7, 9, etc., with a time window of ±3 days.
10) Results within 28 days prior to the first administration are acceptable.
11) Pregnancy test: For women who are not postmenopausal or have not undergone surgical sterilization, blood pregnancy (serum β-human chorionic gonadotropin (β-hCG) pregnancy) tests should be conducted during the study screening period (if applicable, including repeat testing before the first study medication) and at the end of treatment visit. Urine/blood pregnancy results are acceptable during treatment (once every 2 cycles ±3 days).
12) Immunological tests (results obtained within 28 days prior to the first administration are acceptable): Anti-HIV, Anti-HCV and/or HCV-RNA, hepatitis B five items (HBsAg, HBsAb, HBeAg, HBeAb, HBcAb) and/or HBV-DNA;
13) For patients who are positive for HBsAg and/or HBcAb during the screening period, HBV-DNA levels should also be measured during the screening period, as well as within 7 days prior to every 2 cycles of treatment and at the end of treatment visit; For patients who are positive for Anti-HCV, HCV-RNA levels should also be measured during the screening period, as well as within 7 days prior to every 2 cycles of treatment and at the end of treatment visit;
14) AFP: Only applicable to Phase III studies, obtained within 7 days prior to randomization, with AFP checks conducted every 2 cycles (±7 days) during the screening period and after treatment, until disease progression, inability to benefit clinically, initiation of new antitumor therapy, withdrawal of informed consent, death, or loss to follow-up, whichever occurs first;
15) Tumor assessment: In Phase II and III studies, investigators conduct regular clinical imaging assessments of tumors during the screening period and after treatment according to RECIST v1.1, including imaging examinations of the chest, abdomen, pelvis, and affected areas. During this study, tumor assessment is recommended to prioritize enhanced CT scans. If patients are allergic to CT contrast agents, a CT chest scan without contrast may be used along with MRI of other areas (e.g., abdomen). For patients with suspected brain metastasis or bone metastasis at baseline, consideration should be given to performing enhanced CT and/or MRI of the brain and bone scans separately. For positive areas related to bone scans (as determined by the investigator), evaluation must be conducted using enhanced CT or MRI. During the entire study period, the same method used during the baseline period was employed to examine the patients, ensuring the consistency and comparability of the images. For patients without contraindications for enhancement, dual-phase scanning images during the hepatic arterial phase and portal phase must be obtained. Routine tumor imaging assessments should be conducted within 28 days prior to the first administration of the study drug during the screening period; if routine tumor imaging assessments were performed before the signing of the informed consent form and within 28 days prior to the first administration, there is no need to repeat during the screening period. From the start of the first dose of the study treatment until 48 weeks, tumor efficacy evaluations will be conducted every 6 weeks (±7 days); after 48 weeks, evaluations will be conducted every 9 weeks (±7 days). The timing of efficacy evaluation visits is fixed until disease progression occurs with no clinical benefit, a new antitumor therapy is initiated, informed consent is withdrawn, death occurs, or loss to follow-up, with the earliest event taking precedence. For the Phase III clinical study, efficacy evaluations will be conducted periodically by BICR according to the RECIST v1.1 and mRECIST criteria.
16) After the end-of-treatment visit, patients who terminate study treatment for reasons other than disease progression, initiation of new antitumor therapy, death, or loss to follow-up should continue to receive tumor assessments during the survival follow-up period until disease progression, initiation of new antitumor therapy, withdrawal of informed consent, death, or loss to follow-up occurs (whichever comes first).

17) Immunogenicity blood sample collection time: patients should have blood samples collected at cycles 1, 2, 4, 8, 12, 16, and subsequently every 8 cycles (such as cycle 24, 32, etc.) and at the end-of-treatment visit, with the collection time being within 1 hour prior to the start of SCT-I10A infusion.
18) Pharmacokinetics blood sampling: Collect blood samples from the first 25 patients enrolled at designated research centers in the Phase II study, as well as from the first 150 patients in the Phase III study experimental group. Blood samples will be collected from the opposite side of the infusion site, with specific sampling times detailed in a separate pharmacokinetics sampling process document.Table2
19) Applicable only to the Phase III study; if the patient has not received investigational drug treatment within 3 days after randomization, it will be considered a randomization failure and the patient will be withdrawn from the study.
20) SCT-I10A intravenous administration: The dosing regimen is 200mg/Q3W, with no time window allowed in the first week, and a time window of ±3 days for the remaining weeks. The duration of medication should not exceed 2 years, until disease progression (PD) occurs (after disease progression, it can be decided based on the patient's condition whether to continue SCT-I10A treatment until no clinical benefit can be obtained), intolerable toxicity, withdrawal of informed consent, death, or loss to follow-up.
21) SCT510 intravenous administration: The dosing regimen is 15 mg/kg/Q3W, with no time window allowed in the first week, and a time window of ±3 days for the remaining weeks. The duration of medication should not exceed 2 years, until disease progression with no clinical benefit, intolerable toxicity, withdrawal of informed consent, death, or loss to follow-up;
22) Oral administration of Sorafenib: Only in Phase III studies, the dosing regimen is 400 mg bid/Q3W for continuous administration, taken in the morning and evening (with an interval of approximately 12 hours) on an empty stomach (1 hour before meals or 2 hours after meals). The duration of medication should not exceed 2 years, until disease progression with no clinical benefit, intolerable toxicity, withdrawal of informed consent, death, or loss to follow-up;
23) Biological sample collection: Only for Phase III patients, tumor specimens from patients will be collected during the screening period (if available) for PD-L1 immunohistochemistry testing. Patients may choose to provide fresh biopsy tissue or the most recent archived tumor tissue (approximately 3 paraffin-embedded sections; specimen requirements are detailed in the central laboratory's operation manual). If tumor tissue cannot be obtained for various reasons (e.g., exhausted due to prior diagnostic testing, high risk of puncture due to tumor location, etc.), it may not be provided;
24) Quality of life assessment: Only for Phase III studies, results must be completed by the patient within 3 days prior to the first study medication administration; Subsequent study treatment will be administered once every 2 cycles (window period ±7 days) and completed by the patient during the end-of-treatment visit;
25) Adverse events: All adverse medical events occurring from the time the patient receives the investigational drug until 90 days after the last dose or before the start of any new antitumor therapy will be collected, prioritizing the earliest occurrence. After this period, only serious adverse events related to the investigational drug or study procedures need to be collected.

Table2 Pharmacokinetic Blood Collection Process

**Phase II study (25 patients enrolled at designated research centers)**		
**Intensive sampling during administration in cycles 1 and 4**		
Sample collection time points	Time window	Pharmacokinetics
Before SCT-I10A infusion begins	-1 hour	X
Immediately after SCT-I10A infusion completion	+5 minutes	X
Immediately after SCT510 infusion completion	+5 minutes	X
6 hours after SCT-I10A infusion begins	±30 minutes	X
24 hours after SCT-I10A infusion begins (Day 2)	±1 hour	X

96 hours after SCT-I10A infusion begins (Day 5)	±2 hours	X
168 hours after SCT-I10A infusion begins (Day 8)	±24 hours	X
336 hours after SCT-I10A infusion begins (Day 15)	±24 hours	X
504 hours after SCT-I10A infusion begins (Day 22)	±24 hours	X
**Sampling at cycles 2, 5, 6, 8, and 12**		
Sample collection time points	Time window	Pharmacokinetics
Before SCT-I10A infusion begins	-1 hour	X
Note: 1) The sample collection time points for cycles 2 and 5 overlap with day 22 of the intensive sampling in cycles 1 and 4, and no additional collection is needed; However, if there is a delay in administration during cycles 2 or 5 due to AE or other reasons, PK blood samples must be collected within 1 hour prior to the start of SCT-I10A infusion in cycles 2 and 5.<br>2) If a patient terminates treatment early between cycles 5 and 11, a PK blood sample should be collected at the end-of-treatment visit.		
**Phase III study (only for the first 150 patients in the experimental group)**		
**Cycle 1**		
Before SCT-I10A infusion begins	-1 hour	X
Immediately after SCT510 infusion completion	+5 minutes	X
**Cycles 2, 4, 8, and 12**		
Before SCT-I10A infusion begins	-1 hour	X
Note: If a patient terminates treatment early between cycles 5 and 11, a PK blood sample should be collected at the end-of-treatment visit.		

1 Research background

1.1 Overview of the epidemiology and treatment status of hepatocellular carcinoma

1.1.1 Epidemiological characteristics

Primary liver cancer is the sixth most common cancer in the world, with approximately 840,000 new cases and 780,000 deaths each year, of which China accounts for 466,000 new cases and 422,000 deaths annually. Therefore, the incidence and mortality rates of liver cancer in our country exceed more than half of the global total. Among liver cancers, 75-85% are hepatocellular carcinoma (HCC). Chronic hepatitis B virus (HBV) or hepatitis C virus (HCV) infection, aflatoxin contamination, alcoholism, obesity, and 2 type diabetes are the main risk factors, but their etiology varies significantly across regions, with HBV infection and aflatoxin exposure being the primary causes in China [2, 3]. Liver cancer is currently the 4th most common malignant tumor in our country and the 2nd leading cause of cancer-related deaths, posing a serious threat to the lives and health of our people [4].

With advancements in diagnostic and treatment methods, the prognosis for early-stage liver cancer patients has improved. For patients with early-stage disease, treatment options include surgical resection, local ablation, and liver transplantation; however, approximately 2/3 of patients cannot benefit from these treatments [5], as they are often diagnosed at intermediate or advanced stages, making them unsuitable for surgery or local therapy, and frequently have underlying conditions, resulting in generally poor prognosis for this group. In this chapter, the term 'liver cancer' refers to HCC unless otherwise specified.

1.1.2 Current status of first-line treatment for hepatocellular carcinoma

Sorafenib is the world's first multi-target kinase inhibitor approved for the treatment of patients with advanced liver cancer who cannot undergo surgical resection. Based on the Sharp and Oriental international phase III clinical studies, Sorafenib has established its standard treatment position for advanced liver cancer. Sharp is a multicenter, randomized, double-blind, placebo-controlled clinical study conducted in Europe and the United States, with the primary endpoints being overall survival and time to symptom progression. Overall survival was significantly extended, 10.7 vs 7.9 months, with no improvement in time to symptom progression. The median TTP was 5.5 vs 2.8 months, but the ORR was only 2%. Oriental is a randomized, double-blind, placebo-controlled clinical study conducted in the Asia-Pacific region, OS extended 2.3 months (6.5 vs 4.2 months) [6, 7]. Due to the complex molecular pathogenesis of HCC and

the absence of major driver genes, multiple studies on new targeted drugs conducted in the nearly 10 years following Sorafenib have all failed, with OS not superior to Sorafenib.

Lenvatinib is the second drug approved for first-line treatment of unresectable hepatocellular carcinoma following Sorafenib, based on the REFLECT study (a non-inferiority trial comparing Lenvatinib to Sorafenib), with the primary endpoint being OS. Lenvatinib and Sorafenib had 13.6 vs 12.3 months, and the secondary endpoints were significantly better than Sorafenib, with median PFS of Lenvatinib and Sorafenib being 7.4 vs 3.7 months, and ORR of 24% and 9% respectively [8].

In addition to targeted therapy, based on the EACH study, the CFDA approved the FOLFOX regimen for the treatment of liver cancer in 2013, and the NCCN guidelines recommend it as a category 2B option for first-line treatment of advanced hepatocellular carcinoma [9]. Overall, the OS for first-line treatment of advanced hepatocellular carcinoma is 6.4-13.6 months. Although these drugs improve OS, their benefits remain limited, and the need to extend survival and improve tolerance has yet to be met; the treatment options for first-line therapy in patients with advanced liver cancer are very limited, and these patients urgently need new treatment strategies.

With the development of biological agents, immunotherapy has fundamentally changed the landscape of tumor therapy by targeting the host immune system. Immune checkpoint inhibitors, such as cytotoxic T lymphocyte-associated antigen 4 (CTLA-4), programmed cell death -1 (PD-1), and their ligands (PD-L1 or B7-H1), have been shown to be effective against various solid tumors. The efficacy of immunotherapy appears to be related to the immune microenvironment of the tumor and its immunogenicity. Recent research results indicate that targeting immune checkpoints may become a future option for the treatment of hepatocellular carcinoma.

1.1.3 Immunotherapy for advanced hepatocellular carcinoma

Programmed death factor 1 and its ligand (PD-1/PD-L1) are a pair of immune co-stimulatory factors among immune checkpoint inhibitors. Under normal circumstances, PD-1 exerts immune regulatory effects through its ligand PD-L1. In recent years, PD-1 and its ligand PD-L1 have attracted attention due to their involvement in tumor immune evasion mechanisms. The activation of the PD-1/PD-L1 signaling pathway can lead to the formation of an immunosuppressive tumor microenvironment, allowing tumor cells to evade immune surveillance and destruction. Blocking the PD-1/PD-L1 signaling pathway can reverse the tumor immune microenvironment and enhance endogenous anti-tumor immune effects. PD-L1 is highly expressed in various solid malignant tumors, including non-small cell lung cancer, melanoma, renal cell carcinoma, prostate cancer, breast cancer, and glioma. Its expression levels vary

depending on the tumor type, and it not only promotes tumor cell growth but can also induce the apoptosis of T lymphocytes. Currently, PD-1/PD-L1 antibodies have shown good efficacy in the treatment of various types of tumors.

The correlation between the response rate of immune checkpoint inhibitors (CPIs, check-point inhibitors) and PD-L1 expression levels has been confirmed in various types of tumors; therefore, studies have also been conducted to determine whether PD-L1 expression levels are related to the pathology of liver cancer and patient prognosis. Although the results obtained are controversial, the first meta-analysis conducted by Gu et al. concluded that high PD-L1 expression can predict poor differentiation, higher AFP levels, vascular invasion, and worse survival. The expression of PD-L1 in peritumoral hepatocytes is also an independent prognostic factor for OS and DFS, suggesting that future cancer treatments should target not only the residual tumor cells but also the 'soil' that promotes tumor growth. Circulating PD-L1 levels also have certain significance for the prognosis of liver cancer patients. Finkelmeier et al. conducted a prospective cohort study to investigate the relationship between soluble PD-L1 levels and liver disease and HCC staging, concluding that high soluble PD-L1 levels may be an indicator of poor prognosis in HCC. Overall, the expression of tumor cells and peritumoral liver cells PD-L1 is associated with poorer survival, and even in the adjuvant therapy after liver cancer resection, PD-L1 may be a good target [10].

On September 23, 2017, Nivolumab received accelerated approval from the U.S. Food and Drug Administration (FDA) for patients with hepatocellular carcinoma (HCC) who had previously been treated with Sorafenib, becoming the first immunotherapy drug approved for advanced HCC. This accelerated approval was based on the Checkmate-040 study, where Nivolumab demonstrated good efficacy against liver cancer, with known adverse reactions similar to those observed in other tumors, and no new safety issues were identified. Based on the Checkmate-040 study, the subsequent Checkmate-459 study [11] (2019 ESMO) was conducted, which was a global Phase III study comparing Nivolumab head-to-head with Sorafenib as first-line treatment for advanced HCC. The primary endpoint OS for Nivolumab compared to Sorafenib was 16.4 vs 14.7 months, which did not meet the preset criteria, while the PFS for both groups was similar, at 3.7 months and 3.8 months, respectively. Compared to Sorafenib, the Nivolumab treatment group had a higher objective response rate (15% vs 7%). Compared to Sorafenib, the safety of the Nivolumab treatment group is higher, and the quality of life for patients is better. Although not statistically significant, Nivolumab showed better tolerance and clinically meaningful improvements in overall survival (OS) compared to Sorafenib, as well as overall and

complete response rates as a first-line treatment, indicating the potential benefits of immunotherapy as a first-line treatment for this aggressive cancer.

Following Nivolumab, based on the results of the Keynote-224 study [12], Pembrolizumab is the second PD-1 inhibitor conditionally approved by the FDA for the treatment of previously treated advanced HCC. The Keynote-224 study is a Phase II single-arm clinical trial of Pembrolizumab monotherapy for advanced HCC after failure of Sorafenib treatment. The median progression-free survival (mPFS) for 104 patients was 4.9 months, and the median overall survival (mOS) was 12.9 months. Unfortunately, the results of the Keynote-240 study [13], a confirmatory Phase III clinical trial of Pembrolizumab for second-line treatment of HCC, announced at the ASCO conference in June 2019, did not meet the pre-set statistical standards for both primary endpoints, resulting in failure. Although immunological therapy has potential benefits in hepatocellular carcinoma, the efficacy of monotherapy is limited, necessitating the pursuit of greater breakthroughs in combination therapies.

1.1.4 Immune checkpoint inhibitors combined with anti VEGF targeted therapy

1.1.4.1 The theoretical basis for immune checkpoint inhibitors combined with anti VEGF targeted therapy

In addition to promoting tumor angiogenesis, increasing evidence suggests that VEGF plays a role in cancer immune evasion through various mechanisms. For example, activated endothelial cell experiments indicate that in the tumor microenvironment, lymphocytes may reduce adhesion to the vascular wall, thereby decreasing the recruitment of immune cells to the tumor site. Inhibiting VEGF signaling can reverse certain immunosuppressive activities of VEGF. Therefore, dendritic cell function in mice exposed to pathological levels of VEGF is impaired, and blocking VEGF2 can restore dendritic cell function [14]. In a mouse melanoma model, VEGF blockers synergize with acquired immunotherapy, resulting in enhanced antitumor activity, prolonged survival, and increased transport of T cells into the tumor [15]. Vascular endothelial growth factor-mediated immune suppression primarily occurs through 3 key mechanisms: inhibiting dendritic cell maturation, reducing T cell tumor infiltration, and promoting the proliferation of suppressive cells in the tumor microenvironment [16].

Research indicates that anti-PD-1/PD-L1 therapy can make tumors sensitive to anti-angiogenic treatment and prolong its efficacy. CURRAN et al. found that in studies of combined anti-CTLA-4 and anti-PD-1 therapy, the quantity and activity of T cells changed, the pericyte coverage of tumor blood vessels increased, vascular perfusion improved, and permeability decreased, leading to a tendency for vascular normalization. Therefore, the increase and activation

of tumor-infiltrating effector T cells can not only reshape the tumor microenvironment (TME) but also normalize the vasculature. In summary, tumor vascular normalization can promote the aggregation of immune cells and enhance immune function, while the activation of immune cells can, in turn, promote vascular normalization; thus, the two form a positive feedback loop. This mechanism provides a theoretical basis for the combination of anti-angiogenic therapy and immune checkpoint inhibitors in the treatment of malignant tumors [17].

1.1.4.2 Clinical application of immune checkpoint inhibitors combined with anti VEGF targeted therapy in hepatocellular carcinoma

The anti-VEGF monoclonal antibody Bevacizumab has shown a survival advantage in various solid tumors and was approved by the U.S. Food and Drug Administration in 2004. In addition to its anti-angiogenic properties, Bevacizumab's immunomodulatory characteristics may also play a role in its clinical activity. Therefore, immunotherapy combined with Bevacizumab may enhance anti-tumor immune responses, thereby improving and prolonging clinical benefits.

Based on the results of the IB phase (GO30140 study) [18], patients receiving combination therapy with Atezolizumab and Bevacizumab demonstrated good safety and anti-tumor activity, with an ORR of 36%, mPFS of 7.3 months, and OS of 17.1 months. The subsequent IMbrave150 study [19] was a Phase III international multicenter study evaluating the use of Atezolizumab combined with Bevacizumab for the treatment of patients with unresectable liver cancer who had not previously received systemic therapy. Patients were randomly assigned in a ratio of 2: 1 to receive Atezolizumab and Bevacizumab combination therapy or Sorafenib treatment. Ann-Lii Cheng presented the study results at the 2019 European Society for Medical Oncology Asia Congress (ESMO-ASIA), reporting that the median OS for the Atezolizumab and Bevacizumab group had not yet been reached, while the median OS for the Sorafenib group was 13.2 months (HR= 0.58 ; 95%CI: 0.42~0.79). In terms of PFS, the median PFS for Atezolizumab combined with Bevacizumab was 6.8 months, while the median PFS for the Sorafenib group was 4.3 months (HR=0.59;95% confidence interval [CI]: 0.47~0.76). In terms of ORR, the ORR for Atezolizumab combined with Bevacizumab reached 27%, with a complete response rate of 6%. Subsequently, at the 2020 Liver Cancer Summit, the research data from the IMbrave150 Chinese cohort was published, showing that compared to Sorafenib, the “T+A” combined treatment demonstrated statistically significant and clinically meaningful improvements in overall survival (OS) and progression-free survival (PFS). In the Chinese cohort: Compared to Sorafenib, the combination treatment of 'T+A' reduced the risk of death (OS) by 56% (HR=0.44, 95%CI:0.25-0.76), and reduced the risk of disease progression or death (PFS) by 40% (HR=0.60, 95%CI:0.40-

0.90). Compared to global study data, the benefits in OS and PFS in the Chinese cohort are generally consistent with global trends and levels [1]. However, in terms of OS and reduced mortality risk, 'T+A' reduced the risk of death by 56% in the Chinese cohort, while the global study showed a reduction of 42%, suggesting that it may provide greater benefits for Chinese patients. In terms of safety, IMbrave150 study shows that the overall tolerance in the “T+A” group is good, with the treatment-related adverse event rate being 84% vs 94% compared to the Sorafenib group, but most were Grade 1-2 adverse events. Among patients receiving the “T+A” combination therapy, 36 % experienced Grade 3-4 treatment-related adverse events, while 46 % of patients receiving Sorafenib treatment experienced Grade 3-4 treatment-related adverse events. The combination therapy is generally well tolerated and the toxicity is manageable, with common adverse events primarily including hypertension, proteinuria, diarrhea, abdominal pain, decreased appetite, fever, elevated ALT, infusion reactions, etc. No new safety issues were found apart from the known safety events of Atezolizumab and Bevacizumab monotherapy. The safety profile in the Chinese cohort is generally consistent with global study data. In addition, the safety of immunotherapy + anti-angiogenic therapy combined / not combined with chemotherapy has also been demonstrated in several other tumor types.

Therefore, we can conclude that the combination of immunotherapy and anti-angiogenic therapy can bring more significant clinical benefits and has good tolerance. The efficacy of PD-1 inhibitors combined with Bevacizumab in the treatment of advanced hepatocellular carcinoma deserves further exploration. SCT510 and SCT-I10A are recombinant humanized anti-VEGF monoclonal antibody injection and recombinant humanized anti-PD-1 monoclonal antibody developed by Sinocelltech Ltd. In order to further explore the efficacy and safety of SCT-I10A combined with SCT510 as first-line treatment for unresectable, recurrent, and/or metastatic hepatocellular carcinoma, this study was initiated.

1.2 SCT-I10A

1.2.1 SCT-I10A Overview

SCT-I10A, a recombinant humanized anti-PD-1 monoclonal antibody developed by Sinocalltech Ltd., is a humanized IgG4 monoclonal antibody consisting of 1326 amino acids, with a molecular weight of approximately 145kD. SCT-I10A can specifically bind to PD-1 on the surface of cells, altering the tumor microenvironment by blocking the interaction between PD-1 and its ligands, thereby restoring and enhancing the immune killing function of T cells, which inhibits tumor growth. SCT-I10A is obtained from CHO DG44 cells integrated with the PD-1 antibody expression gene, cultured in serum-free suspension, and purified through steps such as

protein A affinity chromatography and ion exchange chromatography to remove process-related impurities, product-related impurities, and other exogenous factors, resulting in a purified antibody, which is then concentrated by ultrafiltration and formulated into an SCT-I10A injection preparation with an appropriate stabilizer and sterilized. SCT-I10A is a sterile, colorless, slightly opalescent liquid formulation without preservatives, intended for intravenous infusion in clinical settings.

1.2.2 Experience from preclinical pharmacodynamics studies of SCT-I10A

The in vitro pharmacodynamics of SCT-I10A were studied from several aspects including antibody binding to antigen epitopes, protein binding/affinity, ligand binding blockade, activation of mixed lymphocyte reactions, activation of PD-1 reporter gene systems, antibody-dependent cellular cytotoxicity (ADCC), CDC and FcRn binding. The results showed: 1)SCT-I10A has high affinity, with a dissociation constant (Kd) of 64.8 pM, which can effectively block the binding of PD-L1 ligand protein to PD-1, activating tumor immune tolerance T cells to kill tumor cells; 2) SCT-I10A has better in vitro activity than Nivolumab ; 3)SCT-I10A exhibits lower ADCC and very low CDC function, further ensuring good efficacy and safety in clinical trials; 4)SCT-I10A binds well to FcRn, ensuring the drug's metabolic levels in the body.

In vivo pharmacodynamic studies used a humanized PD-1 C57BL/6 mouse model with MC38 subcutaneous transplanted tumors to investigate the anti-tumor effect of SCT-I10A alone on humanized PD-1 mouse MC38 subcutaneous transplanted tumors. The research results show that SCT-I10A alone (at 2, 8, and 20mg/kg, administered once every 3 days for a total of 6 administrations) can significantly inhibit the growth of MC38 colon cancer xenografts.

1.2.3 Experience in preclinical pharmacokinetics studies of SCT-I10A

Using crab-eating macaques as models, a single-dose pharmacokinetics trial was conducted, setting three dosage groups: high (10mg/kg), medium (3mg/kg), and low (1mg/kg), with 6 animals in each group, evenly split between males and females, administered via intravenous infusion. The results show that SCT-I10A was administered via intravenous infusion at dosages of 1, 3, and 10mg/kg to crab-eating macaques, and the serum drug concentration increased with the dosage. No significant gender differences were observed in the blood drug concentration in the animals. Starting from 336h post-administration, the drug concentration decreased significantly, which may be attributed to the development of drug-resistant antibodies in some animals.

The pharmacokinetic results indicate:

1) C_{max} and AUC increased with the dosage. The average terminal elimination half-life across dosage groups ranged from 128.50-215.62h, and the average residence time ranged from 92.09-217.85h.

2) The ratios of average C max for male and female animals in each dosage group were 1: 2.81: 11.26 and 1: 3.37: 11.99, while the ratios of average AUC last were 1: 4.02: 16.33 and 1: 3.64: 14.45. Within the dosage range of 1~10mg/kg, the test substance exhibited basic linear pharmacokinetic characteristics in the crab-eating macaque.

3) SCT-I10A was intravenously infused at dosages of 1, 3, and 10mg/kg to the crab-eating macaque, starting from 2 weeks, where some animals in each dosage group showed rapid decline in blood drug concentration. Results confirmed by drug-resistant antibodies (ADA) show that animals with declining drug concentration have developed high titers of drug-resistant antibodies, and in some cases, the drug concentration in the blood has fallen below the detection limit. Calculating the pharmacokinetic parameters of animals in each group, the MRT (mean residence time) of female animals in the 1 and 3mg/kg dosage groups is slightly higher than that of male animals, indicating that the incidence and titer levels of drug-resistant antibodies in male animals are higher than those in female animals. The level of drug metabolism in the blood of animals is directly related to the production of ADA.

The results of T cell receptor occupancy show that the PD-1 receptor occupancy on the surface of CD8+ mature T cells in each dosage group reaches saturation at 2h after administration, remains at a plateau within 168h, and then gradually declines in a dose-dependent manner. Among them, the high dosage group (10mg/kg) at 336 hours post-administration, or 2 weeks, showed that all animals except one maintained receptor occupancy in a saturated state, while the animals with a decreasing trend in blood concentration still had a receptor occupancy rate above 50%. Based on body surface area conversion to equivalent dosage, this dosage corresponds to the clinical human intended dosage of approximately 3mg/kg. Furthermore, considering the clinical intended dosing frequency of once every 2-3 weeks, and the analysis of preclinical T cell receptor occupancy study results, during repeated dosing in clinical studies, the target drug binding remains in a saturated state, which can maximally ensure the clinical efficacy of the drug.

1.2.4 SCT-I10A Preclinical toxicology study

The clinical intended dosage of SCT-I10A is 1~5mg/kg, administered intravenously once every 2~3 weeks, for the treatment of malignant tumors. Under GLP conditions, a general pharmacology study, single-dose acute toxicity study, repeated-dose toxicity study (including

toxicokinetics), hemolytic and local irritation studies, tissue cross-reactivity, and immunogenicity /immunotoxicity studies of SCT-I10A were conducted.

The general pharmacology study of SCT-I10A was combined with long-term toxicity tests to evaluate the effects of the drug on animal body temperature, blood pressure, electrocardiogram, respiratory system, mental state, and behavioral activity. The results showed SCT-I10A had no significant effects on the central nervous system, cardiovascular system, and respiratory system of the crab-eating macaques.

A single acute toxicity test was conducted in crab-eating macaques, with intravenous infusion administered at 30, 100, and 300mg/kg across three dosage groups. Clinical observations in each group did not reveal any abnormalities related to the administration. The maximum tolerated dose (MTD) for crab-eating macaques is greater than 300mg/kg. The long-term toxicity study employed a weekly dosing regimen that is higher than the clinical intended dosing frequency, which can increase drug exposure in a short period to observe potential toxic reactions in the body. In this study, intravenous infusion was administered in crab-eating macaques using a method consistent with clinical administration routes. A negative control group and three dosage groups (3, 20, and 100mg/kg) were established, with continuous administration for 13 weeks followed by a recovery period of 8 weeks. The results showed no unplanned deaths or near-death incidents, and no drug-related abnormal reactions were observed during clinical monitoring, with no immune-related toxicity noted.

The aforementioned hematological drug-related abnormal changes were minor and had all recovered by the end of the recovery period, indicating no toxicological significance. Additionally, the observed infiltration of inflammatory cells in the renal cortex and/or medullary interstitium, as well as in the salivary gland interstitium, had all recovered by the end of the recovery period.

Toxicokinetic results indicate that within the 3~100mg/kg dosage range, SCT-I10A shows a certain degree of accumulation in the animal body. In the immunogenicity study, after administration, some animals in the low, medium, and high dosage groups tested positive for ADA antibodies, with the incidence showing a pattern of low dosage > medium dosage > high dosage. In the low dosage group, antibody titers increased in a time-dependent manner and were higher than those in the medium and high dosage groups, with no significant decrease observed at the end of the 8 -week recovery period. Some ADA exhibited neutralizing activity, and combined with the TK results, it can be seen that the production of high-titer ADA antibodies may reduce systemic exposure in individual animals, but the overall blood drug concentration in the high dosage group remained higher than that in the medium and low dosage groups.

The results of the tissue cross-reactivity show that the biotin-labeled test samples (SCT-I10A/Biotin, 2.0μg/mL and 10.0μg/mL) specifically bind to the lymphocytes of the immune system in normal crab-eating macaques, including those in the spleen, lymph nodes, thymus, and tonsils, as well as the bronchus-associated lymphoid tissue in the lungs and the gut-associated lymphoid tissue in the colon, ileum, and stomach. Under the same conditions, SCT-I10A specifically binds to the lymphocytes in the lymph nodes, spleen, and thymus of normal human tissues, as well as the bronchus-associated lymphoid tissue in the lungs and the gut-associated lymphoid tissue in the stomach, ileum, and colon.

In summary, SCT-I10A does not cause toxicologically significant drug-related adverse reactions in crab-eating macaques, although it can induce strong immunogenicity in vivo, it does not exhibit immunotoxicity. Based on the animal experimental data and clinical safety indications and data of marketed products, this drug is not recommended for use in pregnant women due to its pharmacological effects having side effects on fetal development. Therefore, it is believed that the safety risks associated with the clinical application of SCT-I10A are foreseeable and controllable.

1.2.5 Phase I clinical study data of SCT-I10A

The SCT-I10A-X101 study is a multicenter, open-label, Phase I clinical study designed to evaluate the safety, tolerance, pharmacokinetic characteristics, and preliminary efficacy of the recombinant humanized anti-PD-1 monoclonal antibody injection (SCT-I10A) in patients with advanced solid tumors or lymphomas, divided into two parts: dose exploration and expanded indication enrollment.

In the dose exploration phase, a ‘3+3’ design was used for dose escalation, gradually increasing the SCT-I10A dose levels. The dose escalation phase targets the 200mg dose group, while also designing a low-dose group of 60mg and a high-dose group of 600mg, with a total of three dose groups for escalation. Each dose group initially enrolls 3 patients; if this study does not reach the MTD or if the MTD is 600mg, enrollment in the 600mg dose group will continue until 6 patients are enrolled. Among them, the 200mg, Q3W dose group will continue to enroll patients until 15 patients are included. Additionally, there are 200mg, Q2W dosage group and 3mg/kg, Q3W dosage group, both of which will include 6 patients to evaluate the safety at different dosing frequencies. The starting dose is 60mg, followed by sequential groups of 200mg and 600mg. After confirming the safety of the target dosage 200mg group, an expanded enrollment study part for this dosage group will be conducted simultaneously based on indications. As of June 24, 2020, the SCT-I10A monotherapy for second-line or above advanced solid tumors or lymphomas I

phase clinical study (SCT-I10A-X101) has enrolled 239 patients, and the clinical trial is currently in the patient enrollment and follow-up phase, with final data cleaning and database locking yet to be completed.

1.2.5.1 Human pharmacokinetics

In the SCT-I10A-X101 study, as of November 20, 2019, the PK concentration data that had been detected was used to calculate PK parameters, and the pharmacokinetic characteristics of SCT-I10A were preliminarily evaluated. The pharmacokinetic characteristics of a single dose of SCT-I10A were assessed at four dosage levels: 60mg, 200mg, 3mg/kg, and 600mg; The three dosage groups of 60mg, 200mg, and 600mg will continue into the multiple dosing phase 28 days after the initial administration, with a dosing frequency of once every three weeks; The 3mg/kg dosage group directly enters the multiple dosing mode with a dosing frequency of once every three weeks.

Pharmacokinetic characteristics of single-dose administration at different dosage levels: on 60mg, 200mg, 3mg/kg, and 600mg under the dosing conditions, the study SCT-I10A 's PK characteristics of single-dose administration. The average maximum plasma concentration (C max) for single doses of 60mg, 200mg, 3mg/kg, and 600mg were 22.20, 62.45, 71.86, and 221.33μg/mL, respectively, while the average plasma concentration - time curve area under the curve (AUC 0~last) were 189.22, 578.16, 592.15, and 2119.99μg·day/mL, respectively. The average $t_{1/2}$ range for single-dose administration is 12.25~18.30 days; The clearance rate of SCT-I10A ranges from 178~265 mL/day.

Pharmacokinetic characteristics of multiple-dose administration: The 200mg Q3W dosing regimen has a steady-state C_{max} of 104.63μg/mL, a minimum concentration (C_{min}) of 37.55 μg/mL, a steady-state AUC_{ss} of 1199.34 μg·day/mL, and a steady-state CL_{ss} of 176.56 mL/day. In the Q3W dosing regimen, SCT-I10A reaches steady state at 15 weeks, with an accumulation constant of 1.52.

1.2.5.2 Safety

SCT-I10A-X101 The study concluded on June 24, 2020, with a preliminary assessment of safety conducted on 239 patients, showing a TEAE incidence rate of 86.6%, with a drug-related adverse event incidence rate of 63.6%, and a drug-related severe adverse event incidence rate of 9.2%. The incidence of drug-related adverse events leading to discontinuation of the investigational drug was 7.1%, indicating overall good safety. In the target dosage 200mg dosage group, the TEAE incidence rate was 88.8%, with a drug-related adverse event incidence rate of 65.4%, and the incidence rate of SAE was 24.3%, with a drug-related SAE incidence rate of 8.9%.

The incidence rate of drug-related adverse events of grade ≥3 was 12.1%. Immune-related adverse events with an incidence rate of ≥ 5% included: hyperthyroidism (4.7%), hypothyroidism (10.7%), and rash (4.7%). The overall safety of SCT-I10A-X101 across all dosage groups is shown in Table 3.3

3Table 3 In the SCT-I10A-X101 study, the overall safety profile of the investigational drug SCT-I10A is presented.

**Classification**	**60mg (N=3) n(%)**	**200mg (N=214) n (%)**	**3mg/kg (N=6) n (%)**	**300mg (N=10) n (%)**	**600mg (N=6) n (%)**	**Overall (N=239) n (%)**
TEAE	3(100)	190(88.8)	6(100)	2 (20)	6(100)	207(86.6)
TRAE	1(33.3)	140(65.4)	4(66.7)	2(20.0)	5(83.3)	152(63.6)
SAE	2(66.7)	52(24.3)	1(16.7)	0	3(50)	58(24.3)
TRSAE	1(33.3)	19(8.9)	0	0	2(33.3)	22(9.2)
CTCAE ≥ Grade 3 AE	2(66.7)	65(30.4)	2(33.3)	0	3(50)	72(30.1)
CTCAE ≥ Grade 3 TRAE	1(33.3)	26(12.1)	1(16.7)	0	2(33.3)	30(12.6)
AE leading to discontinuation of the investigational drug	0	33(15.4)	0	0	2(33.3)	3(14.6)
AE leading to discontinuation that are related to the investigational drug	0	16(7.5)	0	0	1(16.7)	17(7.1)
AE that led to the suspension of the investigational drug	1(33.3)	24(11.2)	2(33.3)	0	2(33.3)	29(12.1)
AE related to the investigational drug that led to its suspension	0	19(8.9)	2(33.3)	0	2(33.3)	23(9.6)

Note:
[1] The numbers calculated in each column are the number of patients (incidence rate). Percentages are based on the number of patients in each dosage group and the overall safety analysis set. The analysis of adverse events will be based on adverse events occurring during the treatment period (TEAE).
[2] Adverse events related to the investigational drug are defined as definitely related, possibly related, or indeterminate.

Based on existing safety data, no unexpected safety signals have been found for SCT-I10A compared to the safety data of marketed PD-1 monoclonal antibodies.

1.2.5.3 Efficacy

As of June 24, 2020, a total of 216 patients in the SCT-I10A 200mg/Q3W and 3mg/kg/Q3W dosage groups received treatment with the investigational drug, with an overall ORR of 15.74% (34/216). The preliminary ORRs for various solid tumors were as follows: gastric cancer at 16.13%, esophageal cancer at 18.18%, nasopharyngeal cancer at 31.25%, small cell lung cancer at 23.81%, and ovarian and fallopian tube cancer at 18.18%. Preliminary efficacy results indicate that SCT-I10A in the 200mg/Q3W dosage group has comparable antitumor activity to similar drugs.

As of February 5, 2020, the SCT-I10A-X101 Phase I study has enrolled 15 patients with liver cancer who have failed first-line or higher treatments. Preliminary efficacy data show: ORR is 13.33% (2/15), DCR is 33.33% (5/15), indicating that SCT-I10A shows clinical efficacy for liver cancer. However, due to the small sample size, the clinical efficacy of SCT-I10A for advanced hepatocellular carcinoma needs further validation in subsequent trials.

1.3 SCT510

1.3.1 Overview of SCT510

SCT510, a recombinant monoclonal antibody against vascular endothelial growth factor developed by Sinocelltech Ltd. (internal project code SCT510), is a humanized IgG1 type monoclonal antibody (mAb), with the same antigen binding site and amino acid sequence as the imported product Bevacizumab (trade name Aflibercept®) available in China. Thus, to maximize the effectiveness and safety of SCT510 in clinical applications and reduce its development risks. From product initiation to the entire research and development process, we have focused on a series of comparative studies between SCT510 and Aflibercept®in terms of pharmacology, preclinical pharmacokinetics, in vitro and in vivo pharmacodynamics, and safety. Preclinical research results indicate that SCT510 and Aflibercept ® are fundamentally consistent in terms of antibody structure, physicochemical properties, and in vitro biological activity, and both show a high degree of similarity in pharmacokinetics, in vitro and in vivo pharmacodynamics, and safety.

1.3.2 Pharmaceutical comparative study

SCT510 and Aflibercept®exhibited similarities in biological activity, physicochemical indicators, characterization analysis, impurity residue, and high-temperature stability. Peptide mapping analysis, mass peptide mapping analysis, C -terminal sequence analysis, and N -terminal sequence analysis indicated that the recombinant humanized anti- VEGF monoclonal antibody is consistent with the sequence of Aflibercept ® and aligns with the theoretical sequence; The two products showed consistent results in mass spectrometry molecular weight, free thiol detection, disulfide bond analysis, and isoelectric point detection; Similarities were observed in electrophoresis, SEC, DLS, and other physicochemical analyses; In terms of biological activity, the experiments on the binding with VEGF165 and the detection of cellular biological activity indicate that the biological activities of the two varieties are similar; In the N- glycan analysis, the glycosylation of SCT510 and Aflibercept ® shows consistency in overall trends, with the main glycoforms being G0F and G1F. Although the high mannose-type glycosylation content of SCT510 is slightly higher, no differences were observed between the two in non-clinical

pharmacokinetic studies. Since the antitumor mechanisms of SCT510 and Aflibercept ® are unrelated to antibody-dependent cell-mediated cytotoxicity and complement-dependent cytotoxicity, meaning they do not involve Fc receptors and complement effects, the differences in glycosylation will not have a significant impact on antibody activity. In terms of charge isomers, CEX-HPLC and cIEF results show that the acidic peak content of SCT510 is lower than that of Aflibercept ®, with performance slightly better than the marketed product Aflibercept ®.

1.3.3 Preclinical efficacy comparison study

SCT510 primarily exerts its effects by specifically binding to the VEGF secreted by tumor cells, neutralizing its binding to the surface receptors of vascular endothelial cells (VEGFR), thereby blocking the phosphorylation of intracellular tyrosine kinases of VEGFR and downstream signal transduction, ultimately inhibiting tumor angiogenesis and exerting an anti-tumor growth effect. The results of the in vitro pharmacodynamic experiments show that SCT510 has a good similarity to Aflibercept®. The affinities of both for human VEGF are similar, at 0.358nM and 0.412nM, respectively. They similarly compete to inhibit the binding of VEGF165 to its receptors VEGFR1 and VEGFR2, and can inhibit the proliferation and survival of endothelial cells. The ELISA results show that the EC50 values for both binding to FcRn are 24.6 and 11.7μg/mL, respectively; although there is a slight difference, no differences were observed in the pharmacokinetic studies in crab-eating macaques.

The in vivo pharmacodynamics study used BALB/c-nu nude mice with transplanted tumors as the animal model to investigate the anti-tumor effect of SCT510 alone on human xenograft tumors, and compared it with the marketed drug Aflibercept®. The human tumor cell lines used included VEGF -positive human rhabdomyosarcoma A673 cells, colon cancer LS174T cells, and lung cancer NCI-H460 cells. The results showed that the anti-tumor effects of SCT510 and Aflibercept®were comparable at the same dosage, with no significant differences. In addition to its anti-tumor effects in vivo, SCT510 can also inhibit angiogenesis in transplanted tumors, and its anti-angiogenic effect is comparable to that of Aflibercept®with no statistically significant difference.

1.3.4 Preclinical pharmacokinetics comparative study

The pharmacokinetic characteristics of SCT510 in crab-eating macaques were studied using the ELISA method and compared with the marketed drug Aflibercept ®. The results showed that there were no significant differences in the pharmacokinetic parameters of SCT510 and Aflibercept ® once administered. Crab-eating macaques received single intravenous injections of 2, 10, 50 mg/kg SCT510 and 10 mg/kg Aflibercept ®, with blood samples collected immediately

before and after administration, as well as at 1, 4, 8, 24 h, 2, 3, 5, 7, 9, 11, 14, 17, 21, 24, and 28 days post-administration, followed by plasma separation. The concentrations of SCT510 and Aflibercept ® in plasma were detected using a validated ELISA method. The results showed that a single slow intravenous infusion of SCT510 within the dosage range of 2 to 50mg/kg led to an increase in plasma drug concentration with increasing dosage, with the low, medium, and high dosage groups having a dosage ratio of 1:5:25, and the ratios of C max and AUC last being 1:4.22:27.86 and 1:4.45:23.41, respectively, indicating a positive correlation between plasma drug concentration and plasma drug exposure with the dosage. The half-lives of the low, medium, and high dosage groups of SCT510 and the Aflibercept® group were 175.16 ± 27.07, 173.85 ± 20.14, 172.28 ± 54.24, and 184.85 ± 55.66 hours, respectively, with clearance rates of 0.18 ± 0.03, 0.21 ± 0.05, 0.20 ± 0.06, and 0.20 ± 0.03 mL/h/kg, respectively. The results indicate that within the dosage range of 2 to 50 mg/kg, SCT510 exhibits a basically linear pharmacokinetic profile in crab-eating macaques, and the pharmacokinetic characteristics of SCT510 and Aflibercept® at the same dosage (10 mg/kg) are generally consistent ($P > 0.05$).

A head-to-head pharmacokinetic comparison study was also conducted for the 700L scale-up product SCT510 versus Aflibercept ®. The crab-eating macaques were administered SCT510 and Aflibercept ® via a single intravenous dose at a dosage of 10mg/kg, with blood samples collected at different time points before and after administration, and the drug concentration was measured using the ELISA method. The results showed that under the experimental conditions, the main pharmacokinetic parameters of SCT510 and Aflibercept ® were basically consistent, with no significant differences ($P>0.05$). The C max and AUC (0-408h) were 289.40μg/mL and 292.24μg/mL, and 1.27day*mg/mL and 1.26day*mg/mL, respectively.

1.3.5 Preclinical toxicology comparative study

The non-clinical safety study of SCT510 was conducted using the crab-eating macaque as the relevant animal species, under GLP conditions, and included general pharmacology studies, long-term toxicity studies (including toxicokinetics), hemolytic and local irritation studies, tissue cross-reactivity, and immunogenicity / immunotoxicity studies, which were compared in parallel with Aflibercept®. The results showed that both had good overall tolerance in animals, with similar toxic reactions primarily manifested as long bone dysplasia; this toxic manifestation is related to the pharmacological effects of the antibodies and is consistent with reports on Aflibercept ®.

1.3.6 SCT510 I Phase clinical study data

From May 2018 to November 2018, a total of 84 participants were enrolled in the I phase clinical study of SCT510 and Bevacizumab in healthy male patients, during which 1 participant withdrew from the study prior to dosing. The pharmacokinetic curves of the two groups showed a generally similar trend, with no significant differences; there were no significant differences in the pharmacokinetic parameters between the two groups. The geometric mean ratio (GMR) of the primary PK parameter (AUC0-t) and the 90% CI was 0.8860 (0.8390, 0.9357), falling within the acceptable equivalence range (80.00% to 125.00%). The GMR and 90% CI for the secondary PK parameters (AUC 0-∞ and Cmax) were 0.8808 (0.8316, 0.9330) and 0.9745 (0.9153, 1.0376), respectively, both falling within the acceptable equivalence range (80.00% ~ 125.00%). This demonstrates that SCT510 is bioequivalent to the reference drug. Safety analysis showed that a total of 79 (95.2%) patients experienced TEAEs during the study, with 63 (75.9%) patients experiencing TEAEs related to the investigational drug; A total of 1 (1.2%) patients experienced sinus bradycardia classified as Grade 3 TEAE, and the adverse events of special interest (AESI) occurring during the study were proteinuria and elevated blood pressure, with 27 (32.5%) patients experiencing 33 incidents of AESI. No SAE, investigational drug-related SAE, deaths due to TEAE, TEAE leading to trial termination, TEAE leading to trial termination related to the investigational drug, TEAE leading to dose reduction, or TEAE leading to discontinuation of the investigational drug occurred during the study. The incidence of AE, TEAE, drug-related TEAE, grade 3 and above TEAE, and AESI in the SCT510 group and the Bevacizumab group was not statistically significant ($P > 0.05$). The Phase III clinical study on the efficacy and safety of SCT510 is currently ongoing and has not yet been unblinded.

2 Study Objectives

2.1 Phase II single-arm study

Primary Objective:

1) To evaluate the safety and tolerance of SCT-I10A combined with SCT510 in patients.

Secondary Objectives:

1) To evaluate the objective response rate (ORR), duration of response (DOR), disease control rate (DCR), progression-free survival (PFS), and overall survival (OS) of patients treated with SCT-I10A combined with SCT510 according to the RECIST v1.1criteria;
2) Evaluation of the pharmacokinetic characteristics of SCT-I10A and SCT510;
3) Evaluation of the immunogenicity of SCT-I10A and SCT510

2.2 Phase III randomized controlled trial

Primary Objective:

1) Evaluation of progression-free survival (PFS) and overall survival (OS) based on blinded independent central review (BICR) according to RECIST v1.1 for patients treated with SCT-I10A combined with SCT510 compared to Sorafenib.

Secondary Objectives:

1) One-year, 1.5-year, and 2-year overall survival rates (OSR) for patients treated with SCT-I10A combined with SCT510 compared to Sorafenib;
2) Investigators evaluated the progression-free survival (PFS) of patients treated with SCT-I10A combined with SCT510 compared to Sorafenib according to RECIST v1.1 criteria;
3) BICR and investigators evaluated the objective response rate (ORR), duration of response (DOR), and disease control rate (DCR) of SCT-I10A combined with SCT510 compared to Sorafenib in treating patients according to RECIST v1.1 criteria;
4) BICR evaluated the progression-free survival (PFS), objective response rate (ORR), duration of response (DOR), and disease control rate (DCR) of SCT-I10A combined with SCT510 compared to Sorafenib in treating patients according to mRECIST criteria;
5) Evaluate the safety of SCT-I10A combined with SCT510 in treating patients;

6) Evaluate the quality of life of patients treated with SCT-I10A combined with SCT510 compared to Sorafenib (EORTC QLQ-C30 and EORTC QLQ-HCC18 scales);
7) Evaluate the steady-state trough concentrations and pharmacokinetic characteristics of SCT-I10A and SCT510 in patients with advanced hepatocellular carcinoma;
8) Evaluate the immunogenicity of SCT-I10A and SCT510;
9) Evaluate the correlation between PD-L1 expression levels and efficacy, prognosis.

3 Study design

3.1 Principles of study design

This study is designed to assess the efficacy and safety of SCT-I10A combined with SCT510 as first-line treatment in patients with unresectable, recurrent, and/or metastatic hepatocellular carcinoma. The primary endpoints of the study are set as BICR evaluated progression-free survival (PFS) and overall survival (OS) as dual endpoints. Referencing previous data on Sorafenib in the Chinese population, PFS is approximately 3.5 months, and OS is 10-11.5 months. In the IMbrave150 study data for the Chinese population, the Atezolizumab combined with bevacizumab group had an mPFS of 5.7 months (HR=0.6), while the mOS in the experimental group has not yet been reached, with a HR=0.44. We assume that the control group has an mPFS of 3.6 months and an mOS of 12 months. It is expected that the SCT-I10A combined with SCT510 group will have an mPFS of 5.4 months (HR=0.67) and an mOS of 17.9 months (HR=0.67), indicating that SCT-I10A combined with SCT510 as first-line treatment for advanced hepatocellular carcinoma is superior to the current standard treatment with Sorafenib.

At the same time, this study has secondary efficacy endpoints including 1 year, 1.5 years, and 2 years overall survival rate (OSR), investigator-assessed progression-free survival (PFS), objective response rate (ORR), disease control rate (DCR), duration of response (DOR), and quality of life of patients (QoL) to demonstrate the therapeutic benefits of the investigational drug for the target patient population in China.

At the same time, tumor tissue from patients was collected during the screening period for PD-L1 immunohistochemical testing to evaluate the correlation between the biomarker PD-L1 and efficacy and prognosis.

3.2 Study design

3.2.1 Overall study design

This study is a multicenter, randomized, open-label Phase II/III clinical study to evaluate the efficacy and safety of SCT-I10A combined with SCT510 compared to Sorafenib as first-line treatment for advanced hepatocellular carcinoma, divided into Phase II single-arm study and Phase III randomized controlled trial 2 parts. The Phase II single-arm study part serves as a safety lead-in phase, primarily evaluating the safety and tolerance of SCT-I10A combined with SCT510 therapy, with a planned enrollment of 50-60 patients, receiving SCT-I10A 200mg d1 Q3W combined with SCT510 15mg/kg d1 Q3W therapy until disease progression, intolerable toxicity,

initiation of new antitumor treatment, decision to discontinue treatment after careful consideration by the patient or investigator, death, or loss to follow-up. After disease progression, if the investigator determines that continued treatment with SCT-I10A combined with SCT510 may provide clinical benefit, treatment with SCT-I10A combined with SCT510 may continue after obtaining patient consent, until the investigator determines that there is no clinical benefit, intolerable toxicity, the patient or investigator decides to terminate treatment after careful consideration, death, or loss to follow-up.

The final 1 patient in the Phase II study will undergo an analysis of the safety and efficacy of the Phase II single-arm study part before the 3 rd dose (after the first efficacy evaluation). Upon preliminary confirmation of safety and efficacy, the study will proceed to a III phase randomized, controlled trial.

The second part is a III phase randomized, controlled study, which plans to enroll approximately 342 patients, randomly assigned to the experimental group and control group in a 2: 1 ratio. The study includes 3 phases: screening phase, treatment phase, and follow-up phase.

3.2.1.1 Screening phase

After the patients sign the informed consent form, they will enter the study screening phase, during which the screening phase assessment will be completed according to the visit schedule in the study flowchart. The maximum duration of the screening phase is 28 days, and patients who meet all inclusion criteria and do not meet any exclusion criteria will be eligible to enter the study treatment phase.

3.2.1.2 Treatment period

Patients who meet the inclusion criteria will be randomly assigned (2:1) to the experimental group and the control group, with random stratification factors including ECOG (0 points vs 1 point), baseline AFP levels (< 400ng/ml vs ≥400ng/ml), presence or absence of major vascular invasion and / or extrahepatic metastasis (none vs present). The experimental group will receive the SCT-I10A combined with SCT510 regimen, while the control group will receive the Sorafenib regimen.

Experimental group: SCT-I10A: every 3 weeks (21 days) constitutes 1 cycle, with 200mg administered on day 1 of each cycle, via intravenous infusion, for a duration not exceeding 2 years.

SCT-510: Every 3 weeks (21 days) constitutes 1 cycle, with 15mg/kg administered on Day 1 of each cycle, via intravenous infusion, for a duration not exceeding 2 years.

Administration sequence: First, administer SCT-I10A, followed by SCT510. After the infusion of SCT-I10A is completed, flush the line and then administer SCT510. There should be an interval of more than 30 minutes between the end of SCT-I10A infusion and the start of SCT510 infusion.

If any drug is temporarily or permanently discontinued, other drugs may still be continued for treatment.

If any drug is delayed or interrupted due to adverse events during a treatment cycle, other investigational drugs that have not caused intolerable toxicity may continue to be administered during that cycle. If the investigational drug is delayed for more than 7 days, the administration of the drug for this cycle will be paused and resumed in the next cycle; If the investigational drug is delayed for less than or equal to 7 days, the drug can continue to be used in this cycle. For example, with SCT510: if the administration of SCT510 is delayed due to adverse events, SCT-I10A will continue to be administered as planned. If SCT510 is delayed for less than or equal to 7 days, treatment with SCT510 can continue in this cycle; if SCT510 is delayed for more than 7 days, the administration of SCT510 will be paused for this cycle and resumed in the next cycle. The delay time is calculated based on the first administration of the other investigational drug in this cycle.

Control group: Sorafenib: 3 weeks (21 days) constitutes 1 cycle, with each treatment cycle lasting daily 400mg in the morning and evening (with an interval of 12 hours as much as possible) taken on an empty stomach (1 hour before meals or 2 hours after meals) for 2 times, with a treatment duration not exceeding 2 years.

The control group received Sorafenib treatment until disease progression, intolerable toxicity, the initiation of new antitumor therapy, a decision to terminate treatment made after careful consideration by the patient or investigator, death, or loss to follow-up. After disease progression, if the investigator determines that continued treatment with Sorafenib may provide clinical benefit, it may continue with the patient's consent until the investigator determines that there is no longer clinical benefit, intolerable toxicity, a decision to terminate treatment made after careful consideration by the patient or investigator, death, or loss to follow-up.

The experimental group received SCT-I10A in combination with SCT510 until disease progression, intolerable toxicity, the initiation of new antitumor treatment, termination of treatment decided after careful consideration by the patient or investigator, death, or loss to follow-up. After disease progression, if the investigator determines that continued treatment with SCT-I10A combined with SCT510 may provide clinical benefit, treatment with SCT-I10A

combined with SCT510 may continue after obtaining patient consent, until the investigator determines that there is no clinical benefit, intolerable toxicity, the patient or investigator decides to terminate treatment after careful consideration, death, or loss to follow-up. Clinical benefit is the result of the investigator's comprehensive assessment based on the absence of intolerable toxicity or symptom deterioration due to disease progression, combined with imaging findings and clinical status.

During the screening period and throughout the study, tumor assessments will be determined by the investigator based on the Response Evaluation Criteria in Solid Tumors (RECIST) version 1.1 to decide whether patients should continue treatment. Baseline tumor assessment should be conducted within 28 days prior to the initiation of the investigational drug treatment. It is recommended that tumor assessment be performed using enhanced computed tomography (CT) or magnetic resonance imaging (MRI) (enhanced CT is preferred; if the patient is allergic to the CT contrast agent, a CT chest scan combined with MRI of other areas may be used). From the start of the first dose of the study treatment until 48 weeks, tumor efficacy evaluations should be conducted every 6 weeks (±7 days); after 48 weeks, evaluations should be conducted every 9 weeks (±7 days). The timing of efficacy evaluation visits is fixed, regardless of whether there is a delay in the treatment cycle. Until there is no clinical benefit, new anti-tumor treatment is initiated, informed consent is withdrawn, death occurs, or loss to follow-up, whichever occurs first. If symptoms indicative of PD occur, subsequent imaging methods need to be employed for assessment to record and confirm tumor efficacy.

From the start of the first dose of investigational treatment, safety assessments will be conducted every 2 cycles (±3 days). During the study treatment period, additional safety assessments are required: including physical examinations and vital signs before each SCT-I10A and SCT510 administration, before each cycle of Sorafenib treatment, as well as ECOG performance status, 12-ECG and basic laboratory tests (complete blood count, urinalysis, blood biochemistry). If the safety assessment time coincides with the administration time on the same day, it must be completed before the first dose of the investigational drug.

This study will also evaluate the pharmacokinetic characteristics of the patients.

This study will also assess the immunogenicity of the patients, specifically by detecting the presence of anti SCT-I10A antibodies and anti SCT510 antibodies before and after treatment.

3.2.1.3 Follow-up period

End of treatment visit (EOT): Patients who cannot clinically benefit from continued treatment due to disease progression, experience intolerable toxicity, or require termination of

investigational drug treatment for any other reason must undergo an end of treatment visit. All patients will be followed for adverse events. All adverse medical events should be collected from the time the patients receive the investigational drug until the last dose, 90 days later, or before the start of any new anti-tumor treatment, whichever occurs first. After this period, only severe adverse events related to the investigational drug or study procedures need to be collected. For AEs, follow-up must continue until any of the following conditions are met: the event resolves, the event stabilizes, the event returns to baseline levels, or the investigator reasonably determines (for example, if the event cannot be resolved or has improved) that further follow-up is unnecessary or that more information cannot be obtained (such as when the patient is lost to follow-up or refuses to provide further information).

Survival follow-up visits: After the end of treatment visit, patients will undergo follow-up for survival status. After the end of treatment visit, patients who terminate study treatment for reasons other than disease progression, initiation of new anti-tumor therapy, death, or loss to follow-up should continue to receive tumor assessments during this period until disease progression, initiation of new anti-tumor therapy, withdrawal of informed consent, death, or loss to follow-up, whichever occurs first. All patients who have received at least one dose of the investigational drug will undergo survival follow-up every 3 months (±14 days), which may be conducted via telephone inquiries, to collect information on new anti-tumor therapies initiated and survival status after the end of treatment visit, until death, loss to follow-up, or withdrawal of informed consent.

3.3 Risk / Benefit assessment

The overall survival (OS) for first-line treatment of patients with advanced liver cancer is between 6.4 and 13.6 months, with very limited options available. With the development of biological agents, targeted immunotherapy has shown better efficacy in hepatocellular carcinoma, but the effectiveness of monotherapy remains limited. Following the publication of the IMbrave150 study results, immunotherapy combined with anti-angiogenic therapy can provide greater survival benefits for patients. Compared to Sorafenib, the 'T+A' combination therapy reduced the risk of death (OS) by 42% (HR= 0.58; 95% CI: 0.42–0.79), reduced the risk of disease progression or death (PFS) by 41% (HR=0.59; 95% CI: 0.47–0.76), with adverse reactions mainly including hypertension, proteinuria, diarrhea, abdominal pain, and decreased appetite, indicating good patient tolerance. In addition to its anti-angiogenic properties, bevacizumab's immunomodulatory characteristics may also play a role in its clinical activity. We believe that the

two can form a good synergistic effect, and the safety of the combination of immunosuppressants and bevacizumab has been confirmed in multiple tumor types.

SCT-I10A is a humanized IgG4 monoclonal antibody that can specifically bind to PD-1 on the cell surface. By blocking the interaction between PD-1 and its ligands, it alters the tumor microenvironment, restores and enhances the immune killing function of T cells, thereby inhibiting tumor growth. Compared to currently marketed PD-1 monoclonal antibodies, its Phase I clinical trials did not reveal any unexpected adverse reactions, confirming that its safety is foreseeable and controllable. Preliminary efficacy data indicate that its anti-tumor activity is comparable to that of similar drugs.

SCT510 is a humanized IgG 1 monoclonal antibody that primarily acts by specifically binding to VEGF secreted by tumor cells, neutralizing its interaction with the surface receptor on vascular endothelial cells (VEGFR), thereby blocking the phosphorylation of intracellular tyrosine kinases of VEGFR and downstream signal transduction, which inhibits tumor angiogenesis and exerts an anti-tumor growth effect. Its antigen binding site and amino acid sequence are identical to the imported version of Bevacizumab (trade name Aflibercept®) available in our country. The preclinical toxicology studies are similar to those of Aflibercept ®, primarily showing that the test patients exhibited long bone developmental dysplasia, with no other unexpected adverse reactions, demonstrating a high degree of similarity in safety between the two. The Phase I clinical study conducted in healthy male patients on SCT510 and Bevacizumab showed that based on the analysis of the primary PK parameter AUC 0-t, SCT510 was proven to be bioequivalent to the reference drug, and the safety analysis indicated no statistical differences between the two groups.

In addition, a series of in vivo pharmacodynamic experiments were conducted for the combination treatment of SCT510 and SCT-I10A: since SCT510 does not bind to mouse VEGF, while another drug targeting the same site, SCT520F, binds to mouse VEGF, a comparative study of the pharmacodynamics of SCT510 and SCT520F was conducted using NCI-H460 nude mouse subcutaneous transplanted tumors, and no significant difference in efficacy was observed between the two. Based on the above conclusions, the drug targeting the same site as SCT510, 520F, is used as a substitute antibody to conduct a study on its combined antitumor efficacy with SCT-I10A. The experimental results indicate that neither SCT-I10A nor SCT520F monotherapy (15 mg/kg, every week 2 times, continuous administration 4 times) showed significant inhibitory effects on the growth of B16-F1 transplanted tumors, but their combination (15 mg/kg+15 mg/kg,

every week 2 times, continuous administration 4 times) exhibited significant tumor-suppressive effects.

Based on non-clinical and available clinical data, it is believed that SCT - I10A combined with SCT510 has anti-tumor synergistic effects, and its toxic reactions are controllable. Therefore, the combination may provide greater clinical benefits for patients with advanced hepatocellular carcinoma. More information on the known and anticipated benefits and risks of SCT - I10A / SCT510 can be found in the relevant sections of the investigator's brochure (IB).

In summary, any identifiable risks are considered to be monitorable and controllable.

The sponsor, monitors, and investigators will conduct this study in accordance with the study protocol, Good Clinical Practice (GCP), International Council for Harmonisation (ICH) guidelines, and applicable regulatory requirements.

3.4 Evaluation Criteria Indicators

3.4.1 Phase II single-arm study

Primary Study Endpoint:

1) Safety Assessment Endpoint:

- TEAE；
- Clinical laboratory test values;
- ECOG Performance Status Score;
- Vital signs;
- 12-ECG；
- Thyroid function;
- Physical examination, etc.;

Secondary Study Endpoint:

1) Efficacy Assessment Endpoint: The objective response rate (ORR), duration of response (DOR), disease control rate (DCR), progression-free survival (PFS), and overall survival (OS) of patients treated with SCT-I10A combined with SCT510 evaluated by the investigator according to RECIST v1.1 criteria;

2) PK evaluation: Pharmacokinetic evaluation indicators include but are not limited to: AUC0-t, AUC0-∞, Cmax, Cmin, CL, Vz, t1/2, etc.;

3) Immunogenicity assessment: Detection of anti-SCT-I10A antibodies and anti-SCT510 antibodies in the serum of patients before and after treatment in this study to evaluate immunogenicity.

3.4.2 Phase III randomized controlled trial

Primary Study Endpoint:

- Evaluation of the progression-free survival (PFS) and overall survival (OS) of patients treated with SCT-I10A combined with SCT510 compared to Sorafenib based on blinded independent central review (BICR) according to RECIST v1.1.

Secondary Study Endpoints

1) Other Efficacy Assessment Endpoints:

- 1-year, 1.5-year, and 2-year Survival Rates (OSR);
- The investigator evaluated the progression-free survival (PFS) of SCT-I10A combined with SCT510 compared to Sorafenib treatment in patients according to RECIST 1.1 criteria;
- BICR and the investigator evaluated the objective response rate (ORR), duration of response (DOR), and disease control rate (DCR) of SCT-I10A combined with SCT510 compared to Sorafenib treatment in patients according to RECIST 1.1 criteria;
- BICR evaluated the progression-free survival (PFS), objective response rate (ORR), duration of response (DOR), and disease control rate (DCR) of SCT-I10A combined with SCT510 compared to Sorafenib treatment in patients according to mRECIST criteria;
- Evaluate the changes in quality of life of patients in the experimental group compared to the control group (EORTC QLQ-C30 and EORTC QLQ-HCC18 scales);
- Evaluate the correlation between PD-L1 expression levels and efficacy, prognosis;

2) Safety assessment endpoints:

- TEAE；
- Clinical laboratory test values;

- ECOG Performance Status Score;
- Vital signs;
- 12-ECG；
- Thyroid function;
- Physical examination, etc.;

3) PK evaluation: Assess the steady-state trough concentrations and pharmacokinetic characteristics of SCT-I10A and SCT510 in patients with advanced hepatocellular carcinoma;

4) Immunogenicity assessment: Detect the levels of anti-SCT-I10A antibodies and anti-SCT510 antibodies in the serum of patients before and after treatment in this study to evaluate immunogenicity.

3.5 Randomization and Blinding

3.5.1 Randomization Method

This trial employs a block randomization method, with each center competing for enrollment, utilizing a central randomization system. Researchers at each participating center log into the randomization system after confirming each eligible patient, fill in the screening information, and obtain the random number and corresponding drug number information. The corresponding investigational drug is dispensed according to the random number and drug number, with each drug number assigned to only one patient and not reused.

The stratification factors for this trial include ECOG score (0 vs 1), baseline AFP level (< 400ng/ml vs ≥400ng/ml), presence of major vascular invasion and / or extrahepatic metastasis (none vs present), with a distribution ratio of 2:1 between the experimental group and the control group.

3.5.2 Blinding

This study is an open-label study and will not be blinded.

3.6 End of Study (EOS)

The cutoff date for the final analysis of overall survival (OS) will be after observing at least 250 deaths. A final analysis of the study data will be conducted, analyzing all data from all patients collected prior to this cutoff date, resulting in a clinical study report.

At the end of the study (EOS), patients who continue to receive study treatment due to ongoing CR, PR, or SD, or based on the investigator's judgment that the patient, despite disease progression, still has clinical benefit, may have the opportunity to enter an independent study. In this study, the sponsor will continue to provide the investigational treatment to patients who are deemed by the investigator to benefit from continued therapy, monitor safety, and report to health regulatory authorities as required by regulations. Before the current study concludes, the treatment protocol for this independent study will be submitted to health regulatory authorities and the IRB involved in the current study.

3.7 Trial termination

The trial center will be closed after all patients have completed treatment and assessment, and the data has been locked. In addition, the study may also be terminated at the request of the sponsor, investigator, or regulatory authorities. Any party requesting the termination of the study must promptly and accurately inform the other parties, and the trial center may be closed early after mutual consultation. If it is necessary to terminate or suspend the trial, the investigator should inform the ethics committee of the reasons for termination or suspension in accordance with relevant regulatory requirements. Upon termination of the study, the sponsor and the investigator will ensure that adequate consideration is given to protecting the rights of the patients.

The trial should be considered for termination if the following situations occur:

- The investigator discovers serious safety issues;
- The efficacy cannot meet expectations, and there is no need to continue the clinical trial;
- There are significant errors in the trial protocol;
- The regulatory authority withdraws the study;
- If the following situations occur, the sponsor has the right to decide to terminate the trial at a specific trial center:
  - The trial center is unable to complete the agreed enrollment number within the stipulated time;
  - The trial center has seriously violated GCP;
  - The trial center has seriously violated the protocol;
- It has been confirmed that the primary endpoint of the study has been reached;

After the termination of the study, all trial-related records should be retained for future reference.

Once the study is terminated, patients will be observed as soon as possible, and a treatment end visit assessment should be conducted. Researchers may notify patients of subsequent procedures aimed at ensuring adequate consideration for the protection of patients' interests. Researchers will be responsible for notifying IRBs and / or EC of the news regarding the early termination of the trial.

3.8 Closure of the study and research centers

At the end of the study, the monitor will collaborate with the researchers or personnel from the clinical trial site to carry out the following activities as deemed appropriate:

- Address all data queries;
- Count, verify, and arrange all unused study products;
- Review the consistency and completeness of study records;
- Transport biological samples to the analysis laboratory.

4 Study Population

4.1 Inclusion Criteria

Patients who meet all of the following criteria may be included in this study:

1. Voluntarily signed a written informed consent form prior to screening;
2. Age ≥ 18 years, regardless of gender;
3. ECOG performance status score of 0-1;
4. Clinically or pathologically diagnosed as hepatocellular carcinoma, the diagnosis of hepatocellular carcinoma must meet at least one of the following criteria:
    1) Histologically or cytologically confirmed as hepatocellular carcinoma, excluding fibrolamellar carcinoma, sarcomatoid carcinoma, or mixed cholangiocarcinoma-hepatocellular carcinoma;
    2) According to the 'Guidelines for Diagnosis and Treatment of Primary Liver Cancer (2019 Edition)', clinically diagnosed as HCC;
5. No prior systemic treatment for HCC (mainly including systemic chemotherapy, anti-angiogenic therapy, molecular targeted therapy, and immunotherapy involving PD-1, anti-PD-L1/L2, anti-CD137, or anti-CTLA-4 antibodies, or any other antibodies or drugs targeting T cell co-stimulation or immune checkpoint pathways) has been received, and disease progression is allowed for enrollment 6 months after the completion of postoperative adjuvant chemotherapy;
6. Barcelona Clinical Liver Cancer Staging (BCLC Staging) is Stage C or Stage B that is not suitable for surgery and/or local treatment;
7. Child-Pugh liver function classification: Class A and better Class B (≤7 points), with no history of hepatic encephalopathy;
8. According to the Response Evaluation Criteria in Solid Tumors (RECIST version 1.1), there must be at least one measurable lesion, and lesions that have shown clear disease progression after prior local treatment may also be selected as target lesions;
9. Expected survival of more than 3 months;
10. Major organ functions are normal (no blood components, cell growth factors, or albumin infusion treatments administered within 14 days prior to laboratory tests), meeting the following criteria:
    1) Blood routine: Neutrophils $\geq 1.5\times10^{9}$ /L, Platelets $\geq 75\times10^{9}$ /L, Hemoglobin ≥90g/L;
    2) Liver function: Alanine aminotransferase (ALT) and Aspartate aminotransferase (AST), ALT and AST ≤5×ULN; Serum albumin ≥28g/L; Total bilirubin (TBIL) ≤2×ULN;

Alkaline phosphatase (ALP) ≤5×ULN.

3) Kidney function: Serum creatinine (Cr) ≤1.5×ULN or Creatinine clearance rate (Ccr) ≥50mL/min;
4) Urinalysis shows urine protein <2 (+); If baseline urine protein ≥2 (+), 24-hour urine protein quantification must be ≤1.0g;
5) Coagulation function: Activated partial thromboplastin time (APTT), International normalized ratio (INR), Prothrombin time (PT) ≤1.5×ULN;
6) Cardiac echocardiogram: Left ventricular ejection fraction (LVEF) ≥50%;

4.2 Exclusion criteria

Patients meeting any of the following criteria should be excluded from this study:

1. Within 4 weeks prior to the first investigational drug treatment, patients who have undergone liver surgery and/or local treatment for HCC, except for palliative radiotherapy to relieve pain for bone metastatic lesions;
2. Patients who have not recovered from any toxicity and/or complications from local treatments (including interventional treatments, radiofrequency treatments, etc.), prior chemotherapy, surgery, or radiotherapy, i.e., have not decreased to ≤1 grade (NCI CTCAE version 5.0) (except for hair loss and fatigue);
3. Received antitumor drug treatment (including but not limited to traditional Chinese medicine preparations with antitumor indications) within 2 weeks prior to the first investigational drug treatment;
4. Moderate or severe ascites or ascites requiring drainage within 4 weeks prior to the first investigational drug treatment, or pleural effusion or pericardial effusion requiring drainage and/or accompanied by symptoms of dyspnea;
5. Patients with known active central nervous system metastases (CNS) and/or carcinomatous meningitis: Previously treated patients with brain metastases may participate in the study, provided they are clinically stable for at least 2 weeks, with no evidence of new or enlarged brain metastases, and have discontinued corticosteroids 14 days prior to the administration of the investigational drug. Stable brain metastases in this definition should be determined prior to the first administration of the investigational drug. Asymptomatic patients with brain metastases (i.e., no neurological symptoms, no need for corticosteroids, and no lesions >1.5 cm) may participate, but regular brain imaging should be conducted as part of disease monitoring;
6. Having other malignant tumors (excluding cured cervical carcinoma in situ, non-melanoma

skin cancer, or other tumors/cancers that have undergone radical treatment and have shown no signs of disease for at least 5 years);

7. Having a tendency to bleed, a high risk of bleeding, or coagulation disorders, including a history of venous or arterial thromboembolic events occurring within the 6 months prior to screening, such as myocardial infarction, unstable angina, cerebrovascular accidents or transient ischemic attacks, pulmonary embolism, deep vein thrombosis, or any other serious thromboembolic events (excluding those with implanted venous infusion ports or catheter-related thrombosis, or superficial vein thrombosis that stabilized after standard anticoagulation treatment); Any life-threatening bleeding events occurring within the 3 months prior to screening, including those requiring blood transfusion, surgery, or local treatment, or continuous medication treatment; Use of full-dose oral or parenteral anticoagulants, thrombolytics, or aspirin (> 325mg /day) or other medications that inhibit platelet function (such as clopidogrel, etc.) within 10 days prior to enrollment; Or having undergone surgical procedures, with lesions invading major blood vessels, as determined by the investigator to have a bleeding tendency;

Note: Preventive anticoagulation for maintaining venous access is allowed, provided that INR ≤ 1.5×ULN and APTT is within normal range within 14 days prior to enrollment;

8. Portal vein cancer thrombus involving the main trunk and left and right branches, or simultaneously involving the main trunk and the superior mesenteric vein. Or with inferior vena cava cancer thrombus or cardiac involvement;
9. Presence of unhealed wounds, active gastrointestinal ulcers or bleeding, fractures (excluding healed old fractures);
10. History of esophageal or gastric variceal rupture bleeding within 6 months prior to the first investigational drug treatment. Known severe varices on endoscopy within 3 months prior to enrollment. Evidence of portal hypertension, with a high risk of bleeding as assessed by the investigator;
11. A history of gastrointestinal perforation and/or fistula, abdominal abscess, or intestinal obstruction (including incomplete intestinal obstruction requiring parenteral nutrition) within 6 months prior to the first investigational drug treatment, extensive bowel resection (partial colectomy or extensive small bowel resection, complicated by chronic diarrhea), Crohn's disease, ulcerative colitis, or long-term chronic diarrhea;
12. Patients who have undergone major surgery within 4 weeks prior to enrollment or are expected to undergo major surgery during the study period (excluding diagnostic biopsy);

13. Active, known or suspected autoimmune diseases (such as systemic lupus erythematosus, rheumatoid arthritis, inflammatory bowel disease, autoimmune thyroid disease, multiple sclerosis, vasculitis, glomerulonephritis, etc.), a history of human immunodeficiency virus infection (HIV positive), or other acquired or congenital immunodeficiency diseases. However, the following patients are allowed to be enrolled: patients with type 1 diabetes whose condition is stable after using a fixed dose of insulin; Autoimmune hypothyroidism receiving stable doses of hormone replacement therapy; Skin diseases that do not require systemic treatment (such as eczema with rashes covering less than 10% of the body surface, psoriasis without ophthalmic symptoms, etc.);
14. History of liver or other organ transplantation or stem cell transplantation;
15. Known history of active tuberculosis;
16. Known history of interstitial lung disease, non-infectious pneumonia, or patients highly suspected of having interstitial lung disease; Patients with a history of drug-induced or radiation-induced non-infectious pneumonia who are asymptomatic are allowed to be enrolled;
17. Patients with active infections requiring systemic treatment or uncontrolled infections (excluding simple urinary tract infections or upper respiratory tract infections) within 14 days prior to the first investigational drug treatment, or those requiring treatment for syphilis infection;
18. Patients with severe medical conditions, such as grade III or higher cardiac dysfunction (New York Heart Association [NYHA]), ischemic heart disease (such as myocardial infarction or angina), or a history of myocardial infarction within the last 3 months that remains poorly controlled after medication treatment, diabetes (fasting blood glucose ≥10mmol/L) that remains poorly controlled after medication treatment, or hypertension (systolic blood pressure >140mmHg and/or diastolic blood pressure >90mmHg) that remains poorly controlled after medication treatment, as well as patients with a history of hypertensive crisis or hypertensive encephalopathy;
19. Patients with acute or chronic active hepatitis B or C virus infection, with hepatitis B virus (HBV) DNA > 2000 IU/ml or 10^4 copies/ml; Hepatitis C virus (HCV) RNA > 10^3 copies/ml; Positive for both hepatitis B surface antigen (HbsAg) and anti-HCV antibodies; Patients who have been treated with antiviral therapy and meet the above criteria, and are willing to continue antiviral treatment during the study, may be enrolled;
20. Exclusion of the following cases if immunosuppressive drugs are required within 2 weeks prior to enrollment or during the study;

1) Intranasal, inhaled, topical corticosteroids, or local corticosteroid injections (e.g., intra-articular injections);
2) Physiological doses of systemic corticosteroids (≤10mg/day of prednisone or equivalent dose);
3) Short-term (≤7 days) use of corticosteroids for the prevention or treatment of non-autoimmune allergic diseases;

21. Receiving live vaccines within 4 weeks prior to the first administration of the investigational drug or planning to receive live vaccines during the study (excluding live virus seasonal influenza vaccine);
22. Allergy to any component of the investigational drugs SCT-I10A, SCT510; or known allergy to any other monoclonal antibody; or allergy to any component of Sorafenib;
23. Pregnant or breastfeeding women, or patients planning to become pregnant during the treatment period and within 6 months after the end of treatment;
24. Patients of childbearing potential who are unwilling to use effective contraceptive measures (including male patients capable of causing pregnancy and female patients and their male partners) during the study period and for at least 6 months after the last administration of the investigational drug;
25. Patients currently enrolled in other investigational devices or investigational drug treatments, with a time since the last use of other investigational drugs or devices of less than or equal to 4 weeks;
26. It is known that the patients have a history of drug addiction or mental illness;
27. Any other diseases, metabolic disorders, or laboratory test abnormalities that the investigator believes make the patient unsuitable for investigational drug treatment, or that may affect the interpretation of the study results, or place the patient at high risk, or affect the collection of data and samples.

4.3 Early withdrawal from study / Treatment

4.3.1 Patient withdrawal from treatment

If any of the following situations occur during this study (determined to be related to SCT-I10A), the patient must withdraw from SCT-I10A investigational drug treatment, but further treatment with SCT510 is allowed:

- Grade 3 or 4 respiratory disease, or if the patient experiences grade 2 respiratory disease again;

- Grade 4 colitis/diarrhea;
- Grade 3-4 or recurrence of grade 2 or higher pancreatitis;
- Grade 2 or higher myocarditis;
- Grade 4 maculopapular rash/papules, grade 3-4 severe or life-threatening bullous diseases, including Stevens-Johnson syndrome (SJS) or toxic epidermal necrolysis (TEN);
- Grade 4 hepatitis (ALT or AST ≥ 20×ULN, or total bilirubin ≥ 10×ULN); for patients with grade 2 elevation of AST or ALT at baseline, if AST or ALT increases ≥ 50% compared to baseline during treatment and persists for at least 1 week, treatment should be discontinued;
- Grade 3 or 4 renal failure or nephritis;
- Grade 3 or 4 infusion-related reactions;
- Grade 4 pituitary inflammation;
- Grade 3 or more severe encephalitis/meningitis;
- Transverse myelitis of all grades;
- Grade 3 or more severe, recurrent Grade 2 motor and neurological disorders; Guillain-Barré syndrome of all grades;
- Grade 3 or more severe, or recurrent Grade 2 ocular disorders;
- Within 12 weeks, corticosteroids (or equivalent medications) cannot be reduced to 10 mg/day or less;
- Persistent grade 2 or 3 adverse reactions (excluding endocrine diseases controlled by hormone replacement therapy) that do not recover to grade 0-1 within 12 weeks after the last dose;
- Any grade 4 immune-related adverse reactions or recurrence of immune-related adverse reactions of grade 3 or higher;
- If the patient's efficacy evaluation is CR and they have received SCT-I10A treatment for at least 24 weeks, and have received the study treatment for ≥2 cycles after the first confirmation of efficacy as CR, consideration may be given to stopping the patient's SCT-I10A treatment;

- Adverse events (AEs) occur if the patient and/or investigator believe it is best or necessary to discontinue SCT-I10A treatment.

If any of the following situations occur during this study (determined to be related to SCT510), the patient must withdraw from SCT510 investigational drug treatment, but further treatment with SCT-I10A is allowed:

- Grade 3 or 4 bleeding events;
- Any grade of arterial thrombotic events;
- Grade 4 venous thromboembolism, including pulmonary embolism, or recurrent grade 3 venous thromboembolism after anticoagulation treatment.
- Stage 4 hypertension or stage 3 hypertension that remains poorly controlled after treatment;
- Gastrointestinal perforation;
- Wound dehiscence during treatment that requires intervention;
- Any grade 4 fistula, regardless of whether it was caused by SCT510;
- Any grade of fistula that is possibly or definitely caused by SCT510;
- Any grade of tracheoesophageal fistula;
- Reversible posterior leukoencephalopathy syndrome (PRES);
- At least grade 3 infusion-related reactions associated with SCT510 (excluding fever);
- Delay in the administration of SCT510 exceeding 6 weeks;
- AEs occur if the patient and/or investigator believe it is best or necessary to discontinue SCT510 treatment.

If any of the following situations occur during this study (judged to be related to Sorafenib), the patient must withdraw from Sorafenib treatment:

- Bleeding events of grade 2 or higher requiring medical intervention;
- Patients with grade 2 or higher myocardial ischemia and/or myocardial infarction;
- Grade 4 congestive heart failure;
- Grade 4 hypertension;
- Severe drug-induced liver injury;

- Gastrointestinal perforation;
- Grade 4 non-hematological toxicity;
- Situations requiring dose reduction after two dose reductions;
- Other researchers may decide to permanently discontinue treatment based on a risk/benefit assessment.

For patients who have received treatment with the investigational drug, if they withdraw from treatment early, follow-up visits as specified in the protocol (end-of-treatment visit and survival follow-up visit) should be completed as much as possible after obtaining their consent.

4.3.2 Patients may withdraw from the study on their own accord.

Patients may withdraw from the study at any time during the research process (including discontinuation of the investigational drug and related study assessments), and this will not affect their subsequent treatment. Researchers should strive to understand the reasons for patients withdrawing from the study and whether any adverse events occurred. Adverse events should be followed up.

5 Investigational drug

5.1 Basic information on the investigational drug

5.1.1 SCT-I10A

Drug name: Recombinant humanized anti-PD-1 monoclonal antibody injection

Drug source: Sinocelltech Ltd.

Drug composition: Each vial contains 4mL with 100mg recombinant humanized anti-PD-1 monoclonal antibody, 12.4mg histidine, 28.0mg sodium chloride, 33.6mg arginine hydrochloride, and 0.8mg polysorbate 80.

Characteristics: Colorless liquid, may have a slight milky appearance, essentially free of visible particles, and should not appear turbid.

Specification: 100mg (4mL)/bottle

Shelf life: Tentatively 24 months

Storage: Store at 2–8°C, protect from light, do not freeze. The prepared injection can remain stable for 24 hours at 2–8°C and can be stored for 6 hours at room temperature.

5.1.2 SCT510

Drug name: Recombinant humanized anti-VEGF monoclonal antibody injection

Drug source: Sinocelltech Ltd.

Drug composition: Each bottle contains 100mg of recombinant humanized anti-VEGF monoclonal antibody, 240mg of sucrose, 24.8mg of histidine, 16.8mg of arginine hydrochloride, and 1.6mg of Polysorbate 80 in 4mL.

Characteristics: Colorless liquid, slightly milky, essentially free of visible particles, and should not appear cloudy.

Specification: 100mg (4mL)/bottle

Shelf life: Tentatively 24 months

Storage: Store at 2–8°C, protect from light, do not freeze. The prepared injection solution can remain stable at 2–8°C for 24 hours and can be stably stored at room temperature for 12 hours.

5.2 Drug preparation and administration

5.2.1 SCT-I10A

Before administration, observe the characteristics of the drug; it is a colorless liquid with a slight milky appearance. If the solution is cloudy, discolored, or contains white, protein-like particles, it should be discarded. Do not shake the bottle. Under sterile conditions, withdraw the required volume of the drug and transfer it to the intravenous injection container. Dilute with 0.9% Sodium Chloride Injection or 5% Glucose Injection, with a final concentration range of 1~10mg/mL. Mix the diluted solution using a gentle inversion method; do not shake. Discard any unused vials or empty bottles.

Storage of injection: This product does not contain preservatives. The medication must be administered immediately after preparation. If it cannot be used immediately after preparation, the diluted injection should not be stored at room temperature for more than 6 hours, including the time in the container and the infusion time. Store at 2~8 ℃ (36 ℉-46 ℉) for no more than 24 hours. Do not freeze.

Administration: Administer intravenously over 60 minutes. After the infusion, flush the line to avoid mixing with other medications or intravenous drug solutions.

5.2.2 SCT510

Before administration, observe the characteristics of the drug; it is a colorless liquid with a slight milky appearance. If the solution is cloudy, discolored, or contains white, protein-like particles, it should be discarded. Do not shake the bottle. Under sterile conditions, withdraw the required volume of the drug and transfer it to the intravenous injection container. Dilute with 0.9% Sodium Chloride Injection, with a final concentration range of 1.4~16.5mg/mL. Mix the diluted solution gently by inversion; do not shake. Discard any unused vials or empty bottles.

Storage of injection: This product does not contain preservatives. The medication must be administered immediately after preparation; if it cannot be used right away, the diluted injection can be stored at room temperature for up to 12 hours, including the time in the container and the infusion time. Store at 2~8 ℃ (36 ℉-46 ℉) for no more than 24 hours. Do not freeze.

Administration: The first intravenous infusion time should last 90 minutes (±15min). If the tolerance for the first infusion is good, the time for the second infusion can be shortened to 60 minutes (±15min). If the patients also have good tolerance for the 60 minute infusion, then all subsequent infusions can be completed in 30 minutes (±15min).

5.2.3 Sorafenib

400mg per dose, twice daily, morning and evening (with an interval of approximately 12 hours), on an empty stomach (1 hour before meals or 2 hours after meals), taken with a glass of warm water. Details regarding the specific administration and storage of Sorafenib should follow local guidelines based on treatment standards and the product's instructions.

5.3 Adjustments to investigational drug treatment

In combination therapy, every adverse event should be attributed as much as possible to the individual SCT-I10A or SCT510 or the combination treatment, and the dosage should be adjusted or treatment interrupted / terminated according to the dosage adjustment principles for each drug.

5.3.1 SCT-I10A Treatment adjustments

Reducing the dosage of SCT-I10A is not permitted. Treatment with SCT-I10A may be interrupted or stopped due to toxicity. Adverse events (both non-serious and serious) associated with exposure to SCT-I10A may represent an immune-related etiology. These adverse events may occur shortly after the first dose or within a few months after the last dose.

Treatment adjustments for SCT-I10A in the event of immune-related toxicity can refer to the 2019 CSCO guidelines for the management of toxicities related to immune checkpoint inhibitors, with Table 4 listing the recommended treatment adjustments for SCT-I10A-related toxicities. These specific guidelines apply to situations causally related to SCT-I10A.Table4

Table4 Management of immune-related adverse events

Related to the investigational drug Adverse reactions	Level of treatment interruption (CTCAE grading)	Time to restart treatment	Permanent discontinuation
Respiratory system diseases (e.g., interstitial pneumonia)	Grade 2	Toxicity reduced to Grade 0-1	Toxicity not resolved within 12 weeks after the last dose, or unable to reduce the corticosteroid dosage to 10 mg/day or lower (or equivalent medication) within 12 weeks
	Grade 3-4	Permanent discontinuation	Permanent discontinuation
Elevated AST, ALT, or bilirubin [a]	Grade 2-3	Toxicity reduced to grade 0-1	Toxicity not resolved within 12 weeks after the

Related to the investigational drug Adverse reactions	Level of treatment interruption (CTCAE grading)	Time to restart treatment	Permanent discontinuation
			last dose, or unable to reduce the corticosteroid dosage to 10 mg/day or lower (or equivalent medication) within 12 weeks
	Grade 4	Permanent discontinuation	Permanent discontinuation
Renal failure or nephritis	Grade 2	Toxicity reduced to grade 0-1	Toxicity not resolved within 12 weeks after the last dose, or unable to reduce the corticosteroid dosage to 10 mg/day or lower (or equivalent medication) within 12 weeks
	Grade 3-4	Permanent discontinuation	Permanent discontinuation
Colitis/diarrhea	Grade 2-3	Toxicity reduced to grade 0-1	Toxicity not resolved within 12 weeks after the last dose, or unable to reduce the corticosteroid dosage to 10 mg/day or lower (or equivalent medication) within 12 weeks
	Grade 4	Permanent discontinuation	Permanent discontinuation
Pancreatitis	Grade 2	Toxicity reduced to grade 0-1	Toxicity not resolved within 12 weeks after the last dose, or unable to reduce the corticosteroid dosage to 10 mg/day or lower (or equivalent medication) within 12 weeks
	Grade 3-4	Permanent discontinuation	Permanent discontinuation

**Related to the investigational drug Adverse reactions**	**Level of treatment interruption (CTCAE grading)**	**Time to restart treatment**	**Permanent discontinuation**
Myocarditis	Grade 2 or above	Permanent discontinuation	Permanent discontinuation
Endocrine dysfunction	Grade 2-3 pituitary inflammation	Toxicity reduced to grade 0-1	Toxicity not resolved within 12 weeks after the last dose, or unable to reduce the corticosteroid dosage to 10 mg/day or lower (or equivalent medication) within 12 weeks
	Grade 4 pituitary inflammation	Permanent discontinuation	Permanent discontinuation
	Grade 3 or above hyperglycemia (fasting blood glucose > 13.9-27.8 mmol/L)	Suspend treatment and insulin replacement therapy may be used until blood glucose levels return to grade 2 or below and remain stable	
	Symptomatic hypothyroidism<br>Symptomatic hyperthyroidism, or asymptomatic hyperthyroidism with TSH < 0.1 mU/L	Continue treatment and may use relevant symptomatic management or alternative therapies	
	Adrenal cortical insufficiency of grade 2 or above	Toxicity reduced to grade 0-1	Toxicity not resolved within 12 weeks after the last dose, or unable to reduce the corticosteroid dosage to 10 mg/day or lower (or equivalent medication) within 12 weeks
Encephalitis/Meningitis	Grade 1-2	Toxicity recovered to grade 0	Toxicity not resolved within 12 weeks after the last dose, or unable to reduce the corticosteroid dosage to 10 mg/day or lower (or equivalent medication) within 12 weeks

**Related to the investigational drug Adverse reactions**	**Level of treatment interruption (CTCAE grading)**	**Time to restart treatment**	**Permanent discontinuation**
	Grade 3-4	Permanent discontinuation	Permanent discontinuation
Transverse myelitis	All grades	Permanent discontinuation	Permanent discontinuation
Movement and neurological disorders	Grade 2	Toxicity reduced to grade 0-1	Toxicity not resolved within 12 weeks after the last dose, or unable to reduce the corticosteroid dosage to 10 mg/day or lower (or equivalent medication) within 12 weeks
	Grade 3-4	Permanent discontinuation	Permanent discontinuation
	Guillain-Barré syndrome of all grades	Permanent discontinuation	Permanent discontinuation
Skin toxicity	Grade 2-3 maculopapular rash	Toxicity reduced to grade 0-1	Toxicity not resolved within 12 weeks after the last dose, or unable to reduce the corticosteroid dosage to 10 mg/day or lower (or equivalent medication) within 12 weeks
	Grade 4 maculopapular rash	Permanent discontinuation	Permanent discontinuation
	Grade 3 pruritus	Toxicity reduced to grade 0-1	Toxicity not resolved within 12 weeks after the last dose, or unable to reduce the corticosteroid dosage to 10 mg/day or lower (or equivalent medication) within 12 weeks
	Grade 1-2 bullous dermatitis	Toxicity reduced to grade 0	Toxicity not resolved within 12 weeks after the last dose, or unable to reduce the corticosteroid dosage to 10 mg/day or

Related to the investigational drug Adverse reactions	Level of treatment interruption (CTCAE grading)	Time to restart treatment	Permanent discontinuation
			lower (or equivalent medication) within 12 weeks
	Grade 3-4 bullous dermatitis	Permanent discontinuation	Permanent discontinuation
Ocular diseases	Grade 2	Toxicity reduced to grade 0-1	Toxicity not resolved within 12 weeks after the last dose, or unable to reduce the corticosteroid dosage to 10 mg/day or lower (or equivalent medication) within 12 weeks
	Grade 3-4	Permanent discontinuation	Permanent discontinuation
Other immune-related adverse reactions (please refer to the notes below [b])	Grade 2-3	Toxicity reduced to grade 0-1	Toxicity not resolved within 12 weeks after the last dose, or unable to reduce the corticosteroid dosage to 10 mg/day or lower (or equivalent medication) within 12 weeks
	Grade 4	Permanent discontinuation	Permanent discontinuation

Note: If any severe or ≥ Grade 3 (pneumonia ≥ Grade 2) drug-related adverse events occur again, or any life-threatening adverse events, treatment should be permanently discontinued.

a For patients with a baseline AST or ALT elevation of Grade 2, if ALT or AST increases by ≥50% compared to baseline during treatment and persists for at least 1 week, treatment should be permanently discontinued.

b For patients experiencing intolerable related adverse events, the investigator may consider pausing SCT-I10A at their discretion.

Like all monoclonal antibody treatments, SCT-I10A also carries the risk of allergic reactions, including anaphylactic shock. SCT-I10A should be administered in an environment where immediate transfer to an intensive care unit or similar setting is possible, and where anti-allergic treatment (such as resuscitation measures) can be promptly initiated. Immediate access to steroids (dexamethasone 8-10mg), epinephrine (dilution of 1:1000), anti-allergic medications (IV antihistamines), bronchodilators or equivalent medications, and oxygen therapy should be ensured.

The subsequent SCT-I10A administration before whether pre-medication is needed is determined based on clinical judgment and the presence or severity of infusion reactions.

During the infusion of SCT-I10A, close monitoring of infusion-related reactions is required, and potential infusion-related reactions should be observed for 30 minutes after the infusion.

Infusion-related reactions may occur with the use of SCT-I10A, with symptoms potentially including fever, chills, rigidity, sweating, and headache.

The treatment of infusion-related reactions and adjustments to the infusion rate and / or treatment interruption / or cessation primarily depend on the severity, as shown in TableTable 5 Treatment Adjustments for SCT-I10A Infusion-Related Reactions5

Table 5 Treatment Adjustments for SCT-I10A Infusion-Related Reactions5

NCI CTCAE Grading	Treatment Adjustments
Grade 1<br>Definition: Mild, transient reactions; no need to interrupt infusion; no treatment required.	Reduce the infusion rate of SCT-I10A by 50% and closely monitor for worsening conditions.
Grade 2<br>Definition: Requires treatment or interruption of infusion, but symptomatic treatment (antihistamines, NSAIDs, analgesics, infusion therapy) is effective quickly; preventive medication ≤ 24 hours.	Stop the SCT-I10A infusion. Once the infusion-related reaction resolves or the severity decreases to at least Grade 1, continue the infusion at 50% of the previous rate, closely monitoring for recurrence or worsening.
Grade 3<br>Definition: Symptom relief delay (e.g., symptomatic treatment and/or interruption of infusion, inability to respond quickly); recurrence after symptom improvement; need for hospitalization due to sequelae.	Immediately stop the infusion of SCT-I10A.<br>The experimental drug treatment must be permanently discontinued.
Grade 4<br>Definition: Life-threatening; requires emergency treatment.	

Note: NSAID = Non-Steroidal Anti-Inflammatory Drug
If the infusion rate of SCT-I10A is reduced by 50% due to infusion reactions, this reduced rate must be maintained in the next scheduled infusion. If no infusion reactions are observed during the next planned infusion, the infusion rate can be restored to the baseline infusion rate during subsequent infusions.

5.3.2 SCT510 Treatment adjustment

It is not recommended to reduce the SCT510 usage dosage; the actual infusion dosage of SCT510 should not deviate from the planned dosage ±5%.

If the patient's weight changes compared to the weight reference value corresponding to the current dosage is < 10 %, no dosage adjustment of SCT510 is necessary; if the weight change is ≥ 10 %, the dosage needs to be recalculated;

When the following special adverse events of concern occur, the recommended measures for the investigational drug are as follows (the following grading is based on CTCAE5.0 version):

(1) Hemorrhage

Patients who experience grade 3 or grade 4 hemorrhagic events should receive appropriate treatment and permanently discontinue the injection of recombinant humanized anti-VEGF monoclonal antibody (SCT510).

(2) Thrombosis/Embolism

Patients who experience any grade of arterial thrombotic events should permanently discontinue the injection of recombinant humanized anti-VEGF monoclonal antibody (SCT510).

Patients who experience grade 4 venous thrombosis, including pulmonary embolism, should permanently discontinue the injection of recombinant humanized anti-VEGF monoclonal antibody (SCT510).

The patient developed Grade 3 venous thrombosis, and treatment with SCT510 was suspended. If the planned anticoagulation treatment duration is <2 weeks, suspend the investigational drug treatment until the anticoagulation treatment is completed. If the planned anticoagulation treatment duration is ≥2 weeks, suspend SCT510 treatment for 2 weeks; if the following criteria are met after 2 weeks, the investigational drug treatment may be resumed during the anticoagulation treatment:

- Before resuming investigational drug treatment, the INR should be within the target range (typically between 2-3);
- No bleeding events were found in the patients during previous tumor evaluations;
- No evidence of tumor invasion or proximity to major blood vessels was found in the patients during previous tumor evaluations.

Note: The anticoagulation treatment for the therapeutic dosage is defined as gradually increasing the dosage of anticoagulant drugs from a small amount to reach a maintenance INR level not lower than 1. 5 (usually between 2-3). The dosage of anticoagulant drugs should be recorded in the EDC, and patients receiving anticoagulation treatment should have their INR monitored throughout the treatment period.

If the patient experiences a grade 3 venous thrombosis again after anticoagulation treatment, then treatment with SCT510 will be permanently discontinued.

(3) Hypertension

- Grade 1 hypertension: Prehypertension (systolic blood pressure 120 - 139 mmHg or diastolic blood pressure 80 - 89mmHg). Continue treatment with SCT510.

- Grade 2 hypertension: systolic blood pressure 140-159 mmHg, or diastolic blood pressure 90-99 mmHg, requires medical intervention if it is recurrent or persistent (≥24 hours), symptomatic diastolic blood pressure elevation >20 mmHg, or if previously within normal range but this measurement shows systolic blood pressure >140 mmHg or diastolic blood pressure >90 mmHg, single-agent treatment should be administered. Once blood pressure is restored to Grade 1 or below, patients may continue to receive SCT510 treatment.

- Grade 3 hypertension: systolic blood pressure ≥160 mmHg, or diastolic blood pressure ≥100 mmHg, requires medical intervention, necessitating one or more antihypertensive medications or a higher intensity of treatment than previously. SCT510 treatment should be suspended until hypertension returns to grade 1 or below. If blood pressure remains poorly controlled after treatment, SCT510 treatment should be permanently discontinued.

- Grade 4 hypertension: life-threatening (e.g., hypertensive crisis, transient or persistent neurological deficits, malignant hypertension), requires emergency treatment. If grade 4 hypertension occurs, treatment with SCT510 should be permanently discontinued.

(4) Proteinuria

All patients should undergo urine protein testing within 3 days prior to each administration of the recombinant humanized anti-VEGF monoclonal antibody injection (SCT510).

Dose adjustments for the recombinant humanized anti-VEGF monoclonal antibody injection (SCT510) after the occurrence of proteinuria should be carried out as follows.

<u>First occurrence of proteinuria:</u>

- < 2+ (urinalysis): Continue SCT510 administration as planned, no additional testing is required.

- ≥2+ (urinalysis): Continue SCT510 administration as planned, and measure 24-hour urinary protein within 3 days before the next cycle. Adjust the dosage as follows based on the test results:

A. 24-hour urinary protein ≤2 grams: Continue SCT510 administration as planned.

B. If the 24-hour urinary protein exceeds 2 grams: suspend the current plan for SCT510 administration, and test the 24-hour urinary protein within 3 days prior to the next planned administration. Delay SCT510 administration until the 24-hour urinary protein is ≤2 grams. Test the 24-hour urinary protein before each subsequent SCT510 administration until it decreases to ≤1 gram/24 hours. Suspend SCT510 only if it exceeds 2 grams.

<u>Proteinuria reappearing for the second time and thereafter:</u>

- < 3+ (urinalysis): Continue SCT510 administration as planned, no additional tests are required.
- ≥3+ (urinalysis): Continue SCT510 administration as planned, and test 24-hour urine protein within 3 days before the next treatment cycle. Adjust the dosage based on the test results as follows:

A. 24-hour urinary protein ≤2 grams: Continue SCT510 administration as planned.

B. If the 24-hour urinary protein exceeds 2 grams: suspend the current plan for SCT510 administration, and test the 24-hour urinary protein within 3 days prior to the next planned administration. Delay SCT510 administration until the 24-hour urinary protein is ≤2 grams. Test 24-hour urine protein before each subsequent SCT510 administration until it decreases to ≤1 gram/24 hours. Only suspend SCT510 administration if it exceeds 2 grams.

(5) Gastrointestinal perforation

If gastrointestinal perforation occurs, appropriate treatment measures should be taken, and SCT510 therapy should be permanently discontinued.

(6) Surgical and wound healing complications

If patients experience surgical and wound healing complications during treatment, SCT510 therapy should be suspended until the wound is completely healed. If any elective surgical procedures are required, SCT510 therapy should be suspended first.

If a wound opens during treatment and requires intervention, then SCT510 should be permanently discontinued.

(7) Fistula

Patients must permanently discontinue SCT510 treatment if any of the following situations occur:

- Any Grade 4 fistula, regardless of whether it is caused by SCT510;
- Any grade of fistula that is possibly or definitely caused by SCT510;
- Any grade of tracheoesophageal fistula.

If a patient develops any other fistula not mentioned above, and the treating physician determines that the occurrence of the fistula is unrelated to SCT510 but related to surgery, then SCT510 treatment should be suspended, and conservative or surgical treatment should be provided until the fistula is completely healed.

If a patient develops any other fistula not mentioned above, and the treating physician determines that the occurrence of the fistula is unrelated to SCT510 but related to the disease itself, then SCT510 treatment should be suspended, and conservative or surgical treatment should be provided until the fistula is completely healed.

(8) Reversible posterior leukoencephalopathy syndrome (PRES)

Reports of patients developing symptoms / signs of reversible posterior leukoencephalopathy syndrome (PRES) following treatment with Bevacizumab are extremely rare. PRES is a rare neurological disorder characterized by seizures, headaches, altered mental status, visual disturbances, or cortical blindness, with or without hypertension. The diagnosis of PRES requires confirmation by brain imaging results, with magnetic resonance imaging being the preferred method. In patients who experienced PRES, it is recommended to adopt specific symptomatic treatment including controlling hypertension, while discontinuing SCT510 therapy.

(9) Infusion-related reactions

During medication, if an acute infusion reaction occurs, the recommended management methods are detailed in Table 6, and the final decision is made by the investigator based on clinical experience.Table 6 Management of Acute Infusion Reactions6

Table 6 Management of Acute Infusion Reactions6

Severity of Symptoms	Recommended Interventions
Mild-Moderate<br>For example, skin reactions graded ≤2 according to CTCAEv5.0, dyspnea, tachycardia, hypotension, headache, myalgia, edema, nausea.	• Suspend the use of SCT510 infusion;<br>• Administer 50 mg of diphenhydramine and/or 10 mg of dexamethasone intravenously;<br>• After complete recovery from the infusion reaction, the investigator may re-administer SCT510 based on the patient's condition and benefits;

Severity of Symptoms	Recommended Interventions
	• If an acute infusion reaction occurs again, the investigator will determine whether SCT510 should be permanently discontinued.
Severe<br>Such as systemic bronchospasm, generalized urticaria,<br>angioedema, allergic reactions.	• Permanently discontinue SCT510 infusion;<br>• Administer 50 mg of diphenhydramine and/or 10 mg of dexamethasone intravenously and/or administer epinephrine if necessary.

When other 3 grade or 4 grade SCT510 related toxic reactions (according to CTCAEv5.0) occur, apart from the above-mentioned special adverse events of concern, the following criteria should be used to determine whether to continue or discontinue the use of SCT510:

Upon first occurrence:

- Suspend the use of SCT510 until the symptoms of the toxic reaction return to baseline levels or at least the symptoms are reduced to 1 grade or below.

When it occurs again after resuming the investigational drug:

- If a 3 grade toxic reaction related to the use of SCT510 occurs again, the investigator should assess the risk/benefit of continuing the investigational drug for the patients;
- If a grade 4 toxicity reaction related to the use of SCT510 occurs again, the use of SCT510 should be permanently discontinued.

5.3.3 Adjustment of Sorafenib Treatment

Management of suspected adverse reactions includes pausing or reducing the dose of Sorafenib. If a dose reduction is necessary, the Sorafenib dose can be reduced to 400mg once daily. If further dose reduction is needed, it can be decreased to 400mg every other day, allowing for a reduction 2 times. If further reduction is still required, Sorafenib treatment should be discontinued. TableTable7 lists the recommended dose adjustments when patients experience skin toxicity.

Table7 Recommended dosage adjustments when skin toxicity occurs

Skin adverse reaction grading	Frequency of adverse reactions	Recommended dosage adjustment
Grade 1: Numbness, sensory	Occurs at any time	Continue using this product while

dullness, abnormal sensations, tingling, painless swelling, hand-foot erythema or discomfort that does not affect daily activities		providing local treatment to alleviate symptoms.
Grade 2: Hand-foot erythema and swelling with pain, and/or hand-foot discomfort affecting daily life	First occurrence	Continue using this product while providing local treatment to alleviate symptoms. If symptoms do not improve within 7 days, see below
	Symptoms have not improved within 7 days or the<br>2nd or 3rd occurrence	Interrupt treatment with this product until toxicity resolves to grade 0-1. When treatment with this product is resumed, reduce the dosage level by one.
	4th occurrence	Terminate treatment with this product.
Grade 3: Moist desquamation, ulcers, hand-foot blisters, pain, or severe hand-foot discomfort that prevents the patient from working and normal living.	1st occurrence or 2nd occurrence	Interrupt treatment with this product until toxicity resolves to grade 0-1. When treatment with this product is resumed, reduce the dosage level by one.
	3rd occurrence	Terminate treatment with this product.

For patients who require a dosage reduction due to grade 2 or 3 skin toxicity, if skin toxicity improves to grade 0-1 at least 28 days after reducing the dosage of Sorafenib, the dosage of Sorafenib may be increased by one level.

Patients requiring major surgery are advised to suspend Sorafenib. Clinical experience regarding when to resume Sorafenib after surgery is limited; therefore, it is decided that clinical considerations should be made before the patient resumes treatment to ensure wound healing. For cases requiring suspension or permanent discontinuation of Sorafenib, please refer to the table below:

Table8 Recommended dosage adjustments for Sorafenib when other adverse reactions occur

**Adverse reactions**	**CTCAE Grade**	**Management**	**Dosage reduction and resumption**
Cardiovascular events			
Myocardial ischemia and/or myocardial infarction	Grade 2 and above	Permanent discontinuation	No resumption
Congestive heart failure	Grade 3	Suspend treatment until recovery to ≤ Grade 1 [a]	Reduce by one dose level

	Grade 4	Permanent discontinuation	No resumption
Bleeding events requiring medical intervention	Grade 2 and above	Permanent discontinuation	No resumption
Hypertension	Grade 2 asymptomatic with diastolic pressure at 90-99 mmHg	Antihypertensive treatment	Continue medication as planned and closely monitor blood pressure
	Grade 2 (symptomatic/persistent) or Grade 2 symptomatic diastolic increase of 20 mmHg or baseline blood pressure within normal range rising to >140/90 mmHg or Grade 3	Suspend medication until symptoms resolve and diastolic pressure <90 mmHg	For antihypertensive drug treatment, reduce the dosage level by one step when resuming medication; if necessary, it can be further reduced by one dosage level.
	Grade 4	Permanent discontinuation	No resumption
Gastrointestinal perforation	Any grade	Permanent discontinuation	No resumption
QT interval prolongation	If QTc > 500 milliseconds or an increase of 60 milliseconds or more from baseline, monitor electrolytes and ECG.	Suspend medication and correct electrolyte abnormalities (magnesium, potassium, calcium).	Decide whether to restart treatment based on medical judgment.
Severe drug-induced liver injury	ALT elevation > grade 3 without other causes [b] ;<br>AST/ALT > 3 xULN and bilirubin > 2 xULN without other causes [b]	Permanent discontinuation	No resumption
Non-hematological toxicity	Grade 2	Timely intervention	Reduce by one dose level
	Grade 3		
	First occurrence	Suspend medication until it decreases to ≤ Grade 2	Reduce by one dose level
	No improvement within 7 days or occurrence of the 2nd or 3rd time	Suspend medication until it decreases to ≤ Grade 2	Reduce by two dosage levels

	Grade 4	Permanent discontinuation	No resumption

a . If recovery does not occur within 30 days after medication suspension, permanently discontinue unless the patient can clinically benefit.

b . Additionally, exclude any level of alkaline phosphatase elevation and ≥ Grade 2 bilirubin elevation caused by bone lesions; Meet any of the following conditions: exclude INR ≥ 1.5 due to potential liver cirrhosis or other organ failure; ascites and/or encephalopathy are considered drug-induced liver injury.

5.3.4 Other medications

Preventive medications will be dispensed at each trial center through the hospital pharmacy. Investigators should administer the medication according to the dosage and administration guidelines, safety issues (warnings, precautions), adverse reactions, dosage adjustments, and discontinuation information provided in the product insert. Investigators should also adhere to any relevant local practices when administering these pre-treatments. The sponsor will not bear the costs of these preventive treatment medications.

5.4 Termination of study treatment after CR

If the patient's efficacy evaluation is CR and they have received SCT-I10A and / or SCT510 treatment for at least 24 weeks, while also having received SCT-I10A and / or SCT510 treatment for ≥2 cycles after the first confirmation of efficacy as CR, consideration may be given to stopping SCT-I10A and / or SCT510 treatment.

5.5 Combination therapy / Treatment

All medications administered from the signing of the informed consent form until 90 days after the last dose or before the start of new antitumor therapy (whichever occurs first) will be recorded in the EDC. Changes in medication dosage or adjustments will also be documented.

Investigators may provide standard treatment and palliative care for existing comorbidities, medical / or surgical complications: administering sedatives, antiemetics, antibiotics, analgesics, antihistamines, steroids, granulocyte colony-stimulating factors, as well as red blood cells, erythropoietin, platelets, or fresh frozen plasma transfusion products to help manage pain, infections, and other complications of malignant tumors. If febrile neutropenia or evidence of infection occurs, intravenous antibiotic treatment may be administered.

For any immune-related AE that arises, active treatment should be undertaken, and consultation with relevant specialty physicians should be sought if necessary. For the management of immune-related toxicities, refer to the 2019 CSCO guidelines on toxicity management related to immune checkpoint inhibitors.

During the trial treatment period, local radiotherapy may be implemented for pain relief or for osteolytic lesions at risk of fracture, but prior to radiotherapy, it should first be confirmed whether the patient has experienced PD according to RECIST version 1.1. If a patient undergoes palliative radiotherapy after the start of the study treatment, the reason for the treatment must be clearly documented, and the target and non-target lesions assessed according to RECIST version 1.1. If radiation has been received during the trial, it must not be further used for response assessment.

If the patient has already started bisphosphonate treatment before the initiation of the study treatment, the use of bisphosphonates is allowed, regardless of the indication. If there is a consideration to increase the ongoing bisphosphonate treatment dose or to initiate bisphosphonate treatment due to worsening bone pain, the patient must first be confirmed for PD according to RECIST version 1.1, unless disease progression is ruled out and clearly documented in the patient's source documents; otherwise, patients who need to start bisphosphonate treatment during the study will be assessed as having disease progression.

Systemic corticosteroids required to control infusion reactions or immune-related adverse events must be gradually tapered over at least one month and should not be used at immunosuppressive doses (equivalent to ≤10mg/day of prednisone) prior to the next administration of the investigational drug. For patients with a known allergy to diagnostic imaging contrast agents, the administration of steroids as a preventive treatment is permitted.

Patients may continue hormone replacement therapy if it was already being used prior to enrollment.

Investigators may decide to administer any medication that is necessary for the health of the patients and will not interfere with the investigational drug.

5.6 Prohibited drugs / Treatment

During the treatment period of this study, no additional anti-tumor therapeutic agents may be administered to patients, including but not limited to systemic immunotherapy, chemotherapy, radiotherapy, biological therapy (including cytokines), immunosuppressants, or any other investigational drugs, as well as any traditional Chinese medicine formulations approved for anti-tumor treatment (those labeled with anti-cancer or anti-tumor in the traditional Chinese medicine instructions are not allowed). If necessary, the investigator may decide to administer traditional Chinese medicine for non-anti-tumor treatment indications, such as supportive therapy.

Before the first administration of the study treatment 28 days until the end of treatment, patients must not receive live vaccines, including but not limited to: measles, mumps, rubella, varicella, yellow fever, seasonal influenza, H1N1 influenza, rabies, BCG and typhoid. Inactivated influenza vaccines may be used.

Patients receiving treatment with Sorafenib should avoid using CYP3A4 inducers during the study treatment period: including but not limited to rifampicin; St. John's wort (Hypericum perforatum); phenytoin; carbamazepine; phenobarbital; and dexamethasone.

5.7 Management of investigational drugs

The distribution and collection of investigational drugs are the responsibility of designated personnel. Researchers must ensure that all investigational drugs are used solely for patients participating in this clinical study, and that their dosage and administration adhere to the study protocol. Remaining drugs may be destroyed by the research institution with written authorization from the sponsor. Unused drugs must be returned to the sponsor and must not be transferred to any non-participants of the clinical study.

Monitors are responsible for overseeing the supply, use, storage, and disposal of remaining investigational drugs.

5.7.1 Transport / Receive / Store / Safeguard

Research drugs that have been appropriately packaged are sent by the sponsor to the clinical research center. During transport, the storage conditions and transport precautions for the drugs must be ensured, and there should be records of the transport conditions.

The handover of drugs between the sponsor and the research center should have a receipt record, with signatures and dates from both parties. The clinical research center should designate a dedicated drug administrator to be responsible for the management of drugs, such as receiving drugs and storing them in a dedicated refrigerator for trial drugs according to the storage conditions, ensuring unified safekeeping.

The refrigerator for storing drugs needs to maintain temperature records, covering the time range from the first receipt of the drugs to the last recovery, with a designated person responsible and signing off. The recording frequency should be reasonable.

5.7.2 Distribution / Use / Return / Destruction

All investigational drugs can only be distributed by the drug administrator, who must also record the quantity of drugs distributed. The person receiving the drugs must have a drug receipt

record, including the quantity of drugs received, the name of the recipient, and the date. The preparation of the drugs must have a record of the drugs prepared on that day.

Once the study is completed, the investigator must return all unused investigational drugs to the sponsor. The clinical monitor assigned by the sponsor will collect the drugs along with the corresponding usage records, record the quantity and usage of the returned drugs, sign, and date the records. The sponsor is responsible for the unified destruction of the drugs.

5.7.3 Records

During the entire study period, detailed records should be kept regarding the transportation, receipt, dispensing, collection, use, return, and destruction of the investigational drug. At the end of the study, it must be ensured that the transportation records of the investigational drug are consistent with the usage and destruction / return records; any discrepancies should be explained.

5.8 Other safety considerations and risk management

Investigators should administer the investigational drug to patients according to the study protocol; any medication errors should be immediately reported to the sponsor /CRO as protocol deviations. Medication errors can lead to AE/SAE occurrences or no clinical abnormal results.

For example:

- Drug overdose - If the investigator suspects that a patient has received an overdose of the investigational drug resulting in an AE/SAE, the investigator must take necessary clinical measures to ensure the patient's safety, while documenting the protocol violation, and should also record or report this AE/SAE. If the investigator suspects that a patient has received an overdose of the drug, and after close observation, the patient shows no clinical abnormal symptoms, only a protocol violation record is required.
- Drug diversion - Defined as the intentional or unintentional sale or giving of the investigational treatment to others. This may include the accidental misdelivery of investigational drugs to the hospital's routine main supply drug system. Any adverse events occurring in non-patient individuals after drug transfer must be handled according to the principles described in Chapter 7.

Any packaging or labeling found to pose potential risks (e.g., due to similarity with other products or unclear instructions) must be reported to the sponsor immediately.

6 Study procedures and evaluation criteria

6.1 Study procedures

Table1The following describes the study procedures that each patient recruited in the study will undergo, detailed in Table 1, with visits for these evaluations marked with an '×'. All visits will be scheduled starting from Day 1 of the investigational drug administration according to the appropriate calendar days. The allowable visit time window is ±3 days (except that no visit window is allowed on Day 1 of Cycle 1), and the time window for tumor assessment is ±7 days. Note: If the study treatment is suspended at any time during the study, safety and efficacy evaluations should continue according to the evaluation schedule based on the appropriate calendar days after Day 1 of the study.

All data obtained from these evaluations must be supported by the patients' original records. Any substantial deviation that may affect the safety of patients, the management of trial samples, or the assessment of safety, efficacy, and tolerance parameters should be promptly reported to the sponsor and the ethics committee of the research center (IRB).

6.1.1 Patient Screening

Informed consent must be obtained before any specific steps of the study are conducted. Research-specific evaluations and assessments can only be conducted after the patient indicates their voluntary participation in the clinical trial and signs a written informed consent form. The informed consent process, including the date of signing, must be documented in the patient's source documents. The source documents for the informed consent process must indicate whether informed consent was obtained prior to participation in this study.

After the patient signs the informed consent form, and before any study-related examinations are conducted, each patient will be assigned a unique screening number. The screening number consists of: center number (XX) + sequential number (XXX , for example, the third screened patient at center 02 will be 02003, which will be used for patient identification throughout the study. The patient screening number must not be reused. If a patient fails screening for special reasons and the investigator believes they can be re-screened for the study, a new screening number will be assigned, and a new informed consent form will be signed.

The principal investigator or designated assistant investigator will screen the eligibility of patients who meet the criteria for this trial according to the provisions of this study protocol to determine their suitability for participation. Re-testing of laboratory parameters that do not meet the inclusion criteria is allowed within the screening window (-28 days to -1 day) for patients.

After signing the informed consent form, completing all screening assessments, and confirming that the patients meet all inclusion criteria and do not meet any exclusion criteria, they will enter the treatment phase of the study.

6.1.1.1 Patient Screening Visit

Before entering the study, informed consent patients will be assessed to ensure they meet inclusion/exclusion criteria (see Chapter 4). These assessments must be completed within 28 days prior to the study medication. The investigator or designated assistant investigator must review and approve the results from the screening period prior to the administration of the investigational drug.

The following assessments must be completed within 28 days prior to the first administration of the investigational drug:

- Informed consent form;
- Demographics: including ethnicity, age (date of birth), gender, etc.;
- Past medical history, surgical history, treatment history: past medical history, treatment history, and surgical history within at least the last year;
- Tumor diagnosis: including diagnosis date, pathological diagnosis, staging (BCLC staging), and the site included in the study;
- Previous anti-tumor treatments: all previous anti-tumor treatments, including all surgeries, chemotherapy, biological therapy, immunotherapy, and radiotherapy;
- Height, weight;
- Vital signs;
- Physical examination;
- Assess and record ECOG performance status (refer to Appendix 14.1);
- 12-lead ECG examination;
- Cardiac echocardiogram: results obtained within 28 days prior to the first study medication are acceptable;
- Laboratory tests: complete blood count, urinalysis, blood biochemistry, coagulation function, thyroid function tests;

If urine protein ≥2+ in urinalysis, additional testing for 24-hour urine protein quantification is required.

- Collection of serum creatinine blood samples: Calculate the creatinine clearance rate (mL/min) using the serum creatinine measurement according to the following Cockcroft and Gault equation:

$$Ccr = \frac{(140 - \text{Age [years]}) \times \text{Actual Body Weight (kg)}}{72 \times \text{Serum Creatinine (mg/dL)}}$$

*For female patients, the result obtained from the above formula will be multiplied by 0.85.

- Pregnancy test: For women who are premenopausal or have not undergone surgical sterilization, the serum test must be negative within 7 days prior to the first administration of the study drug (if positive, pregnancy must be ruled out by ultrasound);
- HBV/HCV/HIV testing (results within 28 days prior to the first dose are acceptable): HBV antigen antibody tests (patients who are positive for HBsAg and/or HBcAb should undergo HBV DNA titer testing), anti-HCV antibody (patients who are positive for anti-HCV should undergo HCV RNA titer testing), and anti-HIV antibody tests;
- AFP: Applicable only to Phase III studies;
- Biological sample collection: Applicable only to Phase III studies, tumor specimens from patients will be collected during the screening period for PD-L1 testing (if available). Patients may choose to provide fresh biopsy tissue or archived tumor tissue from the most recent biopsy (within 2 years prior to signing the informed consent). If tumor tissue cannot be obtained for various reasons (e.g., exhausted due to previous diagnostic testing, high risk of puncture due to tumor location, etc.), it may not be provided;
- Gastrointestinal endoscopy: The investigator may assess the patients' portal hypertension and bleeding risk based on physical examination, imaging studies, and other comprehensive evaluations. For patients at high risk of bleeding, gastrointestinal endoscopy may be performed to understand the variceal condition and assist in evaluating the bleeding risk. Patients assessed to have a low bleeding risk may not undergo gastrointestinal endoscopy.
- Randomization: In the Phase III study, patients must receive the investigational drug treatment within 3 days after randomization.
- Record of combination therapy and treatment: All medications and clinically significant non-drug treatments (including physical therapy, oxygen therapy, and blood transfusions) administered after signing the informed consent must be recorded in the EDC under the

prior medication or non-drug treatment section. Any changes in medication should be continuously updated in the EDC.

- Measure and record measurable lesion parameters according to the following imaging examination process:
  - ✓ Enhanced CT of the chest, abdomen, and pelvis; if the patient is allergic to CT intravenous contrast agents, a CT chest scan combined with MRI of other areas (such as the abdomen) may be used;
  - ✓ Record other relevant imaging of disease sites outside the chest, abdomen, and pelvis (if applicable);
  - ✓ For patients with suspected brain metastases or bone metastases at baseline, consider performing enhanced CT and/or MRI of the brain and bone scans separately; for any positive areas identified on the bone scan (as determined by the investigator), evaluation must be conducted through enhanced CT or MRI.

All laboratory safety checks required by the protocol will be conducted in the laboratory of the research center. All laboratory results must be recorded in the EDC, and the normal value range for each laboratory, the model of the instruments used, and the laboratory qualification certificates must be provided and stored in the folders of the investigator and the sponsor.

The investigator should review the laboratory data, verify any significant deviations or data outside of clinically acceptable ranges, and promptly assess and note the clinical significance of any abnormal data.

6.1.1.2 Information to be collected for patients who fail screening

Patients who sign the informed consent form but fail to start the study treatment for any reason will be considered screening failure patients.

For screening failure patients, the following EDC must be completed:

- Screening phase allocation page (including reasons for not starting treatment);
- Informed consent;
- Demographics;
- Inclusion/exclusion criteria.

6.1.2 Study treatment period

Visits during the study treatment period include study drug visits, efficacy assessments every 6 weeks (every 9 weeks after the first study drug administration at 48 weeks), safety assessment visits every 2 cycles, and additional safety assessment visits for each treatment cycle.

6.1.2.1 Visit on Day 1 of Cycle 1

The visit on Day 1 of Cycle 1 does not allow for a time window; the following content must be completed during this visit:

- Weight: Weigh before medication to calculate the SCT510 dosage;
- Vital signs;
- Physical examination: Results from the screening period within 7 days prior to the first medication are acceptable;
- 12-lead ECG examination: Results from the screening period within 7 days prior to the first medication are acceptable;
- Assess and record ECOG performance status: Results from the screening period within 7 days prior to the first medication are acceptable;
- Laboratory tests: Complete blood count, urinalysis, blood biochemistry, coagulation function, thyroid function tests; results from the screening period within 3 days prior to the first medication are acceptable;
- Pregnancy test: For women who are not postmenopausal or have not undergone surgical sterilization, a serum β-human chorionic gonadotropin (β-hCG) pregnancy test is required, with acceptable screening results obtained within 7 days prior to the first dose;
- AFP: Applicable only to phase III studies, with results from examinations conducted within 7 days prior to randomization;
- Collection of serum creatinine blood samples: Creatinine clearance (mL/min) is calculated using the serum creatinine measurement based on the Cockcroft and Gault equation, with acceptable screening results obtained within 3 days prior to the first dose;
- Pharmacokinetics sample collection: Collect samples from the first 25 patients enrolled at designated research centers in the Phase II study, as well as from the first 150 patients in the Phase III trial group, with patients undergoing blood collection from the opposite side of the drug infusion; specific collection times are detailed in section 6.5;

- Immunogenicity testing sample collection: Samples to be collected within 1 hour prior to the first administration of the study drug (for the experimental group only);
- Collect information on adverse events;
- Collect information on combination therapy;
- Quality of life assessment: Applicable only to the Phase III study, with results to be completed by the patient within 3 days prior to the first administration of the study drug;
- Treatment with investigational drugs: The administration sequence for the combined treatment in the Phase II and III study groups is as follows: SCT-I10A, SCT510, with a flushing of the line after the infusion of SCT-I10A, followed by the infusion of SCT510. There should be an interval of more than 30 minutes between the end of the SCT-I10A infusion and the start of the SCT510 infusion. The Phase III control group will receive Sorafenib treatment.

Note: ECG and laboratory safety results must be obtained prior to drug treatment (laboratory blood samples should be collected according to local hospital requirements, either fasting or non-fasting).

6.1.2.2 Visit on Day 1 of Cycle 2 and subsequent cycles

The visit time window is ±3 days, and the following items need to be completed:

- Weight: Weigh before medication to calculate the SCT510 dosage; if weight change is <10%, no dosage adjustment is needed;
- Physical examination;
- Vital signs;
- 12-lead ECG examination;
- Assess and record the ECOG performance status;
- Laboratory tests: Complete blood count, blood biochemistry, urinalysis, thyroid function, coagulation function;

Among them, the complete blood count, blood biochemistry, and urinalysis must be obtained within 3 days before each cycle of medication; thyroid function and coagulation function are assessed before every 2 cycles of medication, such as on the 1st day of the 3rd, 5th, 7th, 9th cycles, with a time window of ±3 days;

- For patients who are HBsAg and/or HBcAb positive, HBV-DNA levels should be measured within 7 days before every 2 cycles of medication; for patients who are anti-

HCV positive, HCV-RNA levels should be measured within 7 days before every 2 cycles of medication;

- AFP: Only Phase III study, every 2 cycles, with a time window of ±7 days;
- Pregnancy test: For women who are not postmenopausal or have not undergone surgical sterilization, a urine/blood β-human chorionic gonadotropin (β-hCG) pregnancy test is required every 2 cycles, with a time window of ±3 days;
- Pharmacokinetics sample collection: Collect samples from the first 25 patients enrolled at designated research centers in the Phase II study, as well as from the first 150 patients in the Phase III trial group, with patients undergoing blood collection from the opposite side of the drug infusion; specific collection times are detailed in section 6.5;
- Immunogenicity sample collection: Blood samples will be collected at cycles 2, 4, 8, 12, 16, and subsequently every 8 cycles (e.g., at cycles 24, 32, etc.) and at the EOT visit, with collection occurring within 1 hour prior to the start of SCT-I10A infusion (only for the experimental group);
- Tumor assessment: See Section 6.2 for details, with a time window of ±7 days; tumor assessment results must be obtained prior to drug treatment.
- Collect information on adverse events;
- Collect information on combination therapy;
- Quality of life assessment: Applicable only to Phase III studies, conducted once every 2 cycles during the treatment period (window period ±7 days), to be completed by the participant.
- Treatment with investigational drugs: The administration sequence for the combined treatment in the Phase II and III study groups is as follows: SCT-I10A, SCT510, with a flushing of the line after the infusion of SCT-I10A, followed by the infusion of SCT510. There should be an interval of more than 30 minutes between the end of the SCT-I10A infusion and the start of the SCT510 infusion. The Phase III control group will receive Sorafenib treatment.

Note: Tumor imaging assessment results and pregnancy test results must be obtained prior to drug treatment (e.g., at every 6 weeks or 9 weeks for efficacy evaluation), along with ECG and laboratory safety results (laboratory blood samples should be collected according to local hospital requirements, either fasting or non-fasting).

6.1.3 End of Treatment Visit

When a patient continues treatment due to disease progression without clinical benefit, experiences intolerable toxicity, or needs to discontinue the investigational drug for any other reason, a treatment end visit must be completed within 7 days after the last dose of the investigational drug or within 7 days after being informed/confirming early withdrawal. This visit must complete the following process:

- If the following situations occur, refer to the provisions in Section 6.2 for efficacy evaluation:
  - ✓ Patients must have received at least one dose of the investigational drug (including both the experimental and control groups);
  - ✓ No new antitumor treatment targeting hepatocellular carcinoma has been initiated, and at least 4 weeks have passed since the last tumor assessment;
- Physical examination;
- Vital signs check;
- Weight;
- 12-lead ECG examination;
- Assess and record ECOG performance status (refer to Appendix 14.1);
- Laboratory tests: complete blood count, blood biochemistry, urinalysis, coagulation function, thyroid function;
- For patients who are HBsAg and/or HBcAb positive, measure HBV-DNA load; for patients who are anti-HCV positive, measure HCV-RNA load;
- AFP, only Phase III study;
- Quality of life assessment: only Phase III study, completed by the participant;
- Pregnancy test: for women who are not postmenopausal or have not undergone surgical sterilization, a serum β-human chorionic gonadotropin (β-hCG) pregnancy test is required;
- Pharmacokinetics sample collection: collect samples from the first 25 patients enrolled at the designated research centers in the Phase II study and from the first 150 patients who terminated treatment before 12 cycles in the Phase III study;
- Immunogenicity sample collection;

- Collect information on adverse events;
- Collect information on combination therapy.

6.1.4 Survival follow-up

Table1After the end-of-treatment visit, survival status will be followed up according to standard clinical treatment. After the end-of-treatment visit, patients who terminate study treatment for reasons other than disease progression, initiation of new anti-tumor therapy, death, or loss to follow-up should continue to undergo tumor assessments according to Table 1 until disease progression, initiation of new anti-tumor therapy, withdrawal of informed consent, death, or loss to follow-up (whichever occurs first). During this period, all adverse events for patients should be collected according to the time limits for adverse event collection outlined in section 7.1.1. All patients who have received at least one dose of the investigational drug (either the experimental drug or the control group Sorafenib) must undergo survival follow-up, with visits scheduled every 3 months (±14 days) after the end-of-treatment visit or the last administration date (only for patients who did not undergo an end-of-treatment visit), which can be conducted via telephone inquiries to collect information on new anti-tumor treatments and survival status starting from the end-of-treatment visit until death, loss to follow-up, or withdrawal of consent. The following processes / evaluations should be conducted at each survival follow-up visit:

- Collect information on severe adverse events related to the investigational drug;
- Record all subsequent treatments for hepatocellular carcinoma (if applicable);
- Record the date of death (if applicable).

6.1.4.1 Loss to follow-up

For patients whose status is unknown but who failed to appear for the study visit indicating an intention to withdraw consent, the investigator should attempt to contact the patient, family members, or family physician as agreed in the informed consent, and document the attempts to contact the patient in the source documents, such as the date of phone contact, WeChat, email, etc., demonstrating “due diligence”.

6.2 Efficacy assessment indicators

Will be based on FAS and PPS.

6.2.1 Tumor assessment time

According to the study flowchart, tumor efficacy assessments will be conducted at baseline before treatment initiation and every 6 weeks (±7 days) during the study treatment period (i.e., on Day 1 of Week 7, Week 13, Week 19, etc.), and after the first dose of the study treatment, tumor efficacy assessments will be conducted every 9 weeks (±7 days) on Day 1 until 48 weeks, continuing with radiologically based tumor assessments until disease progression, inability to benefit clinically, withdrawal of informed consent, death, or loss to follow-up. If disease progression is suspected, the frequency of assessments may be increased, and the additional assessment times and frequencies will be determined by the investigator based on clinical circumstances.

After baseline, all assessment windows for the evaluation plan are ±7 days. The same assessment methods and techniques should be used at baseline and during follow-up to understand the condition of each individual and report the lesions. If a patient experiences dose adjustments or delays, the frequency of imaging examinations or the scheduled dates for imaging examinations should not be changed.

If a patient interrupts treatment for reasons other than radiologically confirmed disease progression, an efficacy assessment should be conducted at the end-of-treatment visit, unless a tumor assessment was performed using CT/MRI within 28 days.

6.2.2 Tumor assessment requirements

At baseline, enhanced CT of the chest, abdomen, and pelvis will be performed (for patients allergic to contrast agents, MRI may be used); detailed technical requirements can be found in the BICR imaging manual provided by this study.

During the study period, enhanced CT of the chest, abdomen, and pelvis will be performed (for patients allergic to contrast agents, a CT chest scan combined with MRI of other areas may be used) to continuously monitor disease status. If there are clinical indications during the screening period, CT or MRI will be used to follow up on other metastatic disease sites (such as brain metastases). Bone scans will be conducted as clinically necessary.

For patients with possible bone metastases at baseline or suspected to have developed bone metastases during the trial, a bone scan should be considered. For any positive areas identified on the bone scan (as determined by the investigator), further evaluation must be conducted using enhanced CT or MRI.

Whenever possible, the same imaging method should be used to assess each lesion throughout the study (with or without intravenous (i.v.) and / oral contrast agents) and to evaluate tumor efficacy. If at baseline, the patient is known to have contraindications to CT i.v. contrast agents or if contraindications arise during the study, a non-contrast CT scan of the chest should be performed (due to respiratory artifacts, the use of MRI is not recommended), along with an enhanced abdominal and pelvic MRI examination using contrast agents (if possible).

Clinical assessment of any existing superficial lesions should be conducted during the screening period and at each subsequent tumor evaluation. Skin lesions should be measured using calipers and documented with clear-focus digital photography (color photos), displaying the size of the lesions using a scale or measuring device shown in the photographs along with the corresponding measurement methods. All skin lesions photographed at screening should continue to be photographed during subsequent tumor evaluations.

If imaging examinations listed above during screening and each subsequent tumor evaluation fail to capture images of other lesions (such as in the neck), separate CT or MRI examinations should be performed.

Ultrasound should not be used to measure tumor lesions.

If possible, all tumor efficacy assessments for individual patients should be conducted by the same radiologist.

Any lesions that have previously received radiotherapy will be considered non-target lesions unless there is clear disease progression after radiotherapy.

6.2.3 Blinded independent central imaging review

Only for the Phase III randomized controlled trial portion, all imaging images will be sent to a blinded independent central imaging laboratory (BICR) for blinded independent central image review as described in the BICR protocol. Specific details will be described in the BICR imaging manual and BICR protocol. BICR image review will assess the antitumor efficacy of the investigational drug based on RECISTv1.1 and mRECIST.

In addition, the investigators will also assess the antitumor efficacy of the investigational drug based on RECISTv1.1, independently of the BICR assessment.

6.2.4 Quality of life assessment

For the Phase III randomized controlled trial portion only, data on patient functioning, disease-related symptoms, health-related quality of life, and health status will be collected using

the EORTC QLQ-C30 (Appendix 14.6) and EORTC QLQ–HCC18 (Appendix 14.7) scales. Quality of life assessments at baseline and during treatment should be completed independently by the patient as much as possible, either before any trial-specific evaluations are conducted or before discussions between the patient and trial personnel regarding how the patient feels, using the EORTC QLQ-C30 and EORTC QLQ–HCC18 scales.

6.3 Safety assessment

Will be based on SS.

6.3.1 Weight and height

Weight will be measured during the screening period, at study treatment visits, and at the end of treatment visit, while height will only be recorded during the screening visit.

6.3.2 Vital signs

The patient's temperature, blood pressure, pulse, and respiratory rate will be recorded. Among them:

- The pulse detection time is 1 minute.
- All blood pressure measurements should be taken from the same arm, preferably by the same examiner, to minimize errors.

6.3.3 Physical examination

The physical examination record includes: general condition, skin and mucous membranes, lymph nodes, head and neck (including thyroid), chest (heart, lungs), abdomen (liver, gallbladder, spleen, kidneys), musculoskeletal system, and nervous system.

According to TableTable1

During the physical examination, attention should be paid to any discomfort of the patients' skin; if any abnormalities occur, it is recommended to promptly conduct relevant specialist examinations.

The study treatment period did not design physical examination visits to be conducted only when clinical symptoms arise, as deemed necessary by the investigator.

6.3.4 ECOG Score

Patients are assessed according to the Appendix 14.1 ECOG Performance Status Scoring Criteria.

6.3.5 12-ECG

Table1A 12 -lead resting electrocardiogram must be taken after resting for 5 minutes before each examination. Detailed examination times are shown in Table 1, and the following ECG parameters need to be recorded: heart rate, PR interval, QRS interval, QT interval, corrected QT interval, and the principal investigator's assessment of the ECG tracing. If there are abnormalities in the electrocardiogram during the visit examination, it is recommended to recheck within 24 hours.

6.3.6 Cardiac echocardiography

Assess left ventricular ejection fraction (LVEF).

6.3.7 Laboratory tests

6.3.7.1 Complete blood count

Includes: red blood cell count (RBC), hemoglobin (HGB), white blood cell count (WBC), platelet count (PLT), absolute neutrophil count (NEUT#), absolute lymphocyte count (LYMPH#), absolute monocyte count (MONO#), and percentage of neutrophils (NEUT%).

6.3.7.2 Urinalysis

Includes: urine protein (U-PRO), urine glucose (U-GLU), urine white blood cells (U-WBC), urine red blood cells (U-RBC). If urine protein ≥2+, a 24-hour urine protein quantification test is required.

6.3.7.3 Blood biochemistry

Includes: alanine aminotransferase (ALT), aspartate aminotransferase (AST), alkaline phosphatase (ALP), gamma-glutamyl transferase (GGT), lipase (LPS), amylase (AMY or AMS), urea (UREA), serum creatinine (CREA) and calculation of creatinine clearance (using the Cockcroft-Gault formula, see Appendix 14.4), total protein (TP), serum albumin (ALB), total bilirubin (TBIL), direct bilirubin (DBIL), creatine kinase (CK), fasting blood glucose (Glu), potassium (K), sodium (Na), chloride (Cl), magnesium (Mg), calcium (Ca), phosphorus (P).

6.3.7.4 Coagulation function

Includes: Prothrombin time (PT), Activated partial thromboplastin time (APTT), D- -dimer, FIB, International normalized ratio (INR).

6.3.7.5 Thyroid function

Includes: Serum Thyroid Stimulating Hormone (TSH), Serum Free Triiodothyronine (FT3), Serum Free Thyroxine (FT4).

6.3.7.6 Immunological tests

Immunological tests: Hepatitis B five items (HBsAg, HBsAb, HBeAg, HBeAb, HBcAb) and / or HBV DNA, Anti HCV antibody and / or HCV RNA titer tests, Anti HIV antibody tests; Immunological tests are conducted during the screening period. During the screening period, patients who are HBsAg positive and/or HBcAb positive should have their HBV-DNA levels measured within 7 days prior to each treatment cycle and at the end of treatment visit. Patients who are anti-HCV positive should also have their HCV-RNA levels measured during the screening period and within 7 days prior to each treatment cycle and at the end of treatment visit.

6.3.7.7 AFP

AFP testing: In the Phase III study, AFP levels should be measured during the screening period (obtained within 7 days prior to randomization) and every 2 cycles (±7 days) after treatment until disease progression, no longer clinically benefiting, initiation of new anti-tumor treatment, withdrawal of informed consent, death, or loss to follow-up, whichever occurs first.

6.3.7.8 Pregnancy test

Female patients of childbearing potential must undergo a pregnancy test (serum) during the screening period, and the pregnancy test result must be negative to be eligible for this study. During the treatment period visits, pregnancy tests may be conducted using either serum or urine analysis. A pregnancy test (serum) must be conducted again at the end of the investigational drug treatment visit. All females of childbearing potential must undergo pregnancy testing. Those without childbearing potential must be noted in the original documents.

The definition of menopause must meet at least one of the following criteria:

- Post bilateral oophorectomy;
- Not undergone bilateral oophorectomy, age ≥ 60 years;
- Age < 60 years, without chemotherapy and the use of tamoxifen, toremifene, and ovarian function suppression treatment, having been postmenopausal for more than one year, while follicle-stimulating hormone (FSH) and estradiol levels are within the postmenopausal range;

- Age < 60 years, if using tamoxifen or toremifene, but follicle-stimulating hormone (FSH) and estradiol levels are within the postmenopausal range;

The timing of the above laboratory tests can be found in the study flowchart.

6.3.8 Adverse events

All adverse medical events will be collected from the time the patients receive the investigational drug until 90 days after the last dose or before the start of any new anti-tumor treatment, whichever occurs first. After this period, only severe adverse events related to the investigational drug or study procedures need to be collected.

During the trial, the symptoms, signs, and relevant laboratory and auxiliary examinations of the patients will be closely monitored. The occurrence time, severity, relationship with the investigational drug, measures taken, outcomes, etc., of adverse events will be recorded in detail.

6.3.9 Concomitant medication and treatment

Record any concomitant treatment received by the patients from the date of informed consent until 90 days after the last dose or before starting a new anti-tumor therapy (whichever occurs first). Patients are required to record the status of combination therapy / treatment at each visit.

6.4 Biomarkers

During the screening period, tumor tissue from patients (if available) will be collected for PD-L1 immunohistochemical testing to evaluate the correlation between PD-L1 and efficacy, prognosis.

6.5 Pharmacokinetic sample collection and analysis

The pharmacokinetic characteristics of the two drugs in patients will be evaluated using a fully validated ELISA method to detect SCT-I10A and SCT510 in human serum.

Phase II designated research centers enrollment prior to 25 patients PK blood sample collection:

Intensive sampling during 1 and 4 cycles: Patients should have samples collected at 1 and 4 hours before the administration of SCT-I10A in each dosing cycle, immediately after the completion of each drug infusion of SCT-I10A and SCT510 (within +5min), as well as at 6 hours (±30min), 24 hours (±1h), 96 hours (±2h), 168 hours (±24 hours), 336 hours (±24 hours), and 504 hours (±24 hours) after the administration of SCT-I10A.

Sampling in the 2, 5, 6, 8, and 12 cycles: SCT-I10A within 1 hour before administration.

The sampling time points for the 2 and 5 cycles overlap with the 22 day of the intensive sampling in the 1 and 4 cycles, so no additional sampling is required; However, if the administration in the 2 and 5 cycles is delayed due to AE or other reasons, a PK blood sample must be collected within 1h before the start of SCT-I10A infusion in the 2 and 5 cycles.

If a patient terminates treatment early between the 5-11 cycles, a PK blood sample will be collected during the end-of-treatment visit.

Phase III Patient PK Blood Sample Collection:

Only the first 150 patients in the experimental group will have PK blood samples collected. The collection time for Phase III patient PK blood samples is:

Before the start of the first cycle SCT-I10A infusion, within 1 hour, and immediately after the end of SCT510 infusion (+5 min). Before the start of the second, fourth, eighth, and twelfth cycles, within 1 hour of SCT-I10A infusion.

If a patient terminates treatment early between the 5-11 cycles, a PK blood sample will be collected during the end-of-treatment visit.

For the collection, processing, preservation, and transportation of II and III phase PK samples, please refer to the 'Laboratory Manual' provided by the central laboratory.

6.6 Immunogenicity

Using immunogenicity detection methods validated in previous clinical studies, we will assess the immunogenicity by detecting anti-SCT-I10A antibodies and anti-SCT510 antibodies in the serum of patients before and after treatment.

Blood sample collection time: Patients should collect blood samples at the 1st, 2nd, 4th, 8th, 12th, 16th cycles, and subsequently every 8 cycles (such as the 24th cycle, 32nd cycle, etc.) and at the end of treatment visit. The collection time is within 1 hour before SCT-I10A infusion begins.

For the collection, processing, preservation, and transportation of immunogenicity samples in Phase II and III, please refer to the 'Laboratory Manual' provided by the central laboratory.

7 Adverse events and severe adverse events

During the clinical trial, investigators need to closely observe, analyze, and record clinical adverse events/severe adverse events and abnormal laboratory test/other special examination values that occur during the trial. Clinical adverse events are assessed for their association with the investigational drug according to the standards for adverse reaction/event analysis. Specific definitions, procedures, and time requirements are detailed in the following content.

7.1 Adverse events

Adverse events refer to all unfavorable medical occurrences that arise after patients receive the investigational drug, which may manifest as symptoms, signs, diseases, or laboratory test abnormalities, but are not necessarily causally related to the investigational drug.

An AE can be an unfavorable sign (including abnormal laboratory tests, etc.) or symptom or disease that is unrelated to the intended use of the drug, with a temporal relationship to drug administration, regardless of whether there is a causal relationship with the drug. All observed adverse events or those actively reported by the patients should be recorded in the original medical records and entered into the adverse event section of the case report form.

7.1.1 Timeframe for adverse event collection

Collect all adverse medical events, including severe adverse events, from the time the patients receive the investigational drug until 90 days after the last dose or before the start of any new anti-tumor treatment, whichever occurs first, regardless of whether they are observed by the investigator or spontaneously reported by the patients. After this period, only severe adverse events related to the investigational drug or study procedures need to be collected.

For each adverse event, the information that needs to be collected and recorded includes but is not limited to: AE name, occurrence time, severity, end time, management measures, whether it is a severe adverse event, and event outcome. The investigator needs to analyze and determine the causal relationship with the investigational drug and whether the criteria for severe adverse events have been met. If the criteria for severe adverse events are met, they must be recorded and reported according to the severe adverse event reporting process.

7.1.2 Follow-up of adverse events

All adverse events (including laboratory test abnormalities) must be followed up until any of the following conditions are met:

- Event resolved;

- Event stabilized;
- Event returned to baseline level;
- The investigator reasonably believes (for example, that it cannot be restored or has improved) that further follow-up is unnecessary;
- When no further information can be obtained (for example, if the patient refuses to provide more information, or there is evidence that after making every effort the patient is still lost to follow-up).

7.1.3 Description of adverse events

The description of matters regarding AE records is as follows:

Diagnosis, symptoms, and signs

If a diagnosis already exists, the AE name should record the diagnosis name rather than individual symptoms and signs (for example, record liver failure instead of jaundice, elevated transaminases, and asterixis). If symptoms and signs cannot be determined to be caused by the diagnosis at the time of reporting, they should be recorded as separate AE/SAE. If during the AE/SAE follow-up process it is determined that the symptoms and signs are caused by the diagnosis, only the diagnosis should be reported separately, and the symptoms and signs will be included in the diagnosis, no longer recorded as AE; for SAE, a follow-up update report is required.

Persistent or recurrent adverse events

Persistent adverse events refer to adverse events that have not resolved and continue to exist between two evaluation time points for the patient.

Recurrent adverse events refer to adverse events that, according to the investigator's judgment, have resolved between two evaluation time points but then reoccurred. Events should be recorded separately.

Disease progression

The diseases studied in this trial, expected disease progression, or symptoms and signs caused by disease progression (excluding death caused by disease progression) are not to be reported as adverse events. For medical events that meet the SAE reporting criteria, if the investigator determines that the medical event is caused by the progression of the primary tumor disease, it may not be reported as SAE; If the investigator cannot clearly determine whether the medical event is caused by the progression of the primary tumor disease, it should be reported as SAE to the sponsor and recorded on the adverse event page of the eCRF.

Death

In this study protocol, death is considered a primary efficacy endpoint. Deaths occurring during the adverse event collection period, confirmed by the investigator to be caused by the progression of the primary disease, will only be recorded on the death record page of the eCRF and will not be reported as SAE to the sponsor; All other deaths occurring during the adverse event collection period, regardless of whether they are related to the investigational drug, should be recorded on the adverse event page of the eCRF and promptly reported to the sponsor; All deaths occurring outside the adverse event collection period are recorded on the eCRF death record page.

When recording death events, if the cause of death is clear, the cause of death should be recorded as an adverse event, with the outcome of that adverse event being death; If the cause of death is unknown at the time of reporting, it should be recorded on the eCRF adverse event form as 'Death of unknown cause', and further investigation should be conducted to determine the exact cause of death. If the cause of death becomes clear later (e.g., after autopsy), the previously recorded 'Death of unknown cause' should be replaced with the determined cause of death.

Pre-existing medical conditions

Symptoms / signs that patients had during the trial screening period should only be recorded and reported as adverse events if there is an exacerbation in severity, frequency, and nature (excluding the worsening of the studied disease condition) after the use of the investigational drug. The records should reflect changes relative to the previous state, for example " increased frequency of headaches ".

7.1.4 Analysis of the association between adverse events and the investigational drug

The analysis of the association between adverse events and the investigational drug must consider the following factors:

- Whether there is a reasonable temporal sequence between the occurrence of adverse events and the use of the investigational drug;
- After the discontinuation or dose reduction of the investigational drug, did the adverse event lessen or disappear;
- After re-administration of the drug, did the adverse event reoccur;

- Do the clinical or pathological manifestations of the adverse event correspond with the known pharmacological and toxicological knowledge of the investigational drug or its class;
- Can the adverse event be explained by the original disease, the patient themselves, or environmental factors;

There are the following situations regarding the association of adverse events with the investigational drug:

- Definitely related: there is evidence of the use of the investigational drug; The occurrence of the adverse event has a reasonable temporal sequence with the use of the investigational drug, and the explanation of the adverse event by the use of the investigational drug is more reasonable than explanations based on other causes. The withdrawal reaction may lessen or disappear, and re-administration (if feasible) may lead to a recurrence of the reaction. This is consistent with known types of drug adverse reactions.
- Possibly related: Evidence of the use of the investigational drug; The occurrence of adverse events is reasonably correlated with the timing of the use of the investigational drug. Adverse events may be explained by other causes. Withdrawal reactions may potentially lessen or disappear.
- Possibly unrelated: Evidence of the use of the investigational drug; the adverse events that occurred may be better explained by other causes. Withdrawal reactions may potentially lessen or disappear or remain unclear.
- Definitely unrelated: The investigational drug was not used, or there is no correlation between the timing of the use of the investigational drug and the occurrence of adverse events, or there is another clear cause of the adverse events.
- Unable to determine: too many missing items in the report, causality difficult to conclude, and the data cannot be supplemented.

7.1.5 Handling of abnormal laboratory test results

During the clinical trial, if patients experience abnormal laboratory test results after receiving the investigational drug treatment, and these results are deemed clinically significant, they should be reported as adverse events (AE). The investigator is responsible for reviewing all laboratory results and determining whether the corresponding laboratory test abnormalities need to be

reported as AE. When the definition of AE is met, it must be recorded on the eCRF 'Adverse Events' page.

Laboratory test abnormalities that meet any of the following conditions are generally considered clinically significant:

- These abnormalities led to changes in the administration schedule of the investigational drug (dosage changes, delayed administration, suspension, or permanent discontinuation of the investigational drug);
- These abnormalities require other medical or therapeutic interventions (for example, anemia requiring blood transfusion or hyperglycemia requiring potassium supplementation);
- These abnormalities suggest new diseases or exacerbation of pre-existing comorbidities.

The principles for managing the aforementioned laboratory test abnormal values also apply to the management of abnormal vital signs and physical examination findings.

7.1.6 Severity of adverse events

The severity of all adverse events must be graded according to the Common Terminology Criteria for Adverse Events (CTCAE, version 5.0, 2017/11/27, refer to Appendix 14.3) published by the National Cancer Institute (NCI). For adverse events not listed in the NCI-CTCAE, please refer to Table Table 9 for grading:

Table 9 Adverse Event Evaluation Grading Criteria

Grade 1	Mild; asymptomatic or mild symptoms; only clinical or diagnostic findings; no treatment required;
Grade 2	Moderate; requires minor, local, or non-invasive treatment; limitations in instrumental activities of daily living comparable to age*;
Grade 3	Severe or of significant medical importance, but not immediately life-threatening; leads to hospitalization or prolongs hospitalization; causes disability; limitations in activities of daily living requiring self-care**;
Grade 4	Life-threatening, requiring urgent treatment;
Grade 5	Death.

*Instrumental activities of daily living refer to tasks such as cooking, shopping for groceries or clothes, using the phone, managing finances, etc.

**Activities of daily living refer to tasks such as bathing, dressing and undressing, eating, grooming, taking medication, and not being bedridden.

7.1.7 Outcome of adverse events

The outcomes of adverse events are as follows: recovery, recovery with sequelae, improvement, no improvement, death, unknown.

7.2 Severe adverse events

7.2.1 Definition

Severe adverse events (SAE) are defined as adverse events that result in the following outcomes after the patient receives the investigational drug:

- Resulting in death;
- Life-threatening (defined as 'life-threatening' when it poses an immediate risk of death to the patient, not referring to a hypothetical severe progression that may lead to death in the future);
- Requires hospitalization or prolongation of existing hospitalization;
- Leads to permanent or significant disability or loss of ability;
- Causes congenital anomalies or birth defects;
- Other important medical events (some important medical events may not necessarily lead to death, be life-threatening, or require hospitalization, but may endanger the patient or require medical intervention to prevent any of the aforementioned situations from occurring, and are typically considered SAEs).

Some events that require hospitalization or an extended hospital stay may not be classified as severe adverse events, including: hospitalization or extended stay for reasons other than adverse events, pre-scheduled surgeries before the trial, or hospitalizations not required by the condition but due to insurance reimbursement reasons; however, all of the above situations must be clearly documented in the medical records.

7.2.2 Reporting of severe adverse events

When a severe adverse event occurs, the investigator should take the following measures:

- Immediately take appropriate medical measures;
- Immediately (within 24 hours of the research center becoming aware of the event or new information regarding the event) complete and submit the 'Severe Adverse Event Report Form' to the sponsor. The sponsor's SAE reporting email is: SCT-

safety@sinocelltech.com (it is recommended to encrypt the SAE report when sending the email, and to send the password in the second email after reporting the SAE to the sponsor);

- Record severe adverse events in the original medical records and enter them in the adverse events section of the case report form.
- Follow up and document the process of the event until it resolves, is lost to follow-up, or returns to a state that the investigator deems no longer requires follow-up.

7.2.3 Follow-up of severe adverse events

All severe adverse events must be followed up until any one of the following conditions is met:

- Event resolved;
- Event stabilized;
- Event returned to baseline level;
- The investigator reasonably believes (for example, that it cannot be restored or has improved) that further follow-up is unnecessary;
- When no further information can be obtained (for example, if the patient refuses to provide more information, or there is evidence that after making every effort the patient is still lost to follow-up).

7.3 Suspected unexpected serious adverse reactions

7.3.1 Definition

Suspected unexpected adverse reactions refer to serious adverse reactions whose nature and severity exceed the information available in the investigator's brochure for the investigational drug, the product insert for marketed drugs, or the summary of product characteristics, and are considered suspicious and unexpected.

7.3.2 Report of suspected unexpected serious adverse reactions

After the sponsor becomes aware of a serious adverse event, a comprehensive analysis, assessment, and medical judgment of the serious adverse event should be conducted immediately. During the clinical trial of the drug, any unexpected and serious adverse reactions that are determined to be definitely related or possibly related to the investigational drug must be reported quickly in the form of individual safety reports.

The start time for the rapid reporting of suspected unexpected serious adverse reactions is the date of clinical trial approval / the date of implied permission from the national drug review agency, and the end time is the date of the last patient's follow-up. Serious adverse events related to the investigational drug or study procedures that occur after the end of the clinical trial or follow-up should be reported by the investigator to the sponsor, who will enter the collected SAE into the sponsor's safety database. If they are classified as suspected unexpected serious adverse reactions, they should also be rapidly reported to the regulatory agency.

The sponsor should rapidly report suspected and unexpected serious adverse reactions to the drug supervision and management department and the health administration department according to the nature (category) of the serious adverse events within the following time limits:

- For unexpected serious adverse reactions that are fatal or life-threatening, the sponsor should report as soon as possible after first knowledge, but no later than 7 days, and report and complete follow-up information within the subsequent 8 days.

Note: The day the applicant first becomes aware is Day 0.

- For unexpected serious adverse reactions that are not fatal or life-threatening, the applicant should report as soon as possible after first knowledge, but no later than 15 days.

At the same time, the sponsor should promptly report any suspected and unexpected serious adverse reactions to all researchers and clinical trial institutions participating in the clinical trial, as well as to the ethics committee, in accordance with the requirements and timelines of each center.

7.3.3 Follow-up of unexpected serious adverse reactions

The sponsor should continue to track unexpected serious adverse reactions after the first report and submit timely reports of new information or changes to previous reports in the form of follow-up reports, with a reporting deadline of within 15 days of obtaining new information.

All unexpected serious adverse reactions must be followed up until any of the following conditions are met:

- Event resolved;
- Event stabilized;
- Event returned to baseline level;

- The investigator reasonably believes (for example, that it cannot be restored or has improved) that further follow-up is unnecessary;
- When no further information can be obtained (for example, if the patient refuses to provide more information, or there is evidence that after making every effort the patient is still lost to follow-up).

7.4 Management of pregnancy events

If a female patient or the spouse of a male patient becomes pregnant within 120 days from the start of the first use of the investigational drug to the last use of the investigational drug, the investigator must complete the 'Clinical Trial Pregnancy Report / Follow-up Form' and report it to the sponsor within 24 hours of becoming aware. Researchers should continuously monitor female patients who become pregnant / the spouses of male patients and follow up on pregnancy outcomes; all pregnancy events must be followed up until the pregnancy concludes / terminates, and the results reported to the sponsor.

All patients participating in this clinical study who are of childbearing potential must take effective contraceptive measures. During the clinical trial, if a female patient exposed to the investigational drug becomes pregnant, the patient should immediately discontinue the study treatment. During the clinical trial, male patients exposed to the investigational drug may continue in the trial if their partners become pregnant.

Spontaneous abortion, selective abortion, induction of labor, ectopic pregnancy, and any fetal abnormalities (e.g., fetal demise, molar pregnancy, stillbirth, stillbirth, fetal malformations) are all considered SAE and must be reported according to the SAE reporting procedures. These events must be followed up until they resolve or stabilize.

7.5 Special attention to adverse events

The special attention adverse events for the investigational drug SCT-I10A include immune-related adverse events (irAE) of severity ≥ grade 3 and infusion reactions of ≥ grade 3, which should be closely monitored during the study. For immune-related adverse events, please refer to the 2019 CSCO guidelines for the management of toxicities related to immune checkpoint inhibitors.

The investigational drug SCT510 has special attention to adverse events including but not limited to the following events: gastrointestinal perforation and fistula, non-gastrointestinal fistula, reversible posterior leukoencephalopathy syndrome (PRES), bleeding (including any grade of

central nervous system bleeding, ≥ grade 2 hemoptysis, other ≥ grade 3 bleeding events), any grade of arterial thromboembolism, ≥ grade 3 venous thromboembolism, ≥ grade 3 hypertension, ≥ grade 3 congestive heart failure (CHF), ≥ grade 3 surgical and / or wound healing complications, ≥ grade 3 proteinuria, ≥ grade 3 infusion reactions, ≥ grade 3 infections, and ovarian failure.

The severity grading of adverse events of special concern is implemented according to CTCAE5.0 version.

Investigators must report adverse events of special concern to the sponsor within 3 days of becoming aware, so that the sponsor can timely assess and take appropriate actions regarding potential or new risks in the clinical trial to protect the interests of the patients. If an adverse event of special concern meets the SAE criteria, it must be reported according to the SAE reporting timelines and procedures.

8 Data management

The primary responsibility of the investigator is to ensure that the data reported in EDC or other forms is accurate, complete, and timely, and that the data on the EDC originates from the patients' source data.

This study will utilize an Electronic Data Collection (EDC) system for data collection and management. The data management process should comply with the Good Clinical Practice (GCP) guidelines and relevant regulatory requirements, following the standard operating procedures (SOP) of the data management department to ensure the authenticity, accuracy, completeness, reliability, and traceability of clinical trial data (the EDC system will record all audit trails). Detailed information on data management will be provided in the data management plan.

8.1 Data Management Plan

This study's data management plan was written by the Data Manager (DM) as a guiding document for the entire data management process. All data management processes should be conducted according to the data management plan, which should be updated based on the specific circumstances of the project. According to the protocol requirements, design the data collection case report form (Case Report Form CRF) and simultaneously create the corresponding data collection filling guidelines, which will be implemented after review and approval by the sponsor.

8.2 CRF and database

Data management personnel establish the database (eCRF) according to the study protocol, and the database administrator conducts database testing. EDC System users are controlled by role permissions and passwords. All accessing EDC users must fill out a user account application form, and the system administrator (Admin) creates accounts for project management personnel and grants them the corresponding account permissions and passwords for project management. Researchers have data approval and electronic signature permissions.

8.3 Data entry and verification

In this study, data entry is performed directly into the electronic data collection system (Electronic Data Collection, EDC). Researchers or CRC accurately, timely, completely, and normatively fill in the patient information through the EDC system based on the original data of the patients.

Clinical monitors should oversee whether clinical trials adhere to the study protocol, conduct Source Document Verification (SDV), and confirm that all EDC entries are consistent with the original documents. If there are errors or discrepancies, the investigator should be notified, and corresponding queries should be recorded based on the identified errors or discrepancies to ensure that all data is recorded and reported accurately and completely.

8.4 Data Verification and Query Management

Verify the reliability, completeness, and accuracy of the data in the EDC according to the requirements of the data verification plan, which mainly includes manual verification and logical verification by computer systems. Queries generated from the verification will be communicated to the research center in the form of data queries. They need to be answered promptly by the investigator or an authorized CRC. If the investigator's response resolves the query, the data manager will close the query; if the query remains unresolved, it will be issued again. This process continues until data cleaning is complete. All queries must be closed before the database is locked. After all data collection is completed, the data quality control personnel will conduct a comprehensive quality check on the data according to the QC plan, and the database QC must be completed before the database is locked.

8.5 External data and medical coding

The external data in this study is managed according to the external data transfer protocol. Adverse events requiring medical coding, past diseases, and surgical history will be coded according to the latest version of MedDRA, and combination therapy will be coded according to WHO Drug.

8.6 Database lock

According to the database locking process, once all pre-lock steps are completed, the database lock must be approved in writing, and the data editing permissions for the database must be revoked before the database is locked. If issues discovered after the database is locked need to be modified, the modifications should strictly follow the process of unlocking and relocking the database.

9 Statistical analysis

The detailed statistical analysis methods and data summary for this study will be included in the Statistical Analysis Plan (SAP). Any changes to this study protocol that are deemed by the sponsor or principal investigator to have a significant impact on the statistical analysis plan must be revised to maintain consistency with the SAP. The statistical analysis plan may revise relevant content in this protocol; however, if the revisions involve major and / or key factors such as the definition of endpoints or their analysis, such revisions may also be reflected in the protocol amendment.

9.1 Sample size

The Phase II study serves as a safety introduction phase, primarily assessing the safety of SCT-I10A combined with SCT510 treatment, with a planned enrollment of 50-60 patients.

The Phase III study references data from the IMbrave150 study in the Chinese population [1], where the Atezolizumab combined with Bevacizumab group compared to the Sorafenib group had an mPFS of 5.7 vs 3.2 months (HR=0.6), and an mOS of 24 vs 11.4 months, HR=0.53. The ORIENT-32 study data showed that the combination of Sintilimab and Bevacizumab biosimilar compared to Sorafenib had an mPFS of 4.6 vs 2.8 months (HR=0.565), with the experimental group not reaching mOS, while the control group was 10.4 months, HR=0.569. Additionally, referencing the REFLECT study data in the Chinese population, the Sorafenib group had a PFS of 3.6 months and an OS of 10.2 months, along with previous related studies indicating that Sorafenib had a PFS of approximately 3.5 months and an OS of 10-11.5 months in the Chinese population.

This study is designed as a superiority parallel trial, with the primary objective of evaluating the efficacy of the experimental group (SCT-I10A combined with SCT510) compared to the control group (Sorafenib) in the target patients. The primary endpoints are set as a dual endpoint consisting of progression-free survival (PFS) and overall survival (OS) assessed by BICR. According to previous literature, the median PFS (mPFS) of Sorafenib in the Chinese population is approximately 3.5 months, and the median OS (mOS) is 10-11.5 months. Assuming that the mPFS of the control group in this study is 3.6 months and the mPFS of the experimental group is 5.4 months, with a hazard ratio (HR) of 0.67, the mOS of the control group is 12 months and the mOS of the experimental group is 17.9 months, also with an HR of 0.67. The two-sided significance level is set at 0.05, and the analysis of PFS and OS will be conducted using a fixed-sequential procedure. Assuming an enrollment period of 12 months, after the random enrollment

of the first patient, approximately 16 months will be used for the two-sided significance level of 0.05 for the hypothesis testing of PFS. Therefore, when 342 patients (with a 2:1 enrollment ratio for the experimental and control groups) are enrolled and 252 PFS events are reached, there will be at least an 85% confidence level to detect the superiority hypothesis of the experimental group relative to the control group regarding PFS. When the null hypothesis of PFS is rejected, a hypothesis test for OS will be conducted approximately 36 months after randomization using a two-sided significance level of 0.05, thereby providing an 85% confidence level to detect the superiority hypothesis of OS in the experimental group compared to the control group. It is planned that during the final analysis of PFS, it is expected that 50% of the required OS events will be observed for an interim analysis of the OS endpoint, and the approximate Lan-DeMets α spending function will be used for the interim analysis effectiveness judgment , specifically using a two-sided significance level of 0.0028 for the interim analysis effectiveness judgment, with the final analysis of OS having a two-sided significance level of 0.049.

Adjustments to the expected number of enrolled patients and events may be further detailed in the statistical analysis plan, considering the delayed effects of subsequent crossover treatments and immunotherapy.

9.2 Analysis Population

Full analysis set (Full analysis set, FAS): Includes all patients assigned to the study according to the intention-to-treat (ITT) principle, who were randomized and signed the informed consent form and successfully screened, and who received at least one dose of the investigational drug. The full analysis set is the primary analysis population for this study. In analyses based on the full analysis set, patients will be assigned to the groups according to their randomization allocation (regardless of the actual treatment they received) for participation in the analysis.

Per-protocol set (PPS): This is a subset of the full analysis set consisting of patients who are more compliant with the protocol. The patients included generally require the following characteristics: completion of the minimum exposure to the specified investigational drug; availability of data for the primary endpoint indicators in the trial; no significant violations of the trial protocol. The analysis based on the per-protocol set will serve as a supportive analysis for this study.

Safety set (SS): This includes all patients who received at least one dose of the investigational drug and had at least one safety assessment after dosing, grouped according to the actual investigational drug taken. The safety set is used for the safety data analysis in this study.

Pharmacokinetic analysis set (PK analysis set, PKS): includes all patients who received at least one dose of the study drug and have at least one post-treatment pharmacokinetic evaluation data.

9.3 Statistical analysis methods

9.3.1 General principles

This study employs a seamless design for Phase II / III, with Phase II and Phase III data analyzed separately, using SAS 9.4 or higher for programming calculations.

Statistical description: For quantitative variables, list the number of cases, mean, standard deviation, median, upper quartile (Q1), lower quartile (Q3), minimum, and maximum. For categorical variables, list their frequency and percentage.

Statistical inference: Appropriate statistical analysis methods should be employed for intra-group or inter-group comparisons based on different applicable conditions, providing descriptive statistics, confidence intervals, and test statistics for inter-group comparisons, as well as P values, etc.

9.3.2 Case distribution

Summarize the screening and randomization status of patients; Reasons for screening failure, termination of treatment / termination of the study, and a summary of the reasons for termination.

Summarize the case distribution of the statistical analysis dataset and the reasons for exclusion, listing the situation of excluded patients (e.g., group, randomization date, drug exposure amount, reasons for exclusion, etc.)

Summarize the serious protocol violations during the study period.

9.3.3 Baseline and demographic characteristics

The Phase II study is based on SS, and the Phase III study is based on FAS, summarizing and analyzing demographic data (gender, age, height, weight, etc.) and other baseline characteristic data (including disease characteristics).

Summarize relevant medical history and current medical conditions classified by preferred terms (latest Chinese version of MedDRA).

9.3.4 Compliance and drug exposure analysis

Evaluate compliance with the protocol through the number and proportion of patients with protocol deviations; The compliance of the investigational drug will be assessed based on the

number of dose reductions and treatment pauses. The analysis will be presented in both list and summary formats.

A summary of the actual dosages, treatment days, and compliance of various investigational drugs will be compiled (compliance will be categorized as < 80%, 80%-100%, 100%-120%, and > 120%, with the number and proportion of patients in each category provided).

A summary of the reasons for treatment discontinuation will be compiled, listing the dates of the first and last administration, duration of exposure, and the discontinuation dates for each patient.

9.3.5 Efficacy analysis

Phase II is a single-arm study, with efficacy indicators serving as secondary endpoints, primarily evaluated using descriptive statistics; the following section mainly pertains to the III phase study. Efficacy analysis will be based on FAS, with PPS as a supportive analysis.

9.3.5.1 Definition of efficacy assessment

Overall survival (OS)

Overall survival (OS) is defined as the time from the first administration of the investigational drug to the date of death from any cause. For patients alive at the end of the study or those followed up, the OS time will be censored at the last contact date.

Progression-free survival (PFS)

Progression-free survival (PFS) is defined as the time from the first administration of the investigational drug to the date of first documented disease progression or death from any cause.

On the analysis date or at the end of the study (i.e., cutoff date), if a patient has not experienced disease progression or death, the last adequate tumor assessment prior to the cutoff date or prior to the anti-tumor treatment date will be censored. If a patient begins a new antitumor treatment (including a new chemotherapy regimen or radiotherapy), the censoring date is the last adequate tumor assessment date, which occurs prior to the start of treatment or the cutoff date, whichever comes first. If disease progression or death is recorded after a single missing tumor assessment, the actual event date of disease progression / death will be used as the PFS event date as a preset value. If disease progression or death is recorded after ≥2 missing tumor assessments, the PFS time for these patients will be censored at the last adequate tumor assessment date where no disease progression occurred.

When no baseline tumor assessment is available, the patient's first medication date is considered the PFS censoring date.

Objective Response Rate (ORR)

The Objective Response Rate (ORR) is defined as the proportion of patients with the best overall response of CR or PR (as assessed by RECIST version 1.1 or mRECIST).

In calculating ORR, partial and complete responses prior to any other antitumor treatment are counted as responses, regardless of how many assessments were missed before the response.

According to RECIST version 1.1, when the best overall response of patients is classified as 'unknown' or 'unassessed' in estimating ORR, it is considered non-response.

Patients who experienced disease progression and continued to receive the study treatment after disease progression are considered to have disease progression at the time it is determined. In the calculation of ORR and other efficacy metrics, they are all recorded as disease progression.

Disease Control Rate (DCR)

Disease Control Rate (DCR) is defined as the proportion of patients achieving CR, PR, or SD based on RECIST version 1.1 or mRECIST.

Duration of Response (DOR)

The time from the first recorded objective response (either CR or PR) confirmed by the investigator to the first recorded confirmed objective disease progression or death for any reason, with the earlier of the two times being used. Only patients who achieved objective response are included in the calculation of DOR. The handling principles for events and censoring refer to PFS.

Patient quality of life

Data on patient function, disease-related symptoms, health-related quality of life, and health status were collected using the EORTC QLQ-C30 and EORTC QLQ–HCC18 scales. Descriptive statistics (such as means and medians) were used to summarize the scores of the EORTC QLQ-C30 and EORTC QLQ–HCC18 scales at each planned assessment time point. Additionally, changes in scores from baseline in each domain were summarized at each assessment. Patients with assessable baseline scores and at least one assessable score after baseline during the treatment period could be included in the analysis of changes from baseline.

9.3.5.2 Analysis of the primary efficacy endpoint

The primary efficacy indicators of this study are progression-free survival and overall survival assessed by BICR, with inter-group comparison as a superiority test:

For the PFS and OS endpoints, the null hypothesis H0: HR≥1; alternative hypothesis H1: HR<1

Controlling the overall I Type I error rate at 0.05 two-sided significance level, first conduct a two-sided significance level =0.05 test for PFS; if PFS cannot reject the null hypothesis, the trial ends; If PFS rejects the null hypothesis, continue to use 0.05 two-sided significance level to test OS, conduct an interim analysis of OS at a two-sided significance level =0.0028, and a final analysis of OS at a two-sided significance level =0.049. If either of the efficacy endpoints, PFS or OS, is statistically significant, then superiority is established statistically. In the FAS population, the median PFS and OS and their 95% confidence intervals for both groups were estimated using the Kaplan-Meier method, and survival curves for PFS and OS were plotted. The differences in PFS and OS between the two treatment groups were compared using a stratified log-rank test; if the p-value for the inter-group comparison is <0.05, the difference is considered statistically significant. A COX proportional hazards model was used to estimate the hazard ratio (experimental group/control group) and two-sided 95% CI, with variables in the model including: group and random stratification factors. If the upper limit of the 95% CI for the hazard ratio comparing to the control group is less than 1, then the superiority of the experimental group relative to the control group is established.

9.3.5.3 Analysis of secondary efficacy endpoints

The secondary efficacy endpoints of this study focus on OS beyond 1 year, 1.5 years, and 2 years survival rates (OSR), objective response rate (ORR), investigator-assessed progression-free survival (PFS), duration of response (DOR), disease control rate (DCR), quality of life, and biomarker subgroup analysis.

The overall survival rates at 1 year, 1.5 years, and 2 years were summarized using the Kaplan-Meier method.

For the analysis of ORR/DCR, the incidence rates of the two groups were calculated based on the exact binomial distribution and the 95% confidence interval (CI), and the exact probability (or Chi-square test) method was used to compare the intergroup differences in ORR and DCR.

For patients who achieved objective response (CR or PR), the DOR will be summarized using the Kaplan-Meier method, displayed graphically when applicable. Calculate the median time to event for both groups and the two-sided 95% confidence intervals.

Report descriptive statistics (mean, standard deviation, median, range, and mean's 95% CI) for the absolute scores in each domain of the EORTC QLQ-C30 and EORTC QLQ–HCC18

questionnaires, as well as summarize the changes from baseline using the aforementioned statistics. Plot the average scores and average changes over time for each domain in both groups.

Biomarker subgroup analysis: A comparative analysis of efficacy endpoints in the PD-L1 positive vs PD-L1 negative subgroup populations between the two groups.

9.3.6 Safety analysis

Analyze Phase II and Phase III separately. The analysis of safety evaluation endpoints is conducted within the safety analysis set, including but not limited to the following aspects.

Adverse events

Adverse events are coded using the Chinese version of the Medical Dictionary for Regulatory Activities (MedDRA), and various adverse events are summarized according to System Organ Class (SOC) and Preferred Term (PT).

Calculate the frequency, number of cases, and incidence of TEAE, Grade 3 or higher TEAE, drug-related TEAE, special attention adverse events, severe adverse events, and TEAE leading to treatment discontinuation. Summarize the severity of various TEAEs. A list describing the details of adverse events for each patient, including the type, severity, occurrence and duration, outcome, and the relationship with the trial drug and drug dosage.

Laboratory tests

A descriptive summary of laboratory test indicators at visit points, presented in a cross-tabulation format (based on clinical significance) to summarize the changes in normal and abnormal indicators of patients before and after treatment. A list of patients with normal indicators before treatment and abnormal indicators after treatment, as well as a list of data that were abnormal both before and after treatment.

Vital signs

Descriptive statistics of vital signs examination results and changes compared to baseline.

ECG

Summarize the number and percentage of patients with abnormal ECG findings, and present the changes in normal and abnormal indicators before and after treatment in a cross-tabulation format (based on clinical significance). List the data of patients who were normal before treatment and abnormal after treatment, as well as those who were abnormal both before and after treatment. This includes a list of ECG assessments for patients with at least one abnormal finding.

9.3.7 Pharmacokinetic analysis

Pharmacokinetic parameters were calculated using the Phoenix WinNonlin non-compartmental model method, including but not limited to AUC_{0-t}, $AUC_{0-\infty}$, C_{max}, C_{min}, CL, Vz, and $t_{1/2}$. Descriptive statistical analysis of the PK concentrations and PK parameters of SCT-I10A and SCT510 will be conducted to describe the pharmacokinetic characteristics of SCT-I10A and SCT510 in patients with advanced hepatocellular carcinoma, including descriptive statistical analysis of steady-state trough concentrations.

9.3.8 Immunogenicity analysis

Summarize the proportion of patients with antibodies that appeared during the study period to evaluate the immunogenicity of SCT-I10A combined with SCT510 in patients with advanced hepatocellular carcinoma.

9.3.9 Combination therapy

Coding will be performed using WHO DD, listing concomitant medications and significant non-drug treatments by patient before and after treatment, summarized according to ATC terminology (Anatomical Therapeutic Chemical Classification System).

9.4 Mid-term analysis

The Phase III portion of this study conducts two tests for OS, namely a mid-term analysis and a final analysis. This study is designed to strictly control the Type I error rate at a two-sided significance level of 0.05, with the primary analysis of PFS conducted when the estimated number of PFS events reaches at least 252, while a mid-term analysis of OS will be conducted when the required number of OS events (approximately 125) is expected to be observed at 50%. If the null hypothesis for PFS is rejected, the entire testing level will be transferred to OS, and the mid-term analysis efficacy will be judged using the approximate O’Brien Fleming boundary values of the Lan-DeMets α spending function, with a two-sided detection level of 0.0028 for the OS mid-term analysis and a two-sided testing level of 0.049 for the OS final analysis.

9.5 Multiplicity considerations

In this study, the PFS and OS analyses were conducted using a fixed-sequential procedure; after rejecting the null hypothesis for PFS, the significance level was carried over to OS, thus this step does not require multiplicity adjustment.

A single interim analysis will be conducted at the OS endpoint. The boundaries for the interim analysis are determined by approximating the O'Brien-Fleming boundary using the Lan-Demets method. If the number of OS events occurring during the interim analysis differs from the planned number in the actual study, adjustments to the allocation of Type I error will be made, following the Lan-Demets method to approximate the O'Brien-Fleming boundary based on the actual event count. The strategies and methods for multiplicity analysis will be described in detail in the statistical analysis plan.

9.6 Other analyses

Sensitivity analysis and stratified (subgroup) analyses will be detailed in the SAP.

9.7 Independent Data Monitoring Committee (IDMC)

An independent data monitoring committee (IDMC) composed of external experts unrelated to this clinical study will be established to conduct interim analyses and monitor safety data during the study.

The IDMC will recommend whether to continue, pause, or revise the study protocol based on the interim analysis results and safety considerations.

The responsibilities and specific operational procedures of the IDMC will be detailed in the IDMC charter.

10 Ethics

10.1 Ethics Committee

Before the study begins, the investigator is responsible for submitting the clinical research protocol, informed consent form, and information materials provided to patients (such as recruitment advertisements, patient compensation plans, etc.) to the ethics committee for approval, in order to obtain an independent approval document for conducting the clinical research. The approval document from the ethics committee must be submitted to the investigator in written form, after which the investigator will provide a copy of the approval document to the sponsor. The approval documents from the ethics committee must include a list of all committee members involved in the discussion of the approval documents and their respective professional backgrounds.

During the clinical research process, any issues related to the safety of the clinical study, such as changes to the clinical study protocol or the informed consent of patients, as well as serious adverse events during the clinical study, must be reported to the ethics committee in a timely manner. The conclusion or premature termination of the clinical study must also be reported to the ethics committee in a timely manner.

10.2 Informed consent of patients

The rights, safety, and physical and mental health of patients are the most important considerations and should take precedence over scientific and social interests. Researchers must clearly and comprehensively inform each patient, or their guardian in the case that the patient is unable to provide consent, about the nature, purpose, relevant procedures, expected duration, potential risks and benefits, and any discomfort that may arise from this study in the informed consent. Each patient must be aware that participation in this study is voluntary, and he / he can withdraw from the study and revoke consent at any time without affecting his / her subsequent treatment and relationship with the treating physician. The informed consent form should be presented in a standard written format and should use non-technical language as much as possible. Each informed consent form must include all the relevant content mentioned above, as well as a voluntary statement. The informed consent form must be submitted for approval by the relevant ethics committee.

After the researcher has explained the basic content of the study and has ensured that the patients understand the purpose of the study, each patient participating in the study should be

asked to sign their name and date on the informed consent form. Patients should carefully read and consider their statement before signing their name and date, and they should receive a copy of the signed document. Before starting any procedures related to the clinical study, informed consent from the patients must be obtained, including consent for any screening processes conducted to determine the patients' eligibility to participate in this study. Without obtaining informed consent, patients cannot enter the study.

For those patients who are unable to express informed consent, their guardians may act on their behalf to provide informed consent. The signed original informed consent form must be properly stored by the investigator and should also be recorded in the EDC and relevant original research records.

10.3 Confidentiality of patient information

The investigator is responsible for maintaining the anonymity of the patients. Patients can only be identified in case report forms or other documents using uppercase letters, numbers, and / or codes, and not by their names. Researchers must keep records of the patient enrollment forms that contain patient codes, names, and home addresses. Researchers must strictly maintain the confidentiality of documents that can reveal the identity of the patients.

11 Management of the study

The conduct of this study will comply with the standard operating procedures of the sponsor and the contract research organization (CRO), which are designed to ensure adherence to the guidelines of Good Clinical Practice (GCP) for drug clinical trials. The investigator will agree to adhere to the instructions and processes outlined in the protocol upon signing it, and will comply with the principles of Good Clinical Practice applicable to this protocol as well as all regulations governing medical research in China.

11.1 Sponsor

The sponsor of this study is Sinocelltech Ltd.

11.2 Investigator

Investigator responsibilities:

1) Before the patients are enrolled, the investigator should explain the significance of the study to the enrollees or their family members, obtain their consent, and have them sign the informed consent form;
2) In the event of adverse events, the researchers must investigate the causes and take appropriate actions, while also reporting to the study leader. In the event of a patient's death, the investigator is responsible for providing the patient's tissue pathology and other relevant information;
3) Data must be recorded in the medical records and EDC in a truthful, accurate, complete, timely, and legal manner;
4) Cooperate with the regular inspections conducted by the clinical monitors assigned by the sponsor;
5) Complete retention of laboratory test records, clinical records, and the original medical records of the patients;
6) Follow-up on severe adverse events.

11.3 Clinical monitoring

The monitoring plan will detail this process. Investigators must comply with the requirements to allow monitoring, auditing, and inspection of clinical, laboratory, and pharmacy

facilities to ensure compliance with Good Clinical Practice (GCP) and laboratory quality management standards. The clinical trial monitor is the primary contact between the sponsor and the investigator. Monitors must adhere to Good Clinical Practice (GCP) and Standard Operating Procedures (SOP), visiting research sites regularly or as needed to conduct clinical monitoring, oversee the progress of clinical trial activities, check and confirm the recording and reporting of all data, ensure the accuracy and completeness of EDC entries, and ensure consistency with original data, guaranteeing that the clinical trial is conducted according to the clinical trial protocol. Investigators should actively cooperate with the monitor's work. The specific responsibilities of the monitor include:

1) Confirm before the trial that the trial undertaking unit has appropriate conditions, including staffing and training, that laboratory equipment is complete and functioning well, that various examination conditions related to the trial are available, that there is an estimated sufficient number of patients, and that the participating researchers are familiar with the requirements of the trial protocol;
2) During the trial, monitor the researchers' adherence to the trial protocol, confirm that informed consent has been obtained from all patients prior to the trial, understand the enrollment rate of patients and the progress of the trial, and confirm the eligibility of enrolled patients;
3) Confirm that all data records and reports are accurate and complete. All case report forms are entered correctly and are consistent with the original data. All errors or omissions have been corrected or noted, and signed and dated by the investigator. Changes in dosage, treatment modifications, combination therapy, concurrent diseases, loss to follow-up, and missed examinations for each patient should be confirmed and recorded. Verify whether the withdrawal and loss to follow-up of enrolled patients have been explained in the original records and EDC;
4) Confirm that all adverse events are recorded, and that severe adverse events are reported and documented within the specified timeframe. Verify that the investigational drugs are supplied, stored, distributed, and retrieved in accordance with relevant regulations, and that appropriate records are maintained;
5) Assist the investigator with necessary notifications and applications, and report trial data and results to the sponsor;

6) Clearly and truthfully record the visits that the investigator failed to conduct, the tests that were not performed, the examinations that were not done, and whether corrections were made for errors and omissions;
7) Complete a written monitoring report after each visit, which should state the monitoring date, time, name of the monitor, findings of the monitoring, etc.

11.4 Medical Monitoring

The specific content of the medical monitoring work for this study will be specified in a separate Medical Monitoring Plan (MMP), including the concept of medical monitoring, key participants and their responsibilities, the process of medical monitoring for this study, and important deadlines. The preparation of the MMP will begin from the finalization of the study protocol and will be completed no later than before the enrollment of the first patient. It will be further updated as needed during the subsequent medical monitoring work to ensure the scientific and rational conduct of the medical monitoring for this study. The contact information and details of the medical monitors and other contacts will be listed in the study center documents.

11.5 Audit

The study will be organized, conducted, and reported in accordance with the protocol, sponsor, and CRO's SOP requirements. In ICH E6, quality assurance (QA) is defined as 'all those planned and systematic actions intended to ensure that the trial is conducted and the data are generated, recorded, and reported in compliance with Good Clinical Practice (GCP) and applicable regulatory requirements.' Sponsor QA activities will be conducted according to the provisions of the study audit plan. Section 5.19.3(b) of ICH E6 states that the development of the audit plan and trial audit processes should be guided by the importance of the trial in the submission materials to the competent authorities, the number of patients in the trial, the type and complexity of the trial, the risk level of the trial patients, and any identified issues. QA activities may be outsourced to a CRO or an independent consulting firm. Researchers are required to support audit activities, attend as requested by the auditors, and allow auditors to directly access original data/documents.

Qualified regulatory authorities (CA) and/or authorized third parties may also conduct inspections (during or after the study). If a CA requests an inspection, the researcher must immediately notify the sponsor upon receiving this request.

11.6 Recording and preservation of research data

In accordance with GCP principles, researchers should retain all detailed original documents of patients and record information regarding trial progress, medication, laboratory test data, safety data, and efficacy assessments in the case report forms. The recorded data should be truthful, accurate, complete, timely, and clear. Original documents, medical records, etc. should be clear, detailed, and easily identifiable by personnel participating in this clinical trial.

Case report forms and original documents may only be modified by the investigator. Any modifications to case report forms and original documents must not obliterate the original data. The correct method of modification is to draw a single line through the original data, then write the modified data next to it, and sign with the date and the initials of the person making the modification.

The investigator should ensure that the data is preserved in its entirety. According to the GCP principles in our country, the investigator should retain clinical research data for five years after the study concludes. The sponsor should retain clinical research data for five years after the investigational drug is approved for marketing.

If the principal investigator changes jobs, retires, or no longer fulfills their research responsibilities, they must notify the sponsor and the CRO in writing to develop appropriate measures regarding changes to the trial documentation.

12 Registration and publication of research results

The sponsor will report the results of this study in the clinical research report, including the case report form data from all clinical research centers participating in this study. The analysis results obtained after the publication of the clinical research report will be included in a separate report, and there is no need to modify the clinical research report. When publishing the results, the identities of the patients will not be disclosed. All research-related works and data protected by copyright (except for any literature published by the researchers as mentioned below) shall be the property of the sponsor and shall be treated as the author and copyright owner.

If researchers wish to publish relevant information about the study, they must obtain the sponsor's consent and provide a manuscript copy to the sponsor for review at least 60 days prior to submission or presentation. For abstracts, conference posters, or other materials (such as invited talks or guest lectures), expedited review can be arranged. At the written request of the sponsor, researchers are required to retain up to an additional 60 days to allow for the completion of patent applications. If issues arise regarding scientific integrity or compliance management, the sponsor will review these issues with the researchers. The sponsor may not compel modifications to the scientific content and has no authority to delete information.

13 References

[1]QIN S E A. Efficacy and Safety of Atezolizumab + Bevacizumab vs Sorafenib in Chinese Patients With Unresectable HCC in the Phase III IMbrave150 Study[J]. EASL Liver Cancer Summit, 2020.

[2]CHEN W, ZHENG R, BAADE P D, et al. Cancer statistics in China, 2015[J]. CA: A Cancer Journal for Clinicians, 2016,66(2): 115-132.

[3]BRAY F, FERLAY J, SOERJOMATARAM I, et al. Global cancer statistics 2018: GLOBOCAN estimates of incidence and mortality worldwide for 36 cancers in 185 countries[J]. CA Cancer J Clin, 2018,68(6): 394-424.

[4] ZHOUKQ. Guidelines for Diagnosis and Treatment of Primary Liver Cancer - National Health Commission [J]. 2020: 75.

[5]YANG J D, HAINAUT P, GORES G J, et al. A global view of hepatocellular carcinoma: trends, risk, prevention and management[J]. Nat Rev Gastroenterol Hepatol, 2019,16(10): 589-604.

[6]M J. Sorafenib in Advanced Hepatocellular Carcinoma[J]. The New England Journal of Medicine, 2008.

[7]CHENG A. Efficacy and safety of sorafenib in patients in the Asia-Pacificregion with advanced hepatocellular carcinoma: a phase III randomised, double-blind, placebo-controlled trial[J]. The Lancet Oncol, 2009.

[8]KUDO M, FINN R S, QIN S, et al. Lenvatinib versus sorafenib in first-line treatment of patients with unresectable hepatocellular carcinoma: a randomised phase 3 non-inferiority trial[J]. The Lancet, 2018,391(10126): 1163-1173.

[9]Hepatobiliary Cancers，NCCN, 2019v2.[J].

[10]LIU X, QIN S. Immune Checkpoint Inhibitors in Hepatocellular Carcinoma: Opportunities and Challenges[J]. The Oncologist, 2019,24(S1).

[11]YAU T. CheckMate 459: A randomized, multi-center phase III study of nivolumab (NIVO) vs sorafenib (SOR) as first-line (1L) treatment in patients (pts) with advanced hepatocellular carcinoma (aHCC).[J]. Annals of Oncology, 2019.

[12]ZHU A X, FINN R S, EDELINE J, et al. Pembrolizumab in patients with advanced hepatocellular carcinoma previously treated with sorafenib (KEYNOTE-224): a non-randomised, open-label phase 2 trial[J]. The Lancet Oncology, 2018,19(7): 940-952.

[13]FINN R. Results of keynote240：Phase 3 study of Pembrolizumab vs best supportive care for second-line therapy in advanced hepatocellular carcinoma[J]. ASCO Annual Meeting,

2019.

[14]HUANG Y, CHEN X, DIKOV M M, et al. Distinct roles of VEGFR-1 and VEGFR-2 in the aberrant hematopoiesis associated with elevated levels of VEGF[J]. Blood, 2007,110(2): 624-631.

[15]SHRIMALI R K, YU Z, THEORET M R, et al. Antiangiogenic Agents Can Increase Lymphocyte Infiltration into Tumor and Enhance the Effectiveness of Adoptive Immunotherapy of Cancer[J]. Cancer Research, 2010,70(15): 6171-6180.

[16]CHEN D S, HURWITZ H. Combinations of Bevacizumab With Cancer Immunotherapy[J]. Cancer journal (Sudbury, Mass.), 2018,24(4): 193-204.

[17] Niu Zhicheng, He Dongwei, Wang Zhiyu. Research progress on the combination of anti-angiogenic drugs and immune checkpoint inhibitors in the treatment of malignant tumors [J]. Chinese Journal of Tumor Biological Therapy, 2019, 26(09): 1012-1018.

[18]HSU D C. Randomised Efficacy and Safety Results for Atezolizumab + Bevacizumab in Patients With Previously Untreated, Unresectable Hepatocellular Carcinoma[J]. ESMO ASIA, 2019.

[19]CHENG A. Atezolizumab + bevacizumab vs sorafenib in patients with unresectable hepatocellular carcinoma: Phase 3 results from IMbrave150[J]. ESMO ASIA, 2019.
