# Supplementary material for: Finotonlimab (PD-1 inhibitor) plus bevacizumab (bevacizumab biosimilar) as first-tier therapy for late-stage hepatocellular carcinoma: a randomized phase 2/3 trial
Source: Signal Transduct Target Ther. 2025 Aug 6;10:249. doi: 10.1038/s41392-025-02333-5 (PMC12329032; doi:10.1038/s41392-025-02333-5)
Supplement: Supplementary file 3 — Statistical analysis plan of a clinical trial [file 41392_2025_2333_MOESM3_ESM.pdf]

Evaluation of SCT-I10A Combined with SCT510 Versus Sorafenib in First-line Treatment of Advanced Hepatocellular Carcinoma: A Multi-center, Randomized, Open-Label, Phase II/III Trial

Protocol No. SCT-I10A-C301

Statistical Analysis Plan

(SAP)

Version: V1.0/2023-11-15

SinoCellTech Ltd.

The ownership of all information contained in this document belongs to SinoCellTech Ltd., and is provided only for review by the investigator, co-investigators, ethics committees and supervisory and regulatory authorities, and other relevant organizations. Without the written approval of SinoCellTech Ltd., it is strictly prohibited to disclose any information to third parties unrelated to this study, except for necessary explanations when signing the Informed Consent Form with the subjects who may participate in this study.

SIGNATURE PAGE

Author

______________________________ ______________

Huo Su — Date (yyyy-mm-dd)

Study Statistician, Statistics Manager

SinoCellTech Ltd.

Approved by

______________________________ ______________

Dongfang Liu — Date (yyyy-mm-dd)

Director of Statistics

SinoCellTech Ltd.

Approved by

______________________________ ______________

Yan Wang — Date (yyyy-mm-dd)

Medical Director

SinoCellTech Ltd.

TABLE OF CONTENTS

TABLE OF CONTENTS 3

LIST OF ABBREVIATIONS 6

1 INTRODUCTION 8

2 TRIAL OVERVIEW 8

2.1 Study Objectives 8

2.1.1 Phase II Single-arm Study 8

2.1.2 Phase III Randomized Controlled Study 9

2.2 Design Type 9

2.3 Type of Control 10

2.4 Comparison Type 10

2.5 Randomization Method and Implementation 10

2.6 Blinding Method and Measures 11

3 STUDY ENDPOINTS 11

3.1 Phase II Single-arm Study 11

3.1.1 Safety Assessment Endpoints 11

3.1.2 Other Assessment Endpoints 11

3.2 Phase III Randomized Controlled Study 12

3.2.1 Primary Endpoints 12

3.2.2 Secondary Endpoints 12

4 SAMPLE SIZE ESTIMATION 13

5 ANALYSIS SET 14

6 STATISTICAL ANALYSIS METHODS 14

6.1 General Considerations 14

6.2 Subject Information 15

6.2.1 Analysis of Subject Distribution 15

6.2.2 Follow-up Time Analysis 15

6.2.3 New Anticancer Therapy 15

6.3 Demographic Information and Baseline Characterization 16

6.3.1 Demographics 16

6.3.2 Hepatocellular Carcinoma-related Baseline Characteristics 16

6.3.3 Medical History and Allergy History *16*
6.3.4 Immunology, HCV/RNA and HBV-DNA Testing *16*
6.4 Efficacy Analysis 17
6.4.1 Primary Endpoint Analysis *17*
6.4.2 Secondary Endpoint Analysis *22*
6.4.3 Subgroup Analysis *27*
6.4.4 Integrated Summary of Effectiveness *28*
6.5 Safety Analysis 28
6.5.1 Prior and Combined Drug/Non-drug Therapy *28*
6.5.2 Study Drug Exposure and Compliance *29*
6.5.3 Adverse Events *30*
6.5.4 Laboratory Tests *33*
6.5.5 Vital Signs, Physical Examination, and Other Safety Assessments *33*
6.6 PK and Immunogenicity Analysis 35
6.7 Treatment of Missing Data 35
6.7.1 Missing Date *35*
6.7.2 Efficacy/Safety Indicator Data Missing *36*

7 INTERIM AND FINAL ANALYSIS OF OS 36

8 MAJOR CHANGES IN SAP RELATIVE TO THE PROTOCOL 39

9 REFERENCES 40

Table of Contents

Table 1.1 Document Revision Record.. 8
Table 6.1 PFS primary analysis and sensitivity analysis censoring rules 19
Table 6.2 Best overall response (BOR) derivation rules.. 24
Table 6.3 EORTC QLQ-C30 and QLQ-HCC18 Dimension Composition and Criteria Scoring Methods 26
Table 7.1 OS Interim and Final Analysis Boundaries 38

LIST OF ABBREVIATIONS

English Abbreviation	Full name in Chinese
12-ECG	12-Lead Electrocardiogram
ADA	Anti-drug Antibody
ADR	Adverse Drug Reaction
AE	Adverse Event
AESI	Adverse Events of Special Interest
AFP	Alpha-fetoprotein
AFT	Accelerated Failure Time Model
ATC	Anatomical Therapeutic Chemica
BCLC	Barcelona Clinical Liver Cancer Staging
BICR	Blinded Independent Central Review
BID	Twice Daily
BLA	Biologic License Application
BOR	Best Overall Response
CI	Confidence Interval
CR	Complete Response
CRF	Case Report Form
CT	Computed Tomography
CTCAE	Common Terminology Criteria for Adverse Events
DCR	Disease Control Rate
DOR	Duration of Response
EAIR	Exposure Adjusted Incidence Rate
ECOG	Eastern Cooperative Oncology Group
eCRF	Electronic Case Report Form
EOS	End of Study
EOT	End of Treatment
FAS	Full Analysis Set
FDA	Food and Drug Administration
FSI	First Subject Enrollment
FWER	Familywise Error Rate
GCP	Good Clinical Practice
HbsAg	Hepatitis B Surface Antigen
HBV	Hepatitis B Virus
HCC	Hepatocellular Carcinoma
HCV	Hepatitis C Virus
HIV	Human Immunodeficiency Virus
HR	Hazard Ratio
ICH	The International Council for Harmonisation of Technical Requirements of Pharmaceuticals for Human Use

**English Abbreviation**	**Full name in Chinese**
IDMC	Independent Data Monitoring Committee
irAE	Immune-related Adverse Event
ITT	Intent-to-treat
IWRS	Interactive Web Response System
MedDRA	Medical Dictionary for Regulatory Activities
mRECIST	Modified Response Evaluation Criteria in Solid Tumors
N/A	Not Applicable
NAb	Neutralizing antibody
NE	Not Evaluable
NMPA	National Medical Products Administration
ORR	Objective Response Rate
OS	Overall Survival
OSR	Overall Survival Rate
PD	Disease Progression
PFS	Progression-free Survival
PK	Pharmacokinetics
PPS	Per-protocol Set
PR	Partial Response
PRO	Patient-reported Outcome
PT	Preferred Terms
RECIST	Response Evaluation Criteria in Solid Tumors
RES	Response-evaluable Analysis Set
RPSFT	Rank Preserving Structural Failure Time
SAE	Serious Adverse Event
SAP	Statistical Analysis Plan
SD	Disease Stabilization
SOC	System Organ Class
SS	Safety Analysis Set
TEAE	Treatment Emergent Adverse Event

1 INTRODUCTION

This statistical analysis plan follows ICH guidelines (e.g., ICH E9 Statistical Principles for Clinical Trials), NMPA guidelines (e.g., Guideline on Data Management Plan and Statistical Analysis Plan of Drug Clinical Trials, Statistical Principles for Clinical Trials of Medicinal Products), and is based on the study protocol (SCT -I10A-C301 V2.0 /2021-04-20) and CRF (V2.2/2022-05-30), to provide a detailed description of the content and methods of their statistical analysis. To avoid inflation of the Familywise Error Rate (FWER) and minimize operational bias, the SAP will be finalized before data blinding, and the finalized SAP will be not updated after blinding.When the analytical principles specified in the SAP remain unchanged, the presentation of the specific analytical content may be adjusted to take into account the main purpose of the study, regulatory requirements, and regulations.

Table 1.1 Document Revision Record

SAP Version/Date	Author	Revision Summary
V1.0/2023-11-15	Huo Su	N/A

2 Trial Overview

This study is a multi-center, randomized, open-label phase II/III operated seamless design with two parts: a phase II single-arm part and a phase III randomized controlled part, with patients from phase II excluded from the confirmatory analysis of phase III.

2.1 Study Objectives

2.1.1 Phase II Single-arm Study

2.1.1.1 Primary Objective

To evaluate the safety and tolerability of SCT-I10A in combination with SCT510.

2.1.1.2 Secondary Objective

1) To evaluate the objective response rate (ORR), duration of response (DOR), disease control rate (DCR), progression-free survival (PFS), and overall survival (OS) of subjects treated with SCT-I10A in combination with SCT510 as assessed by the investigator per RECIST v1.1 criteria;

2) To evaluate the pharmacokinetic characteristics of SCT-I10A in combination with SCT510;
3) To evaluate the immunogenicity of SCT-I10A and SCT510.

2.1.2 Phase III Randomized Controlled Study

2.1.2.1 Primary Objective

To evaluate progression-free survival (PFS) and overall survival (OS) as assessed by Blinded Independent Central Review (BICR) based on RECIST v1.1 in subjects treated with SCT-I10A in combination with SCT510 compared to sorafenib.

2.1.2.2 Secondary Objective

1) 1-year, 1.5-year, and 2-year overall survival rate (OSR) of subjects treated with SCT-I10A in combination with SCT510 compared to sorafenib;
2) Investigator-assessed progression-free survival (PFS) of subjects treated with SCT-I10A in combination with SCT510 compared to sorafenib based on RECIST v1.1 criteria;
3) BICR and the investigator assessed the objective response rate (ORR), duration of response (DOR), and disease control rate (DCR) of SCT-I10A in combination with SCT510 compared to sorafenib in subjects based on the RECIST v1.1 standard;
4) BICR evaluated the progression-free survival (PFS), objective response rate (ORR), duration of response (DOR), and disease control rate (DCR) of SCT-I10A combined with SCT510 compared to sorafenib in subjects based on the mRECIST standard;
5) evaluated the safety of subjects treated with SCT-I10A combined with SCT510;
6) To evaluate the quality of life (EORTC QLQ-C30 and EORTC QLQ-HCC18 scales) of subjects treated with SCT-I10A in combination with SCT510 compared to sorafenib;
7) Evaluate the steady-state trough concentrations and pharmacokinetic characteristics of SCT-I10A and SCT510 in patients with advanced hepatocellular carcinoma;
8) Evaluate the immunogenicity of SCT-I10A and SCT510;
9) Evaluate the correlation between PD-L1 expression levels and efficacy and prognosis.

2.2 Design Type

This study adopts a multi-center, randomized, open-label phase II/III operation seamless

design, the safety and efficacy of the phase II single-arm study will be analyzed before the 3rd dose (completion of the first efficacy evaluation) of the last subject in the phase II study, and after preliminary confirmation of the safety and efficacy, it will enter into the phase III study, and the subjects in phase II will not be included in the confirmatory analysis of phase III.

Phase III is a randomized, controlled study that plans to enroll approximately 342 subjects and randomly assign them 2:1 to the SCT-I10A+SCT510 group (experimental group) or the sorafenib group (control group). The BICR is set to assess progression-free survival (PFS) based on RECIST v1.1 and overall survival (OS) as the dual primary endpoints. The Familywise Error Rate (FWER) is controlled using the fixed-sequential procedure, and after rejecting the null hypothesis for PFS, the two-sided test level of 0.05 will be passed to OS. It is planned that for the final analysis of PFS , approximately 252 PFS events provide 85% power; OS performs an interim analysis (when the number of PFS events reaches 252) and a final analysis. The OS final analysis requires 253 OS events, also providing 85% power.

It is expected that a final analysis of PFS will be performed when approximately 252 PFS events have been observed. if the null hypothesis for PFS is not rejected, the trial will be terminated. On the contrary, if the test result of PFS is significant, an interim analysis of OS will be performed at the time of the final analysis of PFS, at which time it is expected that approximately 127 (50% of 253 total expected) deaths will have been observed. Type I error will be controlled using the O'Brien-Fleming type alpha spending function (approximated by the Lan-DeMets method), and the superiority boundary value for the interim and final OS analyses will be determined based on the proportion of events that actually occur.

2.3 Type of Control

This phase III stage of the study uses sorafenib as a positive control and the experimental group is SCT-I10A combined with SCT510, to evaluate its efficacy and safety compared to sorafenib in the first-line treatment of advanced hepatocellular carcinoma.

2.4 Comparison Type

The phase III part is a superiority design that aims to verify whether the therapeutic effect of SCT-I10A in combination with SCT510 is superior to sorafenib.

2.5 Randomization Method and Implementation

This trial uses stratified block randomization in the phase III part. Subjects who have

signed the informed consent form and meet the inclusion and exclusion criteria will be randomly assigned to the SCT-I10A+SCT510 group (experimental group) or the sorafenib group (control group) in a 2:1 ratio using a central randomization system (Interactive Web Response System, IWRS). Randomization stratification factors include:

- ECOG performance status (0 vs. 1);
- baseline AFP level (<400 ng/ml vs. ≥400 ng/ml)
- macrovascular invasion and/or extrahepatic metastasis (no vs. yes)

Patients will be considered to have failed randomization will be withdrawn from the study if they did not receive study medication within 3 days of randomization.

2.6 Blinding Method and Measures

This study is an open-label, to minimize the risk of bias associated with open-label status and to maintain data integrity, especially the monitoring of the trial data including the interim analysis, an independent data monitoring committee (IDMC) consisting of external experts not related to this clinical study will be established in this study.

The final analysis of PFS and the interim analysis of OS (collectively referred to as the interim analysis) will be conducted by the IDMC and an independent statistical team. After the interim analysis, if the IDMC determines that the study has met the primary endpoint, the sponsor may consider setting up a dedicated unblinded team to apply for the Biologic License Application (BLA). Meanwhile, the team responsible for long-term survival follow-up will remain blinded, and there will be a "firewall" between the blinded and unblinded holders until the BLA is approved by the regulatory authority.

3 Study Endpoints

3.1 Phase II Single-arm Study

3.1.1 Safety Assessment Endpoints

Treatment Emergent Adverse Event (TEAE); clinical laboratory; ECOG score; vital signs; 12-ECG; thyroid function; and physical examination.

3.1.2 Other Assessment Endpoints

3.1.2.1 Efficacy Assessment Endpoints

The investigators assessed the objective response rate (ORR), duration of response (DOR), disease control rate (DCR), progression-free survival (PFS), and overall survival (OS) of the subjects based on the RECIST v1.1 criteria.

3.1.2.2 PK Evaluation Endpoints & Immunogenicity Assessment Endpoints

PK and immunogenicity evaluation endpoints will be detailed in a separate analysis plan.

3.2 Phase III Randomized Controlled Study

3.2.1 Primary Endpoints

The dual primary endpoints of this phase III study part are progression-free survival (PFS) and overall survival (OS) as assessed by Blinded Independent Central Review (BICR) according to RECIST v1.1.

3.2.2 Secondary Endpoints

3.2.2.1 Other Efficacy Assessment Endpoints

- 1-year, 1.5-year, and 2-year survival rates (OSR);
- Progression-free survival (PFS) evaluated by the investigator based on RECIST v1.1 criteria;
- Objective response rate (ORR), duration of response (DOR), and disease control rate (DCR) evaluated by BICR and investigators based on RECIST v1.1 criteria;
- Progression-free survival (PFS), objective response rate (ORR), duration of response (DOR), and disease control rate (DCR) evaluated by BICR based on mRECIST criteria;
- Changes in subjects' quality of life (EORTC QLQ-C30 and EORTC QLQ-HCC18 scales);
- Evaluation of the correlation of PD-L1 expression levels with efficacy and prognosis (see section 6.4.3 Subgroup analysis).

3.2.2.2 Safety Assessment Endpoints

Treatment Emergent Adverse Event (TEAE); clinical laboratory; ECOG score; vital signs; 12-ECG; thyroid function; and physical examination.

3.2.2.3 PK Evaluation Endpoints

PK evaluation endpoints will be detailed in a separate analysis plan.

3.2.2.4 Immunogenicity Evaluation Endpoints

Immunogenicity evaluation endpoints will be detailed in a separate analysis plan .

4 Sample Size Estimation

342 patients are planed to enrolled in the Phase III part and randomized 2:1 to either the SCT-I10A+SCT510 group (experimental group) or the sorafenib group (control group). One PFS analysis is planned at the occurrence of 252 PFS events to provide 85% power to detect an HR of 0.67 favoring finotonlimab+SCT510 compared with sorafenib; and two OS analyses (one interim and a final) are planned at the occurrence of 127 (50% of 253 total expected) deaths and 253 deaths, also providing a power of 85% to detect an HR of 0.67 favoring finotonlimab+SCT510.

The family-wise type I error rate will be strictly control at two-sided 0.05. Using a fixed-sequential procedure, PFS and OS will be analyzed with a two-sided α of 0.05, respectively. The PFS will be analysed first, if the null hypothesis for PFS is not rejected, the trial will be terminated, if the test result of PFS is significant, the interim analysis of the OS is planned concurrently with the final PFS analysis. The Lan-DeMets spending function will be used to estimate the O'Brien-Fleming boundary for the interim and final analyses of the OS. The superiority boundary values for the OS interim and final analyses will be determined based on the percentage of the actual number of events that occurred (see Section 7 OS Interim and Final Analyses for details).

Sample size estimation is mainly based on the following assumptions:

- PFS and OS time are exponentially distributed;
- The median PFS is 3.6 months in the sorafenib group and 5.4 months in the SCT-I10A+SCT510 group, HR=0.67;
- The median OS is 12 months in the sorafenib group and 17.9 months in the SCT-I10A+SCT510 group, HR=0.67;
- Interim and final analyses of OS will be performed using an O'Brien-Fleming type alpha spending function (approximated by the Lan-DeMets method) to control for the total type I error rate;
- It is expected that 252 PFS events will be observed at month 16 after the first

enrollment and 253 OS events at month 41 after the first enrollment;

- The planned enrollment period is 12 months, with an annualized shedding rate of 5% after enrollment.

5 Analysis Set

Intention-to-treat (ITT) analysis set: Phase II for all enrolled subjects; Phase III for all randomized subjects, according to the group assigned by randomization.

Safety set (SS): includes all subjects who received at least one dose of study medication, grouped according to the actual study medication taken.

Modified Intention-to-treat (ITT) analysis set (Full analysis set, FAS): based on the intention-to-treat (ITT) principle, all enrolled subjects in Phase II and all randomized subjects in Phase III who received at least one dose of study medication.

Response-Evaluable set (RES): a subset of the mITT population that included all subjects with an measurable baseline tumor assessment.

Per-protocol set (PPS): a subset of the mITT population in which subjects are included that generally required the following characteristics: no major protocol deviations that could have affected the main study conclusions. The resolution is finalized by blind data review.

Pharmacokinetic analysis set (PKS): includes all subjects who received at least one dose of study medication and at least one dose of post-treatment blood concentration evaluation data. Specifics will be described and used in a separate analysis plan.

6 Statistical Analysis Methods

6.1 General Considerations

This study used a seamless design with phase II/III operations. Subjects in the phase II trial will be excluded from the confirmatory analysis in phase III. SAS 9.4 or above was used for sample size estimation, statistical decision-making, and analysis. The last non-missing observation before the first administration of the drug is used as the baseline. Unless otherwise specified, the corresponding statistical measures will be used for analysis according to the treatment group and index type. When each statistical measure is used to write a clinical study summary, it can be selectively presented in consideration of regulatory guidelines and clinical considerations.

Statistical description: For continuous variables, the number of cases (N), the number of missing cases (Nmiss), the mean (Mean), the standard deviation (SD), the median (Median),

the minimum value (Min), and the maximum value (Max) will be listed. For categorical variables, the frequency and percentage will be described. For time-to-event variables, the median and its 95% CI can be estimated using the Kaplan-Meier method.

Statistical inference: According to the treatment group and the type of index, the corresponding statistical methods will be used to compare the group or the group, and descriptive statistics, confidence intervals, test statistics and P values for comparisons between groups will be given. In addition to the primary endpoint, which considers the control of the total type I error rate alone, the nominal significance level for the remaining hypothesis tests is 0.05 on both sides, and the two-sided P value is given by default. The P value is retained to 4 decimal places. If the P value is less than 0.0001, it is displayed as "<0.0001"; if the P value is greater than 0.9999, it is displayed as ">0.9999".

6.2 Subject Information

6.2.1 Analysis of Subject Distribution

Based on all subjects who signed the informed consent (screening subjects), summarize the screening results and the reasons for screening failure, describe the enrollment status, the reasons for end of treatment and end of the study and the proportion by group, and list the above information.

Based on all enrolled subjects (ITT), summarize protocol deviations recorded in the study, both overall and by site, and list the specific circumstances of the protocol deviations. Calculate the number and percentage of subjects included in each group in the SS, mITT (FAS), and RES, summarize the reasons for non-inclusion, and list the division of the analysis set.

6.2.2 Follow-up Time Analysis

Based on the mITT (FAS), the follow-up time of the subjects was calculated using the reverse Kaplan-Meier method, which interchanges censoring and events in the OS survival outcome and then performs a K-M estimation. The results of the OS censoring/event determination are the same as those in Section 6.4.1 for the primary endpoint analysis.

6.2.3 New Anticancer Therapy

Based on the mITT (FAS), the follow-up new anticancer therapy of each subject will be summarized, with descriptive statistics by treatment modality.

6.3 Demographic Information and Baseline Characterization

This section will be analyzed based on the mITT (FAS).

6.3.1 Demographics

Demographic information such as age, height, weight, BMI, gender, ethnicity, etc. will be described and counted, and age is treated by both measures and counts (<65 vs. ≥65 years) , and the corresponding subjects will be listed.

6.3.2 Hepatocellular Carcinoma-related Baseline Characteristics

Important baseline characteristics associated with hepatocellular carcinoma will be analyzed descriptively: Disease duration, primary diagnostic modality, macrovascular invasion and/or extrahepatic metastases, presence of extrahepatic metastases, extrahepatic metastatic lesions, presence of macrovascular invasion, AFP level, ECOG score, screening stage BCLC staging, Child-Pugh classification of liver function, history of viral hepatitis, previous localized hepatic therapy, status of the target lesion at baseline, and PD-L1 expression level.

Duration of disease (months) = (time of first drug- date of first diagnosis + 1)/30.4375.

6.3.3 Medical History and Allergy History

Describe the past history and allergy history of the subjects, summarizing the history of previous anticancer drug therapy, previous anticancer non-pharmacological therapy, previous anticancer surgical therapy, previous anticancer radiotherapy therapy, previous anticancer liver localization, and allergy history, respectively. For drug therapy, coded in WHODrug latest version. For non-pharmacological treatments, they will be summarized by SOC and PT in MedDRA latest version.

Describe the subject's past medical history and allergy history, and summarize the past anticancer drug treatment history, past anticancer non-drug treatment history, past anticancer surgical treatment history, past anticancer radiotherapy treatment, past anticancer local liver treatment, allergy history, etc. For drug treatment, code in WHODrug latest version. For non-drug treatment, summarize in MedDRA latest version according to SOC and PT.

6.3.4 Immunology, HCV/RNA and HBV-DNA Testing

Descriptive analysis of baseline testing for the following indicators:

Anti-HIV antibodies, anti-HCV antibodies, hepatitis B five-antigen (HBsAg, HBsAb,

HBeAg, HBeAb, HBcAb), HCV-RNA, HBV-DNA.

6.4 Efficacy Analysis

6.4.1 Primary Endpoint Analysis

6.4.1.1 Statistical Hypothesis

This phase III study part is a superior efficacy trial. The dual primary endpoints are progression-free survival (PFS) and overall survival (OS) assessed using BICR based on RECIST v1.1. The two-sided significance level is α = 0.05.

The null and alternative hypotheses for the PFS and OS analyses can be expressed using the corresponding survival functions:

H_0: The survival function is the same between the two groups, with S_T (t) = S_C(t)

H_1: The survival function is different between the two groups, S_T (t) ≠ S_C (t)

6.4.1.2 PFS Analysis

6.4.1.2.1 PFS Primary Analysis

PFS (months) = (PFS events/censored date – study drug first administration date + 1)/30.4375. For the PFS primary analysis, the rules for censoring are shown in Table 6.1.

The PFS primary analysis is based on the mITT (FAS) and uses progression-free survival (PFS) assessed by BICR based on RECIST v1.1. The PFS events/censored outcome will be summarized. The Kaplan-Meier method will be used to estimate the PFS survival curves for each treatment group, providing the 25th percentile, median, and 75th percentile PFS, and the Brookmeyer-Crowley method will be used to estimate the 95% CI. The Greenwood formula and log-log transformation will be used to calculate the 3-month, 6-month, 9-month, and 12-month PFS rates and their 95% CIs. The Greenwood formula can be used to construct a standard normal distribution, and comparisons between groups will be based on the Z statistic.

The comparison of the PFS survival functions of the two groups will be performed using a stratified log-rank test with the actual randomization stratification results. Stratification factors included ECOG score (0 vs 1), baseline AFP level (<400 ng/ml vs ⩾400 ng/ml), and the presence or absence of macrovascular invasion and/or extrahepatic metastasis (no vs yes), and the significance level is 0.05 on both sides. The study will be failed when the log-rank test did not reject the null hypothesis. The hazard ratio (HR) for PFS and its 95% CI will be calculated using a Cox proportional hazards model based on actual randomization stratification.

6.4.1.2.2 PFS sensitivity analysis

Based on the primary analysis of PFS, sensitivity analyses 1-4 will be taken in order to demonstrate the robustness of the main analysis. Table 6.1 describes the censoring rules for the PFS primary analysis and the 4 sensitivity analyses. Based on the mITT (FAS), sensitivity analyses 1-3 will be done for PFS, PFS (months) = (PFS event/censored date - date of first administration of study drug +1)/30.4375. Based on all randomized subjects in phase III (ITT), sensitivity analyses 4 will be also done for PFS, PFS (months) = (PFS event/censored date - date of randomization +1)/30.4375.

Table6.1 PFS primary analysis and sensitivity analysis censoring rules

Event (PD or death) occurrence	Primary analysis	Sensitivity analysis 1	Sensitivity Analysis 2	Sensitivity Analysis 3	Sensitivity analysis 4
No baseline tumor assessment or no post-treatment, no deaths	**Censored** on the date of first dose of study drug	**Censored** on date of first dose of study drug	**Censored** on date of first dose of study drug	**Censored** on date of first dose of study drug	**Censored** on date of randomization
No PD, no death, and no new anticancer therapy	**Censored** at date of last evaluation of tumor	**Censored** at date of last tumor assessment	**Censored** at date of last tumor assessment	1. Subject completes treatment and **is censored** at the date of the final tumor assessment;<br>2. The subject terminates treatment with the study drug for reasons other than completion of treatment and the decision to end treatment is recorded as an **event** in the Decision to End Treatment Diary.	**Censored** at date of last tumor assessment
No PD, no death, but use of new anticancer therapy	Date of last tumor assessment prior to initiation of new anticancer therapy is recorded as **censored**, no tumor assessment is recorded as **censored** on the date of first dose of drug	Date of last tumor assessment prior to start of new anticancer therapy recorded as **censored**, date of first dose without tumor assessment recorded as **censored**	**Event** on the start date of the new anticancer therapy	**Event** on date of new anticancer therapy initiation	Date of last tumor assessment prior to new anticancer therapy initiation is noted as **censored**, or on date of randomization if no tumor assessment is available
First PD or death not more than two cycles from the last tumor assessment (or date of first administration of study drug in the absence of a last tumor assessment)*	**Event** at PD or date of death	**Event** at PD or date of death	**Event** at PD or date of death	**Event** at PD or date of death	Date of PD or death noted as **event**
First PD or death more than two cycles since the last tumor	The date of the last tumor assessment before	The date of PD or death is recorded as an **event**	The date of the last tumor assessment before	The date of the last tumor assessment before more	The date of the last tumor assessment

Event (PD or death) occurrence	Primary analysis	Sensitivity analysis 1	Sensitivity Analysis 2	Sensitivity Analysis 3	Sensitivity analysis 4
evaluation (or date of first administration of study drug in the absence of a previous tumor evaluation)*	more than two cycles are missing is noted as **censored.**		more than two cycles are missing is noted as **censored.**	than two cycles are missing is noted as **censored.**	before more than two cycles are missing is noted as **censored.**

*Tumor assessment cycle: tumor efficacy assessment every 6 weeks (±7 days) at baseline and every 6 weeks (±7 days) during the study treatment period prior to initiation of therapy, and every 9 weeks (±7 days) after 48 weeks of the first study medication.Time interval between the 2 consecutive tumor assessments: 12 weeks + 14 days for the first 48 weeks after the first medication, and 18 weeks + 14 days for the first 48 weeks after the first medication.

6.4.1.3 OS Analysis

6.4.1.3.1 OS primary analysis

OS (months) = (date of death/date of final confirmation of survival - date of first administration of study drug + 1)/30.4375. In addition to the date of the event, the censored date is the date of final confirmation of the subject's survival: take the latest of all the dates of assessment, date of the visit, date of the administration of medication, date of the examination, etc., in the CRF.

When the PFS rejects the null hypothesis, it will detect an improvement in OS with 85% power at a two-sided significance level of 0.05 (HR=0.67). In the final analysis of the PFS, if the PFS rejects the null hypothesis, the PFS passes the test level to the OS, and the interim analysis for OS will be performed.

The primary analysis of OS is based on the mITT (FAS). Event/censored outcomes for OS will be summarized. The Kaplan-Meier method will be used to estimate the OS survival curves for each treatment group, providing 25% percentile, median, and 75% percentile OS, and the Brookmeyer-Crowley method will be used to estimate the 95% CI for the median OS.The Greenwood formula and log-log transformation will be used to calculate the OS rates and 95% CIs for the 12-month, 18-month, and 24-month periods. The Greenwood formula can be used to construct a standard normal distribution for between-group comparisons based on the z-statistic.

Comparison of OS survival functions between the two groups will be performed using stratified log-rank tests with actual stratification results. Stratification factors include ECOG score (0 vs. 1), baseline AFP level (<400 ng/ml vs. ≥400 ng/ml), presence or absence of macrovascular invasion and/or extrahepatic metastasis (no vs. yes), and the boundary value of the statistically significant level of superiority P-value for the OS interim and final analyses will be determined on the basis of the percentage of the actual number of events that occurred (for details, see Section 7 OS Interim and Final Analyses) . The stratified Cox proportional hazards model will be also used to calculate the risk ratio (HR) for OS and its 95% CI.

6.4.1.3.2 OS sensitivity analysis

Sensitivity analyses 1-4 will be taken in turn to demonstrate the robustness of the primary OS analysis. The changes in the sensitivity analysis relative to the main analysis will be as follows:

Based on mITT (FAS), sensitivity analyses 1-3 will be done for OS, OS (months) = (date of death/date of confirmation of last survival - date of first administration of study drug +1)/30.4375. Based on all randomized subjects in Phase III (ITT), sensitivity analysis 4 will be done for OS, OS (months) = (date of death/date of confirmation of last survival - date of randomization +1)/30.4375.

Sensitivity analysis 1: Stratified log-rank test using the planned stratification factors.

Sensitivity analysis 2: When subjects received a new anticancer treatment, focus on the treatment effect prior to that event and censore the OS at the date of receiving the new anticancer treatment.

Sensitivity analysis 3:

Using the RPSFT model and considering the "pure" efficacy of the experimental drug, for subjects in the sorafenib group receiving a new anticancer therapy with a PD-1/L1 inhibitor, estimate the time of survival that might have been observed in the absence of the new anticancer therapy with a PD-1/L1 inhibitor.

Let the survival time in the sorafenib group be T_i^{off}, survival time from receiving new anticancer therapy is T_i^{on} , then the overall survival time:

$$T_i = T_i^{off} + T_i^{on}$$

The RPSFT used the Accelerated Failure Time Model (AFT) to convert the observed survival time of subjects receiving the new anticancer therapy. T_i converted to a counterfactual survival time U_i , i.e., assuming the survival time that might have been observed for patients in the sorafenib group not receiving new anticancer therapy, and estimating efficacy based on the counterfactual survival time:

$$U_i = T_i^{off} + T_i^{on} \exp(\psi_0)$$

Also for patients not receiving new anticancer therapy there:

$$U_i = T_i$$

Parameter estimation will be performed using interval search (grid search), the optimal parameters will be selected to solve for ψ_0 , the conventional analysis will be performed after finding the counterfactual time.

Sensitivity analysis 4: Based on all randomized enrolled subjects (ITT) in phase III, the analysis model is the same as the primary analysis of OS.

6.4.2 Secondary Endpoint Analysis

Primary analysis population: the PFS and OS analyses will be based on mITT (FAS), ORR and DCR analyses will be based on RES, and DOR analyses will be based on the confirmed CR/PR population.

Phase II single-arm study part, based on statistical description, no between-group comparisons. For the phase III randomized controlled study portion, statistical inference will be performed on the basis of descriptive analysis, giving test statistics and p-values for between-group comparisons, etc.

6.4.2.1 Overall Survival (OS rate)

For the 12-month, 18-month, and 24-month OS rates, the survival rate and corresponding variability will be estimated based on the Kaplan-Meier method, and the Greenwood formula will be used to construct Z statistics for inter-group comparison.

6.4.2.2 Investigators Progression-free Survival (PFS) Assessed by RECIST v1.1

PFS (months) = (date of event/censoring for PFS - date of first administration of study drug + 1)/30.4375. For the primary analysis of PFS, the censoring rules are the same as in Table 6.1 for the primary analysis of PFS for the primary endpoint.

Event/censored outcomes for PFS will be summarized. PFS survival curves for each treatment group will be estimated by the Kaplan-Meier method, providing 25% quantile, median, and 75% quantile PFS, and 95% CIs will be estimated by the Brookmeyer-Crowley method.3-month, 6-month, 9-month, & 12-month PFS rates and their 95% CIs will be calculated using the Greenwood formula and log-log transformation. The Greenwood formula could be used to construct a standard normal distribution, and between-group comparisons could be made based on the z-statistic.

Comparisons of PFS survival functions between the two groups will be performed using stratified log-rank tests with actual stratification results. Stratification factors included ECOG score (0 vs. 1), baseline AFP level (<400 ng/ml vs. ≥400 ng/ml), and the presence of macrovascular invasion and/or extrahepatic metastases (no vs. yes), with a significance level of two-sided 0.05. Hazard ratios (HRs) for PFS and their 95% CIs will be also calculated using the Cox proportional hazards model based on the actual stratification.

6.4.2.3 BOR/ORR/DCR Analysis Assessed by BIRC and Investigators Based on RECIST v1.1

The definition of best overall response (BOR) before PD or before the use of a new anticancer drug is shown in Table 6.2. The results of tumor assessments after new anticancer drugs are not included in the assessment. Objective response rate (ORR) is defined as the proportion of subjects with a confirmed complete response (CR) or confirmed partial response (PR) as best overall response (BOR) before PD or before the use of a new anticancer drug. Disease control rate (DCR) is defined as the proportion of subjects with BOR of CR, PR, or SD.

The ORR and DCR analyses will be based on the RES analysis population, which is defined as subjects with measurable baseline tumor assessment at baseline. The number and percentage of subjects in the analysis population for ORR and DCR were summarized, and their 95% CIs were calculated using the Clopper Pearson method. For the comparison of ORR/DCR between groups, the stratified CMH test was used to calculate the rate difference (95% CI) and P value. The stratification factors included ECOG score (0 vs 1), baseline AFP level (<400 ng/ml vs ≥400 ng/ml), and the presence or absence of macrovascular invasion and/or extrahepatic metastasis (none vs yes).

Table 6.2 Best overall response (BOR) derivation rules

Assessment of efficacy	Final determination of BOR
CR at both first and follow-up efficacy assessments ≥ 4 weeks apart:<br>• First and follow-up assessment visits are adjacent<br>• First and follow-up assessment visits are not adjacent, with any intervening assessment of NE/CR	CR
BOR not CR with first assessment of PR and subsequent PR/CR with an interval of ≥ 4 weeks:<br>• First and follow-up assessments are adjacent<br>• First and subsequent assessments are not contiguous and 1 assessment of SD may exist in between<br>• Any number of intervening assessments can exist as NE/PR/CR	PR
BOR not for PR/CR:<br>• Presence of an assessment of SD/PR/CR and ≥35 days from first dosing	SD
Presence of BOR not being SD/PR/CR:<br>• First assessment of PD<br>• PD assessed within 2 consecutive assessment cycles* since last tumor assessment	PD
BOR is not PD/SD/PR/CR in cases that include, but are not limited to:<br>• No tumor assessment documented after the first administration of study drug	NE

Assessment of efficacy	Final determination of BOR
• PD after 2 consecutive assessment cycles* since the last swelling assessment	

*Tumor assessment cycles: every 6 weeks (±7 days) for tumor efficacy assessments at baseline and during the study treatment period prior to initiation of therapy, and every 9 weeks (±7 days) after 48 weeks of first study drug.Time interval between 2 consecutive tumor assessments: 12 weeks + 14 days for the first 48 weeks after the first dose of study drug, and after 48 weeks, take 18 weeks + 14 days.

6.4.2.4 BIRC and Investigator-assessed Duration of response (DOR) Analysis

The population analyzed for DOR is the confirmed CR/PR population. DOR (months) = (date of PFS event or censoring - date of first PR/CR imaging + 1)/30.4375.

Event/censored outcomes for DOR will be summarized. DOR survival curves for each treatment group will be estimated by the Kaplan-Meier method, providing 25% quantile, median, and 75% quantile DORs, and 95% CIs will be estimated by the Brookmeyer-Crowley method . The best overall response (BOR) derivation rule is the same as for ORR, and the censoring rule is the same as for the primary endpoint, PFS, for the primary analysis.

Comparison of the DOR survival function between the two groups is performed by stratified log-rank tests using actual stratification results. Stratification factors included ECOG score (0 vs 1), baseline AFP level (<400 ng/ml vs ≥400 ng/ml), and the presence of macrovascular invasion and/or extrahepatic metastasis (no vs yes), with a significance level of two-sided 0.05. Hazard ratios (HR) for PFS and their 95% CIs will be calculated using the Cox proportional hazards model based on the actual stratification as well.

6.4.2.5 Progression-free survival (PFS) Assessed by BICR Based on mRECIST

The analysis model is the same as the primary analysis for the primary endpoint PFS.

6.4.2.6 Objective response rate (ORR) Assessed by BICR Based on mRECIST

Analysis modeled as objective response rate (ORR) as assessed by BICR based on RECIST v1.1.

6.4.2.7 Duration of response (DOR) Assessed by BICR Based on mRECIST

Duration of response (DOR) as assessed by BICR based on RECIST v1.1 for analytic models.

6.4.2.8 Disease Control Rate (DCR) Assessed by BICR Based on mRECIST

Disease Control Rate (DCR) as assessed by analytic model same as BICR based on RECIST v1.1.

6.4.2.9 Quality of Life Conditions (EORTC QLQ-C30 and EORTC QLQ-HCC18 scales)

Descriptive statistics will be used to summarize the EORTC QLQ-C30 and EORTC QLQ-HCC18 scores for each domain at each assessment time point and to summarize the change from baseline in each domain score at each assessment. Plot the mean and average change in each domain score over time for both groups.

The 30 entries of the ORTC QLQ-C30 (V3.0) can be categorized into 15 domains, counting five functional domains (somatic, role, cognitive, emotional, and social functioning), three symptomatic domains (fatigue, pain, and nausea and vomiting), one domain of general health status/quality of life, and six single entries (each serving as a domain).The QLQ-HCC18 scales contain scores for fatigue, somatic Changes, Jaundice, Nutritional Changes, Pain, Fever, Sexual Life Changes, and Bloating for a total of 8 domains.

The EORTC QLQ-C30 (V3) and QLQ-HCC18 will be scored according to the scoring procedure of the EORTC QOL group. A rough score (RS, Raw Score) for each domain is obtained by summing the scores of the entries included in each domain and dividing by the number of entries included, i.e., RS= (Q 1+ Q 2 + ...+ Q n) / n. Q n is the nth question in the scale. To facilitate comparison, the rough scores will be converted to percentile standard scores (SS) using the method of polar deviation with the following formula: standard score (SS) = [1-(RS-1)/R] × 100 for the functional domain, and standard score (SS) = [(RS-1)/R] × 100 for the symptomatic domain and the domain of total health status. see Table 6.3. higher scores on the functional domain and the domain of total health status indicate that the functional status and the quality of life are better, and higher scores on the symptom domain indicate more severe symptoms. The specific criteria will be calculated in the last column of the table below.

Table 6.3 EORTC QLQ-C30 and QLQ-HCC18 Dimension Composition and Criteria Scoring Methods

Scale	Domain	Nature	Number of entries	Full Distance (R)	Rough Score Scoring Method (RS)	Standard Score (SS)
C30	Total health status		2	6	(Q29+Q30)/2	[(rs-1)/r] × 100
	Somatic Function	Functional	5	3	(q1+q2+q3+q4+q5)/5	[1-(RS-1)/R] x 100
	Role Function		2	3	(Q6+Q7)/2	
	Emotional function		4	3	(q21+q22+q23+q24)/4	
	Cognitive functioning		2	3	(Q20+Q25)/2	
	Social functioning		2	3	(Q26+Q27)/2	
	Fatigue	Symptomatic	3	3	(q10+q12+q18)/3	[(RS-1)/R]×100
	Nausea and vomiting		2	3	(Q14+Q15)/2	
	Pain		2	3	(Q9+Q19)/2	
	Shortness of breath		1	3	Q8	
	Insomnia		1	3	Q11	
	Loss of appetite		1	3	Q13	
	Constipation		1	3	Q16	
	Diarrhea		1	3	Q17	
	Economic difficulties		1	3	Q28	
HCC18	Fatigue	Symptomatic	3	3	(q45+q46+q47)/3	[(RS-1)/R]×100
	Physical changes	Symptomatic	2	3	(Q33+Q35)/2	
	Jaundice	Symptomatic	2	3	(Q36+Q37)/2	
	Nutritional changes	Symptomatic	5	3	(q31+32+q42+q43+q44)/5	
	Pain	Symptomatic	2	3	(Q38+Q39)/2	
	Fever	Symptomatic	2	3 (Q40+Q41)/2	(Q40+Q41)/2	
	Bloating	Symptomatic	1	3	Q34	
	Change in sex life	Symptomatic	1	3	Q38	

6.4.3 Subgroup Analysis

Prespecified subgroup analyses of the primary endpoints PFS (BICR, RECIST v1.1), OS, and forest plots of HR for each subgroup will be provided , analyzed as in Section 6.4.1 Primary Endpoint Analyses, except that the model no longer incorporates other stratification factors for between-group comparisons within the strata of the subgroups. The primary subgroups are selected, as exemplified below:

Baseline ECOG score (0 vs. 1);

Age (<65 vs. ≥65);

Gender (male vs. female);

Extrahepatic metastases and/or macrovascular invasion (yes vs. no);

Presence of extrahepatic metastases (yes vs. no);

Presence of large vessel invasion (yes vs. no);

BCLC staging (B (intermediate stage) vs. C (advanced stage));

Child-Pugh classification of liver function (5, 6, 7);

Baseline AFP level (<400 ng/ml vs. ≥400 ng/ml);

History of viral hepatitis (HBV infection, HCV infection, no HBV/HCV infection);

Previous localized hepatic therapy (yes vs. no);

PD-L1 expression level (CPS>1 vs. CPS≤1, TPS>1% vs. TPS≤1%).

6.4.4 Integrated Summary of Effectiveness

The PFS and OS will be analyzed by combining the phase II and phase III SCT-I10A+SCT510 groups of subjects.

For PFS, PFS curves will be estimated by the Kaplan-Meier method to provide the 25% quartile, median, and 75% quartile PFS and PFS rates, and the Brookmeyer-Crowley method will be used to estimate the 95% CI for the median PFS, and Greenwood's formula and log-log transformations will be used to calculate the 3-month, 6-month, 9-month, and 12-month PFS rates.

The primary endpoint OS in phase III will be also analyzed in a combined analysis with the efficacy endpoint OS in phase II, and the analysis model will be the same as PFS.

6.5 Safety Analysis

6.5.1 Prior and Combined Drug/Non-drug Therapy

Prior medications, combined medications, prior nonpharmacological treatments, and combined nonpharmacological treatments will be summarized separately. Analyses of specific

drugs and treatments will be summarized as coded in the latest version of WHODrug. Non-pharmacological treatments will be summarized according to the PT after coding in the latest version of MedDRA. The order of drug presentation in the summary table will be arranged in descending order according to the incidence of the trial group, and the number of cases and percentages will be summarized.

For cases where the stage of drug or non-drug treatment could not be determined due to missing or incomplete dates, "combined medications" or "combined non-drug treatments" will be included in the statistics.

6.5.2 Study Drug Exposure and Compliance

Drug exposure and compliance will be calculated separately for each group with the following derivation rules:

Exposure time

- Actual exposure time (weeks):
  - For SCT-I10A+SCT510 group, take (date of last dose - date of first dose + 21)/7
  - Sorafenib group, take (date of last dose - date of first dose + 1)/7
- Actual dosing cycle = number of cycles from first dosing to last dosing according to actual CRF records

Dose and Dose Intensity

- Cumulative Actual Dose (mg) = sum of the actual doses administered in each cycle (sum the "Actual Doses Administered" on the Study Dosing Page)
- Cumulative Planned Dose (mg) = Weekly Planned Dose (mg/week) (as specified in the protocol) * Planned Duration of Administration (weeks)
- Planned Duration of Administration (weeks) = Actual Exposure (weeks)
- Actual dose intensity (mg/week) = Cumulative actual dose (mg)/exposure time (weeks)
- Relative Dose Intensity = Actual Dose Intensity (mg/week) / (protocol specified) Weekly Planned Dose (mg/week)
- (Protocol-specified) weekly planned dose (mg/week) = planned dose administered in the first dosing cycle/3

Compliance

- Compliance (%) = Cumulative Actual Dose (mg) / Cumulative Planned Dose (mg) * 100%

6.5.3 Adverse Events

Adverse events other than immune-related adverse events (irAE) can be considered for analysis based on either of the following two types:

- Based on investigator determination
- Based on re-determination by the sponsor's medical team after communication with the investigator and consideration of scientific validity and rigor
  - After coding using MedDRA, for SOC/PTs with similar medical significance, sponsors may combine groups appropriately based on medical logic.
  - For AEs with a ≥ level 3 investigator determination of causality of "probably unrelated" or an AE with a final preferred term (PT) of "death," the sponsor determines the relevance to the study drug on a case-by-case basis, confirming that each "probably unrelated" and "probably unrelated" AE is not a case. The sponsor determines the relevance to the study drug on a case-by-case basis, identifies the basis for each "probably not relevant" and "definitely not relevant" AE, and documents and provides the details to the regulatory authority.

See Section 6.5.3.5 Immunization-Related Adverse Events for details on the process of analyzing irAEs.

Exposure adjusted incidence rate (EAIR) analysis of adverse events will be handled on an as-needed basis.

6.5.3.1 Standardization of terminology

Adverse events will be coded in MedDRA latest version and graded according to CTCAE v5.0.

After coding adverse events using MedDRA, sponsors can merge terminology for SOCs/PTs with similar medical significance. Rules for merging AE terms given by professional medical judgment will be given in a separate document.

6.5.3.2 Adverse events during treatment

The analysis of adverse events will be based on treatment-emergent adverse events (TEAE), with the time frame for TEAE taken from the start of the first dose to 90 days after the last dose or before starting any new anticancer therapy.

The number of cases and incidence of TEAEs in each category will be summarized by dosing situation (SCT-I10A, SCT510, SCT-I10A and/or SCT510, sorafenib), e.g., all TEAEs,

TEAEs with an incidence rate of >=10%, Adverse Events of Special Interest (AESIs), SCT510 Specific Adverse Events (Bevacizumab Instructions) Adverse Events of Special Interest, a list of PTs is given in a separate document), TEAEs leading to suspension of SCT-I10A and/or SCT510 infusion, TEAEs leading to suspension of study drug, TEAEs leading to downward adjustment of sorafenib dosage, TEAEs leading to discontinuation of study drug, Serious Adverse Events (SAEs), TEAEs leading to death.

TEAEs in the above categories will be similarly summarized by SOC and PT (equivalently notated as SOC/PT), and combined with clinical needs will be summarized by severity as rated by CTCAE.

When calculating the incidence of each category of adverse events by SOC and PT, if a subject had multiple occurrences of an adverse event under the same SOC/PT, it is counted as one case under that SOC/PT, and the severity level is taken as the most severe level.The order of presentation of the AEs is first in descending order by the incidence of the SOC in all the subjects, and then in descending order by the incidence of the PT within the SOC.

6.5.3.3 TEAE related to study drug

A professional medical judgment will be made based on a comprehensive analysis of the characteristics of the underlying disease, the mechanism of action of the drug, and the accumulated AE data. Adverse events (AEs) included in the study will be judged for causality using a "five-point scale", with the results categorized as "definitely related", "probably related", "probably not related", "probably not related", "probably not related", "probably not related", "probably not related", "probably not related", and "probably not related", "Possibly not related", "Definitely not related", and "Undetermined". AEs judged as "definitely relevant", "possibly relevant" and "undecided" will be taken by the sponsor as TEAEs related to the study drug, also known as adverse drug reaction (ADR). also known as adverse drug reaction (ADR).

For AEs with a causal relationship of "probably unrelated" as determined by the investigator of level ⩾3, the sponsor's medical team, after communicating with the investigator, will determine the relevance of the study drug on a case-by-case basis, and confirm that each "probably unrelated" and "definitely unrelated" AE has been determined to be "probably unrelated" and "definitely unrelated" to the study drug. The sponsor medical team communicated with the investigator to determine the relevance to the study drug on a case-by-case basis, confirmed the basis for each "probably unrelated" and "definitely unrelated" AE, and documented and provided the details to the regulatory authority; AEs determined to be "probably unrelated" and "definitely unrelated" will be excluded from the ADR.

In the event of a death, actions will be taken in accordance with regulatory and protocol requirements. The term "death" is not an AE per se, and the cause of death should be recorded as an AE, but because the cause of death could not be fully determined in some of the deaths, the preferred term (PT) "death" is recorded. For these AEs, the sponsor's medical team communicated with the investigator to determine the relevance of the investigational drug in each case, identified the basis for each "probably unrelated" and "definitely unrelated" AE, and documented it in detail and provided it to the regulatory authority. AEs determined to be "probably not relevant" and "definitely not relevant" may not be included in the ADR.

The summary categories for TEAE (TRAE/ADR), as exemplified in the TEAE above, will be presented as relevant to SCT-I10A, SCT510, SCT-I10A and/or SCT510, sorafenib, respectively.

6.5.3.4 Death, Serious Adverse Events

The timeframe of data for SAE, death is the same as for TEAE, i.e., taken from the start of the first dose to 90 days after the last dose or before initiation of any new anticancer therapy.

The number of cases and incidence of SAEs in each group will be summarized by SOC, PT, and correlation, and correlation will be presented as with SCT-I10A, SCT510, SCT-I10A, and/or SCT510, sorafenib, respectively, and detailed in the list for each subject. Relative to SAE, deaths will be similarly summarized.

6.5.3.5 Immune-Related Adverse Events

An immune-related adverse event (irAE) is defined as an adverse drug reaction at all levels judged to be causally related to an immune mechanism in clinical trials of anticancer drugs/treatments.

On the overall medical judgment of SCT-I10A products, the judgment process of irAE will be formulated by combining the definition of study protocol, data collection and other considerations. The sponsor medically categorizes irAEs based on domestic and international guidelines for toxicity management related to immune checkpoint inhibitors and clinically relevant data for similar products, as well as the occurrence of irAEs during the clinical study of SCT-I10A. irAE determination process and irAE medical categorization will be given in a separate document.

The number of cases and incidence of irAEs in SCT-I10A will be summarized by category, e.g., all irAEs, irAEs leading to suspension of drug, irAEs leading to discontinuation of drug, irAEs leading to withdrawal from the study, and irAEs leading to death.

The number of cases and incidence of irAEs will be summarized by irAE subcategory, measure, and severity. The time to first occurrence, duration, and regression of each type of irAE will be described. For irAEs categorized as "systemic glucocorticoid therapy", the starting dose and duration of administration of irAEs in the high-dose group are additionally considered:

- irAE to first occurrence (months) = (date of first event - date of first dose +1)/30.4375
- Duration of irAE (months) = (date of recovery or recovery with sequelae - date of occurrence + 1)/30.4375
- The outcome value is taken as the outcome of the latest AE.

6.5.4 Laboratory Tests

For quantitative laboratory tests, statistics of measures will be used to descriptively summarize the observations and change from baseline across visits for each group. For qualitative laboratory tests, a statistical descriptive summary of categorical indicators is used to summarize the results at each visit for each group.

For all laboratory tests, a cross-tabulation is used to summarize their assessment of clinical significance before and after treatment, with baseline results used before treatment and the most severe assessment at each visit after treatment, in the order of severity: abnormal clinically significant > abnormal not clinically significant > normal > not examined, or positive > negative > not examined, if negative and positive judgments are available only. Judged as not checked only if the indicator has no test results after treatment. The number and percentage of subjects in each cell of the crosstab will be calculated, and the number of subjects with a baseline and at least one post-baseline assessment result is used as the denominator when the assessment is excluded from "not investigated".

For each laboratory test indicator, CTCAE severity classes will be summarized by CTCAE severity class, and a cross-tabulation format is used to summarize the most severe CTCAE class at baseline and post-treatment.

Give listings showing the laboratory test results for all subjects. For subjects for whom an abnormal laboratory test was observed, a list of all results for that test is shown. Pregnancy is listed for positive subjects.

6.5.5 Vital Signs, Physical Examination, and Other Safety Assessments

6.5.5.1 Vital Signs

Vital signs such as weight (kg), systolic and diastolic blood pressure (mmHg), pulse rate

(beats/minute), body temperature (˚C), and respiratory rate (breaths/minute). The following summary is provided:

Summary of observations and change from baseline values for each vital sign by visit;

Summarize the assessment of the clinical significance of each vital sign observation before and after treatment by cross-tabulation;

Provide a listing of vital signs categorized by subject.

6.5.5.2 Physical Examination

The number and percentage of subject cases will be summarized by physical examination result category (normal, abnormal without clinical significance, and abnormal with clinical significance).

A qualitative description of the change from baseline and post-treatment physical examination (based on clinician judgment) relative to baseline will also be provided in the form of a cross-tabulation, with the most severe post-baseline result being analyzed and the number and percentage of cases calculated.

Provide a listing of physical examination results by subject.

6.5.5.3 12-ECG

The number of cases and percentages of subjects will be summarized by electrocardiogram (ECG) category (normal, abnormal without clinical significance and abnormal with clinical significance).

A qualitative description of the change in 12-ECG (as judged by the clinician) at baseline and post-treatment relative to baseline will be provided in the form of a cross-tabulation, with the most severe post-baseline result being analyzed and the number and percentage calculated.

A list of ECG results by subject will be provided.

6.5.5.4 ECOG Physical Status

The number of cases and percentages of subjects will be summarized by ECOG score category.

A qualitative description of the change in ECOG physical status scores at baseline and post-treatment relative to baseline will be provided in the form of a cross-tabulation, with the most severe post-baseline results being analyzed and the number and percentage calculated.

A list of ECOG physical status score results by subject will be provided.

6.6 PK and Immunogenicity Analysis

PK analysis and immunogenicity analysis will be detailed in a separate analysis plan.

6.7 Treatment of Missing Data

If other scientifically valid ways of filling in the missing data other than those described below are undertaken, they will be documented in detail in the data description document for the BLA submitted data set.

6.7.1 Missing Date

Pre-existing dates:

Year missing, not filled; day and month missing, month UN taken as 1 month, day UN taken as 1 day filled, with the filled date value compared to the informed consent date, whichever is earlier.

Date of start of new anticancer therapy:

Missing should be avoided. Year is missing and will not be filled. Day and month are missing, compare incomplete date to EOT date, or date of last dose if no EOT record. Month missing, if the year of the new anticancer therapy treatment start date is less than the year of the EOT/last dose date, fill to the last day of the year of the new anticancer therapy, equal to fill to the EOT/last dose date +1, greater than fill to January 1; day missing the same way, compare with the year and month of the EOT/last dose date, less than take the last day of the month of the new antibody, equal to fill to the EOT/last dose date +1, greater than that it is filled to the 1st day of the month. The value of the filled date is compared with the date of death, whichever is earlier.

Date of death:

Missing should be avoided. For subjects where death is recorded but the date of death is missing, the year is missing and is not filled; for days and months, the month UN is filled by taking 1 month and the day UN is filled by taking 1 day, and the filled date value is taken to be greater than the date of the last confirmation of survival, and vice versa to be taken to the date of the last confirmation of survival + 1.

AE Date:

- Start date: year is missing, not filled; day and month are missing, month UN is taken as 1 month, day UN is taken as 1 day filled, and the filled date value is taken as the greater value when compared to the date of first administration of study drug.

- End date: year is missing and not filled; day and month are missing, month UN is taken as December and day UN is filled as the last day of the month. For AEs resulting in death, if the year of the end date is missing, it is filled as the date of death; if the month or day of the end date is missing, it is filled according to the rules and compared with the date of death, taking the earlier of the two as the end date.
- After filling, compare with the date of death, take the lesser value, and if the start date is later than the end date, make the start date equal to the end date.

Beginning and ending dates of other events:

Year is missing, not filled; day and month are missing, month UN is taken as 1 month, day UN is taken as 1 day to be filled, and after filling, it is compared with the date of death, taking the lesser value, and if the start date is later than the end date, the start date is made equal to the end date.

6.7.2 Efficacy/Safety Indicator Data Missing

The rules for deletion of PFS, DOR, and OS, and the treatment of missing values for ORR are described in the corresponding section of Section 6.4, and the date of disease progression or date of death are avoided as much as possible.

For adverse events, if the stage could not be determined due to missing or incomplete dates, the TEAE is used as a participant in the statistics, and missing severity is treated as "grade 3", and missing correlation with SCT-I10A and/or SCT510, sorafenib, is treated as "undetermined". " treatment.

For cases where the stage of drug or non-drug treatment could not be determined due to missing or incomplete dates, they will be counted as "combined medication" or "combined non-drug treatment".

If the stage of medical history could not be determined due to missing or incomplete dates, "concomitant disease" is used.

7 Interim and Final Analysis of OS

The study has dual primary endpoints, PFS (BICR, RECIST v1.1) and OS, which will be analyzed using a fixed-sequential procedure to control for the total familywise error rate (FWER) at two-sided 0.05 (0.025 one-sided).

The final analysis of the PFS is expected to be performed when approximately 252 PFS events are observed, at a two-sided 0.05 level of significance. At the final analysis of the PFS, the study will be terminated if the null hypothesis of PFS is not rejected; in contrast, if the PFS

primary analysis result is significant, the two-sided alpha of 0.05 will be passed to the OS.

One interim analysis (IA) with one final analysis (FA) will be performed on OS. In the final analysis of PFS, if PFS rejects the null hypothesis, a interim analysis of OS will be performed.

The P-value boundary value for the OS interim analysis will be determined by approximating the O'Brien-Fleming boundary according to the Lan-DeMets method, and the corresponding statistical significance level will be adjusted according to the number of OS events that actually occurred in the interim analysis as shown in Table 7.1 below.

The final analysis of PFS, the interim analysis of OS (collectively referred to as the interim analysis), will be programmed and processed in SAS version 9.4 or higher based on the pre-determined number of progression-free survival (PFS) events assessed by the BICR, with a data cut-off date of November 02, 2023 determined:

- For data recorded per visit, intercept all visit data no later than the cutoff date. For tumor imaging assessment data, the earliest scan date from the same visit no later than the cutoff date is included in all data from this visit;
- For data not recorded at the same visit, such as prior medical history, prior treatment, prior and combined drug therapy, prior and combined non-drug therapy, adverse events, subsequent anticancer therapy, study summaries, and death records, incorporate all data with a start date no later than the cutoff date. When the relationship with the cutoff date could not be determined because the start date is missing, it is included in the analysis.

Table 7.1 OS Interim and Final Analysis Boundaries

OS IA number of events and information ratio	IA significant level	FA significant level
127 (50.2%)	0.00312	0.04898
130(51.4%)	0.00353	0.04885
140 (55.3%)	0.00517	0.04834
150 (59.3%)	0.00721	0.04772
160 (63.2%)	0.00965	0.04699
165 (65.2%)	0.01102	0.04659
166 (65.6%)	0.01131	0.04650
167(66.0%)	0.01160	0.04642
168 (66.4%)	0.01190	0.04633
169(66.8%)	0.01220	0.04625
170 (67.2%)	0.01250	0.04616
171 (67.6%)	0.01281	0.04607
172(68.0%)	0.01312	0.04598
173 (68.4%)	0.01343	0.04589
174 (68.8%)	0.01375	0.04580
175 (69.2%)	0.01408	0.04570
176(69.6%)	0.01440	0.04561
177(70.0%)	0.01474	0.04552
178(70.4%)	0.01507	0.04542
179(70.8%)	0.01541	0.04533
180 (71.1%)	0.01575	0.04523

8 Major Changes in SAP Relative to the Protocol

Changes	Study Protocol (V2.0 /2021-04-20)	Statistical Analysis Plan (V1.0 /2023-11-15)
Analysis set	Undefined ITT population	Definition of ITT population in Phase II/Phase III: Phase II for all enrolled subjects; Phase III for all randomized subjects
	Safety Analysis Set (SS): includes all subjects who received at least one study drug with at least one post-dose safety assessment	Safety Analysis Set (SS): includes all subjects who received at least one dose of study medication
	Phase II baseline and demographic analyses based on SS	Phase II baseline and demographic analysis based on mITT (FAS)
	Undefined Response-Evaluable set (RES)	Add Response-Evaluable set (RES): a subset of the full analysis set, taken from all subjects with an evaluable baseline target lesion present
Statistical assumptions	Null hypothesis H_0: HR ≥1 on PFS and OS; alternative hypothesis H_1: HR<1	Both the original and alternative hypotheses for the primary endpoint PFS and OS analyses can be expressed as corresponding survival functions: H_0 : S_T (t) ≤ S_C (t) vs H_1 : S_T (t) ≠ S_C (t)
ORR/DCR analysis method	The exact probability (or chi-square test) method is used to compare the between-group differences in ORR and DCR between the two groups	The stratified CMH test is used to calculate the rate difference between the groups and confidence intervals, and the stratification factors are taken as the ECOG score (0 vs. 1), the baseline AFP level (<400 ng/ml vs. ≥400 ng/ml), and the presence of macrovascular invasion and/or extra-hepatic metastasis (no vs. yes).

9 References

[1] O'Brien PC, Fleming TR. A Multiple Testing Procedure for Clinical Trials. biometrics.1979; 549-556

[2] ICH. E9 Statistical Principles for Clinical Trials. 1998

[3] FDA. Guidance for Industry Integrated Summaries of Effectiveness and Safety: Location Within the Common Technical Document. 2009

[4] Herson J. Data and Safety Monitoring Committees in Clinical Trials. portland:Taylor & Francis Group, 2016.

[5] ICH. E9(R1): Addendum on Estimands and Sensitivity Analysis in Clinical trials to the Guideline on Statistical Principles for Clinical Trials. 2019

[6] FDA. Adaptive Design Clinical Trials for Drugs and Biologics Guidance for Industry. 2019

[7] Center for Drug Evaluation of NMPA. Technical Guidelines for the Preparation of Integrated Summary of Safety for NDA of Innovative Anticancer Drugs. 2020

[8] Center for Drug Evaluation of NMPA. Guidelines for Clinical Trial Data Monitoring Committee (for Trial Implementation). 2020

[9] Center for Drug Evaluation of NMPA. Guidelines for Comprehensive Analysis of the Effectiveness of Clinical studies on Drugs (for Trial Implementation). 2021

[10] Center for Drug Evaluation of NMPA. Guideline on Data Management Plan and Statistical Analysis Plan of Drug Clinical Trials. 2021

[11] Center for Drug Evaluation of NMPA. Technical Guidelines for the Evaluation of Immuno-Related Adverse Events in Anticancer Therapy. 2022

[12] Center for Drug Evaluation of NMPA. Guiding Principles for Blinding of Drug Clinical Trials. 2022

[13] Center for Drug Evaluation of NMPA. Guiding Principles for Sample Size Estimation in Drug Clinical Trials (Draft for Comment). 2023
